# The dual roles of uterine monocytes in regulation of tissue homeostasis throughout reproductive cycles in health and disease

**DOI:** 10.1038/s41590-026-02651-y

**Published:** 2026-09-08

**Authors:** Abdullah Shahzad, Olivia Moran, Yousef Alebrahim, Stefano Colombo, Livia Lacerda Mariano, Olivia Shorthouse, Hannah Morgan, Daxina Bhatt, Nicholas Scott, Felicity K. Hunter, Catriona Laverty, Peter Ruane, Gerard Graham, Isabel Paterson, Navneet Kaur, Zohra Amin, Mariam Lokman, I-Hsuan Lin, Syed Murtuza-Baker, Michael Haley, Ian J. Donaldson, Jenny Myers, Daniel R. Brison, John D. Aplin, Molly A. Ingersoll, Laurence Pearmain, Lamiya Mohiyiddeen, Elizabeth R. Mann

**Affiliations:** 1 Lydia Becker Institute of Immunology and Inflammation, Faculty of Biology, Medicine and Health, University of Manchester, Manchester, UK; 2 Division of Infection, Immunity and Respiratory Medicine, School of Biological Sciences, Faculty of Biology, Medicine and Health, Manchester Academic Health Science Centre, University of Manchester, Manchester, UK; 3 St Mary’s Hospital, Manchester University NHS Foundation Trust, Manchester, UK; 4 Division of Diabetes, Endocrinology and Gastroenterology, School of Medical Sciences, Faculty of Biology, Medicine and Health, Manchester Academic Health Science Centre, University of Manchester, Manchester, UK; 5 Department of Immunology, Institut Pasteur, Paris, France; 6 Maternal and Fetal Health Research Centre, Division of Developmental Biology and Mediciner, St Mary’s Hospital, University of Manchester, Manchester, UK; 7 Chemokine Research Group, Institute of Infection, Immunity and Inflammation, College of Medical, Veterinary and Life Sciences, University of Glasgow, Glasgow, UK; 8 Bioinformatics Core Facility, Faculty of Biology, Medicine and Health, University of Manchester, Manchester, UK; 9 Bioimaging Core Facility, Faculty of Biology, Medicine and Health, University of Manchester, Manchester, UK; 10 North West Lung Centre, Wythenshawe Hospital, Manchester University NHS Foundation Trust, Manchester, UK

**Keywords:** Mucosal immunology, Innate immunity

## Abstract

Inflammation occurs during the recurring cycles of tissue degradation, repair and remodeling in the uterus, but the mechanisms by which the immune system contributes to uterine tissue integrity are unclear. We reveal that uterine monocytes have dual roles in both tissue remodeling and inflammation, depending on the reproductive cycle stage. Circulating inflammatory monocytes infiltrated the uterus before their differentiation into proinflammatory or pro-repair macrophages, depending on interferon-γ and transforming growth factor-β, respectively. Human monocyte numbers peaked before the window of implantation, when an inflammatory environment is required for embryo implantation. Uterine monocytes exhibited cytotoxic properties, regulating epithelial cell apoptosis. Bioinformatic analyses of receptor–ligand interactions indicated that monocytes dominated interactions between myeloid cells and fibroblasts. These pathways were disrupted in uterine fibrosis, marked by excess inflammatory monocytes or macrophages and a loss of rhythmicity through reproductive cycles. These data reveal a key role for monocytes and their progeny in the regulation of uterine tissue homeostasis.

## Abstract (web summary)

Mann and colleagues reveal key roles for monocytes in replenishing the subsets of uterine macrophages involved in tissue remodeling and inflammation. These processes maintain tissue homeostasis in healthy reproductive cycles, but are disrupted in reproductive disease.

## Main

The lining of the uterus (endometrium) must maintain tissue integrity during the repeated cycles of shedding and regeneration that occur during the menstrual cycle^1,2^. The endometrium has a distinct structure, with glands bounded by epithelial cells within stroma containing fibroblasts, immune cells and extensive vasculature. Epithelial cells also provide a barrier between the endometrium and the inner lumen. After cyclical episodes of proliferation and differentiation of the epithelium into secretory cells and fibroblasts into decidual cells, if pregnancy does not occur, the inner layer of endometrium breaks down and is shed. A period of repair and tissue regeneration then indicates the start of the next cycle^3^. Abnormalities in these processes can lead to infertility, miscarriage or poor pregnancy outcomes, but our understanding of the mechanisms underlying impaired reproductive performance is remarkably limited, reflecting underfunding and low prioritization of women’s health.

Macrophages are essential for tissue remodeling, regeneration and repair in other organs^4^ and their importance in the uterus is evidenced by CSF1-null female mice exhibiting reproductive defects^5^. Macrophages have long been proposed to contribute to the tissue breakdown and repair processes that occur in the uterus during the menstrual cycle. Endometrial macrophages increase in number during the secretory phase of the cycle^6^, but numbers are also high during menstruation, when they contribute to the breakdown of extracellular matrix (ECM), clearance of debris and initiation of repair^7,8^. Inflammation is thought to be required for embryo implantation^9,10^ at the mid secretory phase of the cycle in humans, with the proposal that the inflammatory milieu is provided by myeloid cells^11^. However, prolonged or skewed macrophage activation can lead to chronic inflammation, structural defects and/or scarring (fibrosis)^4^. Thus, tight regulation of these processes is critical and it remains unclear how endometrial macrophages can balance their roles in inflammation and physiological tissue remodeling. Single-cell studies of the endometrium have revealed distinct populations of macrophages with proinflammatory or tissue-remodeling features^12–16^, but the origins and in vivo roles of these subsets are unknown.

Here we reveal key roles for monocytes in replenishing the subsets of uterine macrophages involved in tissue remodeling and inflammation. These processes maintain tissue homeostasis in healthy reproductive cycles, but are disrupted in reproductive disease.

## Results

### Monocytes in the uterus are dynamic through reproductive cycles and continually replenish the macrophage pool

**Fig. 1:**
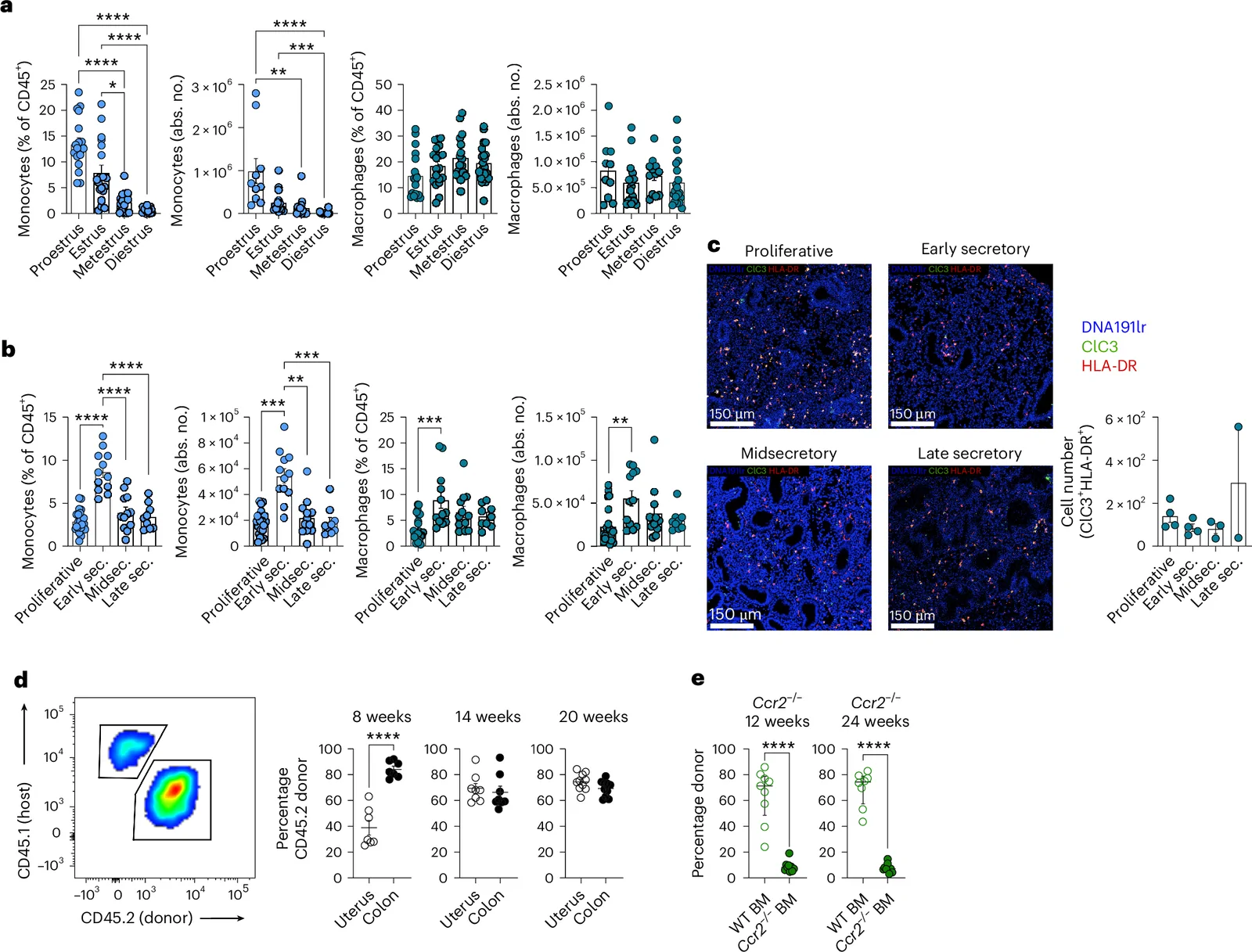
Monocytes and macrophages in the murine and human uterus. **a**,**b**, Flow cytometry summary graphs demonstrating proportions (of total CD45^+^ cells) and absolute numbers of monocytes and macrophages at different reproductive cycle stages within total digested mouse uteri and digested human endometrial biopsies (per mg of tissue for absolute numbers) (**a**) (the Kruskal–Wallis test was applied in all cases; proestrus, *n* = 18; estrus, *n* = 19’ metestrus, *n* = 17; and diestrus, *n* = 23) (**b**) (one-way analysis of variance (ANOVA) analysis was performed in all cases; proliferative, *n* = 22; early secretory, *n* = 12; mid secretory, *n* = 12; and late secretory, *n* = 9). **c**, Imaging mass cytometry pictures demonstrating ClC3 and HLA-DR staining in sections taken from endometrial biopsies at different stages of the menstrual cycle. The images represent five different regions of interest (ROIs) within one sample and 13 different biological samples. The summary graph shows the numbers of cells co-expressing HLA-DR and ClC3 at different stages of the menstrual cycle. Kruskal–Wallis test was applied: proliferative, *n* = 4; early secretory, *n* = 4; mid secretory, *n* = 3; and late secretory, *n* = 2. **d**, Example flow cytometry gating plot identifying host (CD45.1) versus donor (CD45.2) macrophages (pre-gated on CD45^+^live Ly6G^−^SigF^−^CD64^+^CD11b^+^Ly6C^−^ cells) from BM chimeric mice, and summary graphs showing proportions of total macrophages comprising donor (CD45.2) cells at various timepoints after irradiation. In all cases, the results were normalized to chimerism in blood monocytes. A paired, two-tailed Student’s *t*-test was applied in all cases: 8 weeks, *n* = 7; 14 weeks, *n* = 8; and 20 weeks, *n* = 10. **e**, Summary graphs showing proportions of macrophages comprising donor cells recruited from the bloodstream at 12 and 24 weeks post-irradiation when recipient mice were replenished with WT versus *Ccr2*^*−/−*^ BM. In all cases, the results were normalized to chimerism in blood neutrophils: 12 weeks: the unpaired two-tailed Mann–Whitney *U*-test was applied (mice replenished with WT BM (*n* = 9); mice replenished with *Ccr2*^*−/−*^ BM (*n* = 9)); at 24 weeks: an unpaired Student’s *t*-test was applied (mice replenished with WT BM (*n* = 8); mice replenished with *Ccr2*^*−/−*^ BM (*n* = 9)). In all cases, at least two independent experiments were carried out. For one-way ANOVA and Kruskal–Wallis tests, adjusted *P* values were reported from Tukey’s and Dunn’s post hoc tests, respectively: ^*^P < 0.05, ^**^P < 0.01, ^***^P_adj_ < 0.001, ^****^P < 0.0001. Data are presented as the mean ± s.e.m. in **b** and **d** and as the median ± interquartile range (IQR) in **a**, **c** and **e**. abs.no., absolute number; midsec., mid secretory; sec., secretory. Source data

**Extended Data Fig. 1:**
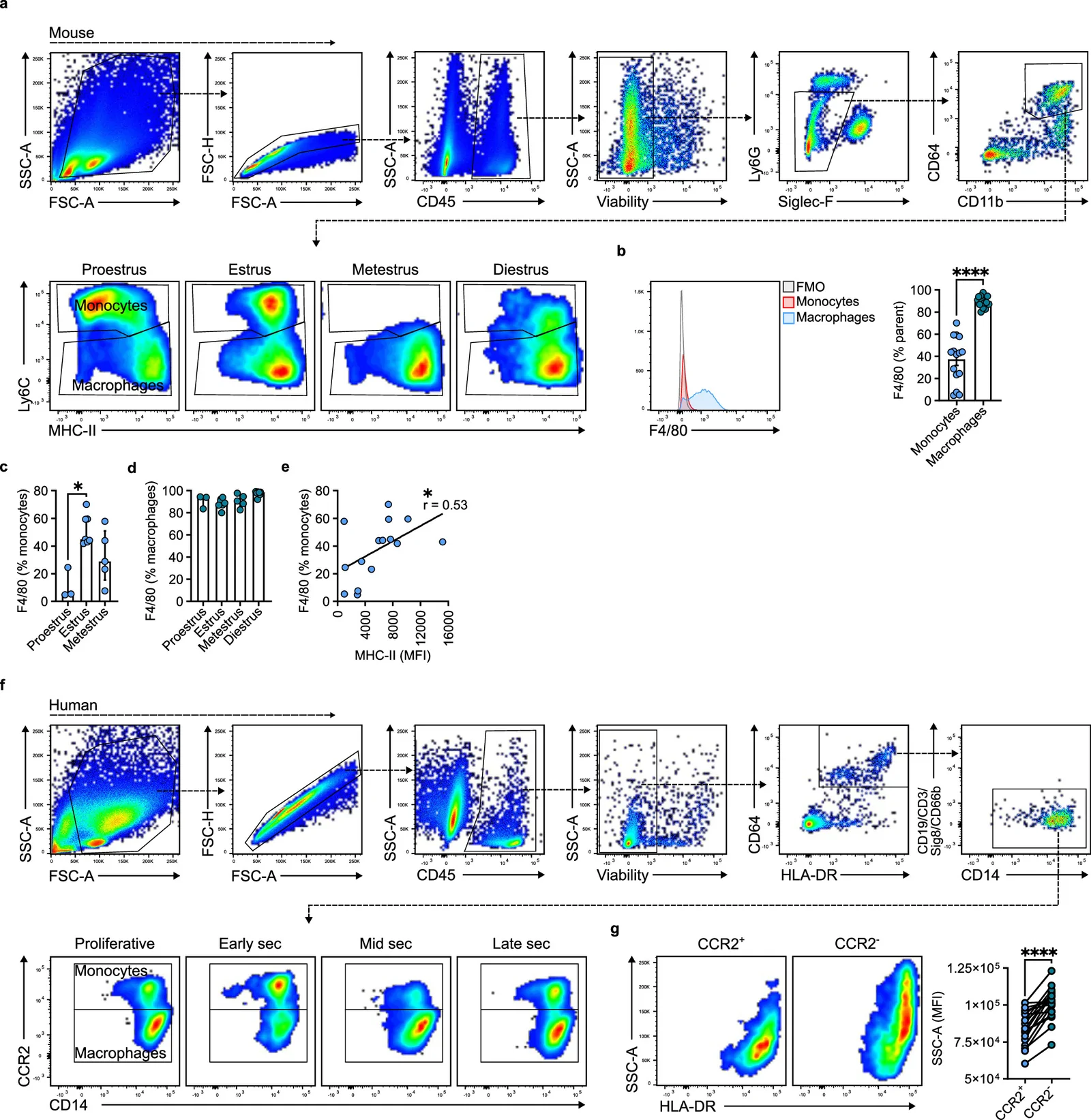
Flow cytometry gating strategy for uterine monocytes and macrophages in mice and humans. Flow cytometry gating strategy for **a**) mouse monocytes and macrophages at diestrus, proestrus, estrus and metestrus stages of the estrous cycle. **b**) Example staining for F4/80 expression on murine monocytes and macrophages and summary graph showing proportions of total uterine monocytes and macrophages (n = 15 in both cases) expressing F4/80. Two-tailed unpaired t-test was applied. **c**) Summary graph demonstrating proportions of monocytes expressing F4/80 at different estrous cycle stages (proestrus: n = 3, estrus: n = 7, metestrus: n = 5). Kruskal-Wallis test was applied. **d**) Summary graph demonstrating proportions of macrophages expressing F4/80 at different estrous cycle stages (proestrus: n = 3, estrus: n = 7, metestrus: n = 5, diestrus: n = 9). **e**) Summary graph demonstrating correlation of F4/80 expression (% of monocytes) with levels of MHC class II as assessed by mean fluorescence intensity (n = 15). Spearman’s rank correlation coefficient was carried out. **f**) Flow cytometry gating strategy for human uterine monocytes and macrophages at proliferative, early-, mid- and late-secretory stages of the menstrual cycle. **g**) Example flow cytometry staining showing side-scatter assessed by mean fluorescence intensity of CCR2^+^ and CCR2^−^ cells, pre-gated on CD45^+^ live CD3^−^CD19^−^CD66b^−^Sig8^−^CD64^+^HLA-DR^+^ cells, and summary graphs demonstrating side scatter values comparing CCR2^+^ and CCR2^−^ cells (n = 20). Two-tailed paired t-test was applied. In all cases, at least two independent experiments were carried out. For Kruskal-Wallis tests adjusted p values are reported from Dunn’s *post hoc* tests. *p < 0.05, **p < 0.01, ***p < 0.001, ****p < 0.0001. Data are presented as mean±SEM in **b**. Data are presented as median±IQR in **c**, **d**. Source data

Macrophage numbers in the uterus fluctuate during reproductive cycles^17,18^, but overlapping cell-surface proteins used to identify monocytes and macrophages makes them difficult to distinguish. Here we have defined gating strategies to identify monocytes and macrophages in the uterus in mice (Extended Data Fig. 1a) and humans (Extended Data Fig. 1f). In both species, monocytes rather than macrophages change in number dramatically across the reproductive cycle (Fig. 1a,b). In mice, we distinguished monocytes as Ly6C^hi^ cells and macrophages as Ly6C^−^ cells, with most macrophages expressing major histocompatibility complex class II (MHC-II; Extended Data Fig. 1a). All macrophages expressed F4/80; this was substantially lower in monocytes (Extended Data Fig. 1b). In mice, monocyte numbers peaked during proestrus. During the following cycle stage (estrus), Ly6C^hi^ cells acquired expression of MHC-II and F4/80 compared with proestrus, suggesting that they were differentiating in situ (Extended Data Fig. 1c). Expression of F4/80 was consistently high in macrophages across the cycle (Extended Data Fig. 1d). There was a positive correlation between F4/80 and MHC-II expression by monocytes (Extended Data Fig. 1e).

It is more difficult to distinguish monocytes from macrophages in human tissues because both express human leukocyte antigen (HLA)-DR and neither expresses Ly6C or F4/80. However, CCR2 is highly expressed on blood monocytes and is essential for egress from bone marrow (BM) and recruitment into peripheral tissues^19–21^; thus we used C–C motif chemokine receptor 2 (CCR2) to distinguish human monocytes (Extended Data Fig. 1f). As in mice, there were dramatic fluctuations in monocyte numbers across the menstrual cycle in humans, with a stark increase in endometrial monocytes during the early secretory phase (Fig. 1b), which precedes the window of implantation in the mid secretory phase. The CCR2^+^ cells had a characteristic monocyte-like appearance, as indicated by low side scatter, whereas CCR2^−^ cells had the more granular appearance typical of macrophages (Extended Data Fig. 1g). The expression of cleaved caspase 3 (ClC3) in human HLA-DR^+^ cells (predominantly monocytes or macrophages) during the menstrual cycle suggests active apoptosis in monocytes or macrophages (Fig. 1c), implying a requirement for precursor cell recruitment (early secretory phase).

To define macrophage replenishment, CD45.1 mice were irradiated with their abdomens shielded to deplete BM precursors, then reconstituted with BM cells from CD45.2 (wild-type (WT) or *Ccr2*^−/−^) congenic animals. Flow cytometric analysis showed that BM-derived cells recruited from the circulation replenished the uterine macrophage pool. Although uterine macrophage turnover was initially slower than intestinal macrophages, a population with one of the highest reported replenishment rates of all tissue macrophages^22^, by 14 weeks post-irradiation uterine replenishment was equivalent to that in the intestine (Fig. 1d). This was dependent on CCR2, supporting a requirement for classic monocytes (Fig. 1e).

### Monocytes give rise to distinct populations of mature macrophages with proinflammatory or pro-repair properties

**Fig. 2:**
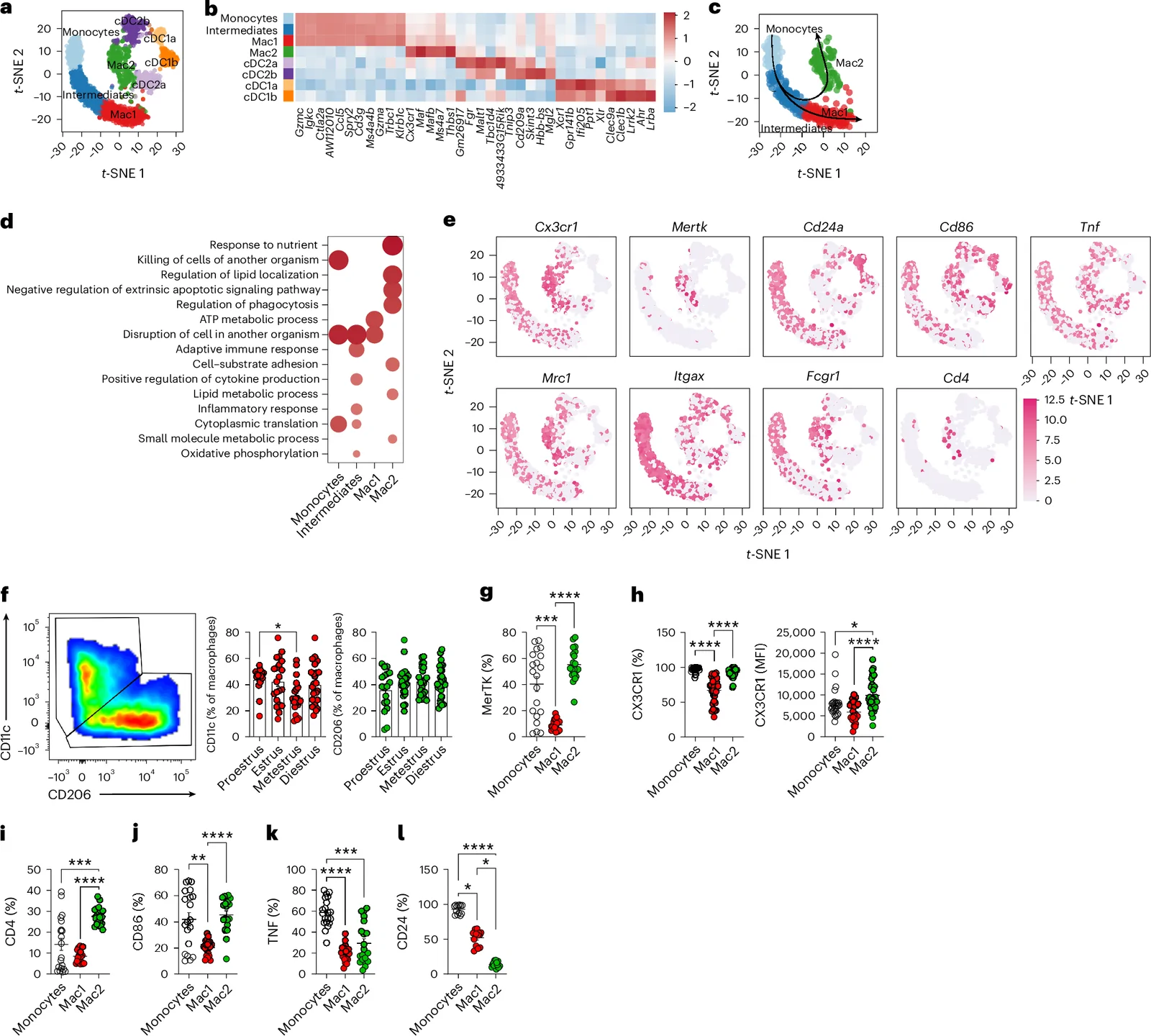
scRNA-seq analysis in pooled mouse uteri. **a**, The *t*-distributed stochastic neighbor embedding (*t*-SNE) of sorted and pooled murine uterine myeloid cells (CD45^+^ live SigF^−^Ly6G^−^CD3^−^CD19^−^CD20^−^CD56^−^ cells expressing CD11b and/or MHC-II) from uteri across all cycle stages. The points represent individual cells and color cluster assignment. **b**, Heatmap of the relative average expression of the top cluster-defining genes selected by average log_2_(fold-change) relative to other clusters. **c**, The *t*-SNE of macrophage subsets with pseudotime curve projections. **d**, Gene set enrichment analysis (GSEA) using gene ontology (GO) biological process (BP) terms for monocyte and macrophage subsets. Point size and color represent the enrichment score. All plotted points have an adjusted *P* (*P*_adj_) < 0.05. **e**, The *t*-SNE plots with point color representing relative gene expression. **f**, Representative flow cytometry plot of gating for murine macrophage subsets based on scRNA-seq analysis with CD11c identifying Mac1 macrophages and CD206 identifying Mac2 macrophages and summary graphs demonstrating expression of CD11c and CD206 on differentiated macrophages at different estrus cycle stages assessed by flow cytometry. A one-way ANOVA was applied in both cases. The proportion of CD11c^+^ macrophages (within CD45^+^ cells) was as follows: proestrus, *n* = 11; estrus, *n* = 14; metestrus, *n* = 18; and diestrus, *n* = 16. The proportion of CD206^+^ macrophages (within CD45^+^ cells) was: proestrus, *n* = 13; estrus, *n* = 16; metestrus, *n* = 16; and diestrus, *n* = 21. **g**, Flow cytometric analysis showing proportions of total monocytes, Mac1 macrophages and Mac2 macrophages expressing MERTK (Kruskal–Wallis test, *n* = 20). **h**, CX_3_CR1. Left: percentage of subset; right: MFI; Kruskal–Wallis tests were used. **i**, CD4. A Kruskal–Wallis test was used, *n* = 20. **j**, CD86. A Kruskal–Wallis test was used, *n* = 20. **k**, TNF (intracellular protein). A Kruskal–Wallis test was used, *n* = 20. **l**, CD24. A Kruskal–Wallis test was used, *n* = 13. For all flow cytometry data, at least two independent experiments were carried out. For one-way ANOVA and Kruskal–Wallis tests, *P*_adj_ values were reported from Tukey’s and Dunn’s post hoc tests, respectively. ^*^P < 0.05, ^**^P < 0.01, ^***^P < 0.001, ^****^P < 0.0001. In all cases, the dots represent biological replicates. Data are presented as the mean ± s.e.m. in **f** and as the median ± IQR in **g**, **h**, **i**, **j**, **k** and **l**. cDC, conventional dendritic cell. Source data

To comprehensively profile the uterine myeloid compartment, we performed single-cell RNA sequencing (scRNA-seq) analyses of FACS-sorted myeloid cells from multiple mouse uteri to represent all stages of the estrus cycle. This pre-enrichment excluded the extensive stromal fraction that has led to underrepresentation of monocytes and dendritic cells in other uterine single-cell studies^15,23–29^. Pre-enriching for myeloid cells before scRNA-seq showed that uterine monocytes and macrophages could be clearly distinguished from dendritic cells (Fig. 2a) based on their expression of the macrophage-specific transcription factor, *Mafb* macrophage-activating factor (*Maf*) and the monocyte marker, *Ms4a7*^30^ (Fig. 2b).

**Extended Data Fig. 2:**
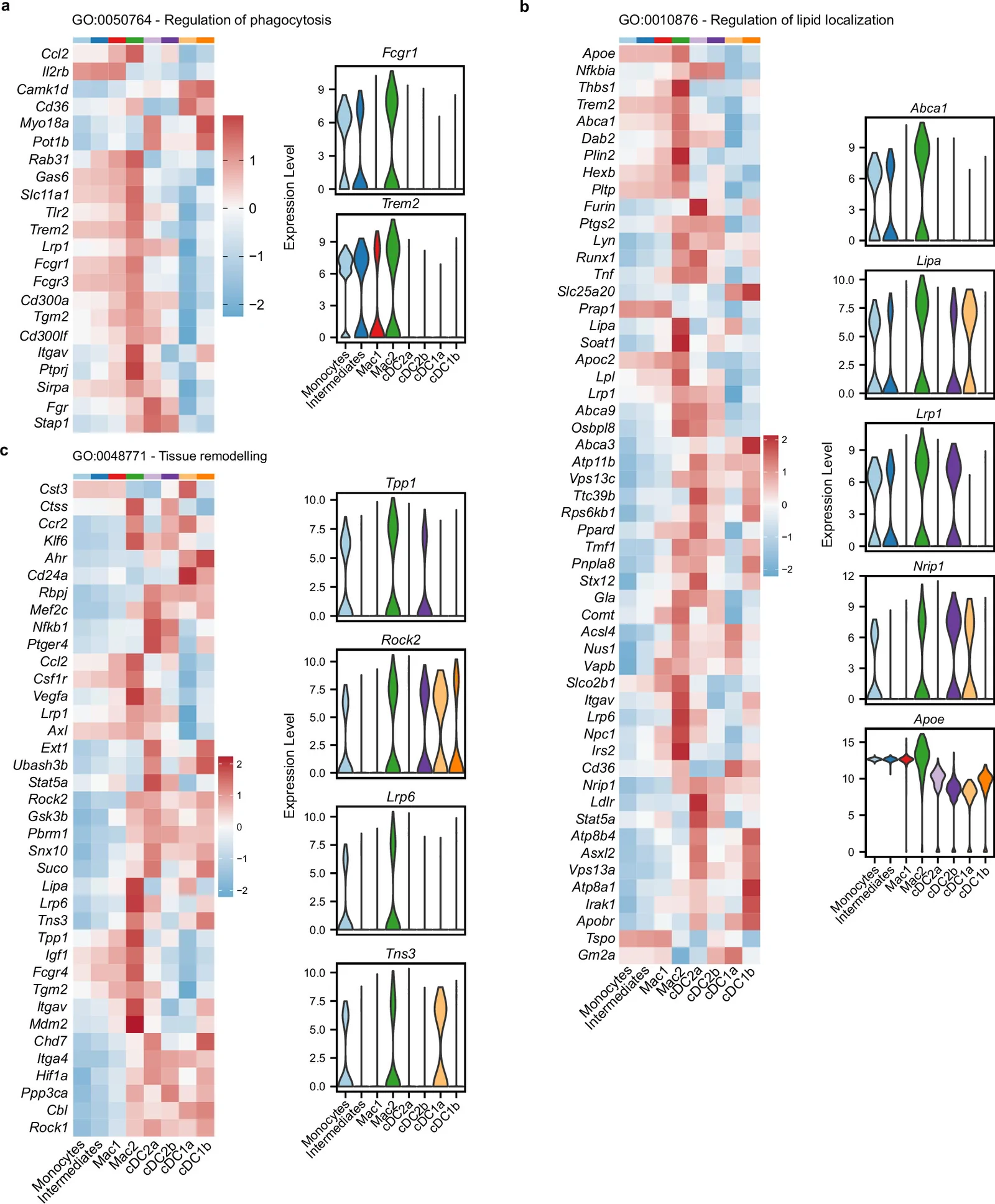
Summary data for selected gene ontology terms enriched in mac2 macrophages in mouse single cell RNAseq analysis of myeloid cells. Heatmaps representing relative cell cluster average expression of genes in the **a**) ‘regulation of phagocytosis’, **b**) ‘regulation of lipid localization’ and **c**) ‘tissue remodelling’ gene ontology (GO) biological processes terms with violin plots showing the distribution of expression of selected genes within cell clusters. Within heatmaps only genes in a given term that were statistically significantly enriched in at least one cell cluster are plotted (adjusted p value < 0.01 & average log2 fold change > 1). Source data

**Extended Data Fig. 3:**
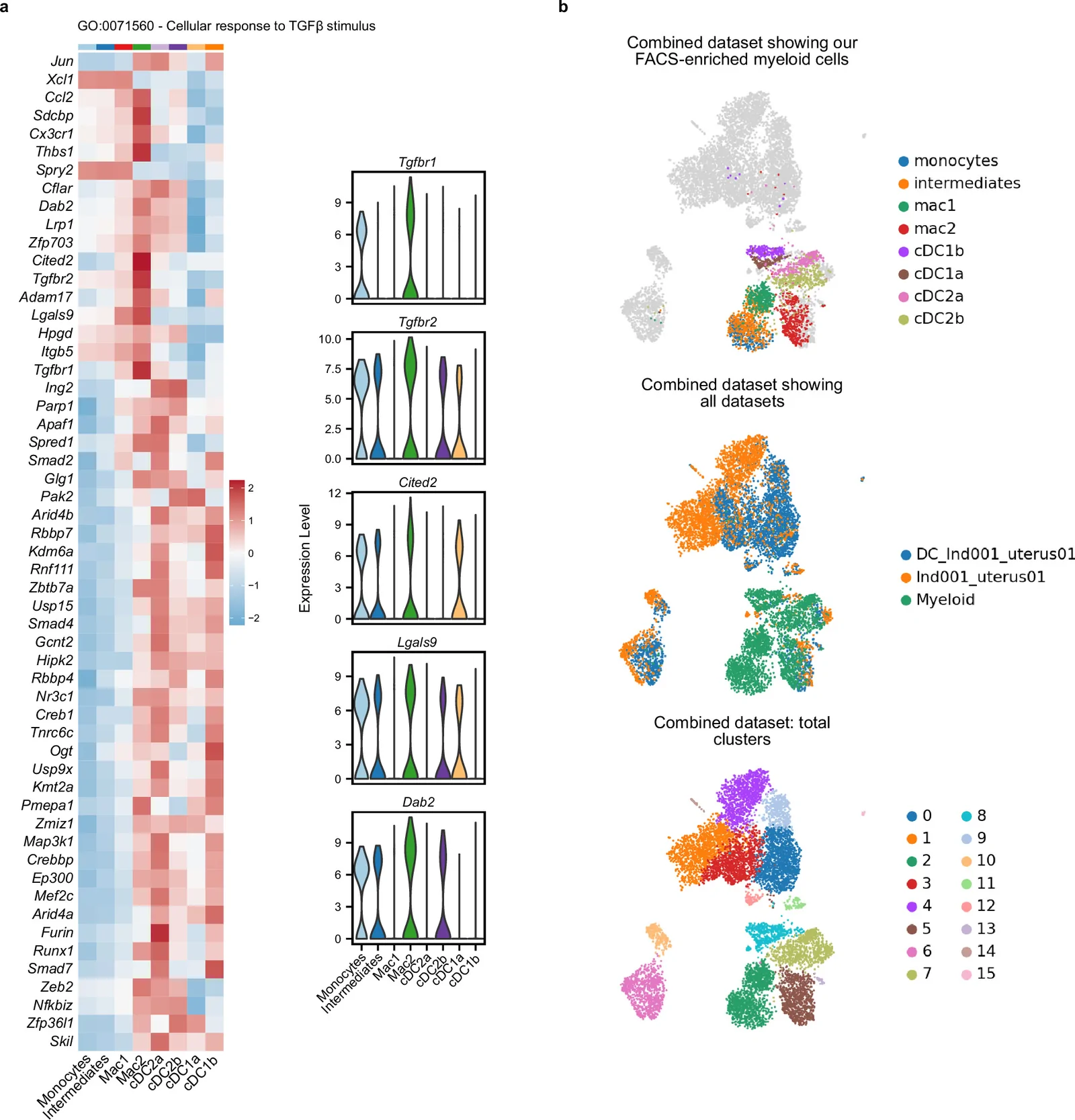
Mouse single cell RNAseq data and integration with other datasets. **a**) Heatmap representing relative cell cluster average expression of genes in the ‘cellular response to TGFβ stimulus’ gene ontology (GO) biological processes term with violin plots showing the distribution of expression of selected genes within cell clusters. Only genes that were statistically significantly enriched in at least one cell cluster are plotted (adjusted p value < 0.01 & average log2 fold change > 1). **b**) Batch Balanced KNN was used to integrate our FACS enriched myeloid single cell data with literature data. Top panel: cell types in our FACS-enriched myeloid dataset are coloured and literature data is grey. Middle panel: FACS-enriched myeloid dataset is shown in green, literature data is shown in orange and blue. Bottom panel: Total clusters from combined datasets. Source data

Monocytes clustered with an intermediary population of cells and a subset that we termed Mac1 macrophages (Fig. 2a), with pathway analysis demonstrating inflammatory properties including cytotoxicity in monocytes. Inflammatory response genes were expressed in the intermediate cells (Fig. 2d), which appear to be the Ly6C^hi^MHC-II^+^ monocytes that differentiate into macrophages and accumulate during estrus (Extended Data Fig. 1a). Both intermediate cells and Mac1 macrophages expressed genes involved in oxidative phosphorylation (Fig. 2d), a process associated with macrophage activation in other mucosal tissues^31^. Accordingly, Mac1 macrophages also expressed genes involved in positive regulation of inflammatory responses and in metabolic processes involved in rapid ATP generation (Fig. 2d). Slingshot trajectory analysis revealed uterine monocytes as a likely common precursor of both Mac1 macrophages and a second subset of macrophages that we termed Mac2 macrophages (Fig. 2c). In contrast to Mac1 macrophages, Mac2 macrophages expressed genes involved in phagocytosis, apoptotic cell clearance, regulation of lipid localization (Fig. 2d and Extended Data Fig. 2a,b), tissue remodeling (Extended Data Fig. 2c) and cellular responses to transforming growth factor (TGF)-β (including *Tgfbr1* and *Tgfbr2*; Extended Data Fig. 3a). When we applied the Batch Balanced, *k*-nearest neighbors method^32^ to integrate our FACS-enriched myeloid cell data with published single-cell studies of the murine estrus cycle^23^ (Extended Data Fig. 3b), we found that our Mac2 macrophages and dendritic cell subsets could be detected in these previous studies. However, monocytes, intermediates and Mac1 macrophages were not represented, consistent with them having been diluted out in datasets lacking myeloid enrichment.

As we found by flow cytometry, all monocyte and macrophage clusters expressed *Fcgr1a* encoding CD64 (Fig. 2e), allowing them to be distinguished from dendritic cells^33^. Surface markers that were predicted to identify monocytes included Ly6C, whereas Mac1 macrophages were CX_3_CR1^lo^, MERTK^−^, CD206^−^ (encoded by *Mrc1*) and CD24^+^ (encoded by *Cd24a*), CD11c^+^ (encoded by *Itgax*) and Mac2 macrophages were CX_3_CR1^hi^, MERTK^+^, CD206^+^, CD24^lo^, CD11c^−^ and CD4^+^ (Fig. 2e). Of these, the clearest markers for distinguishing Mac1 and Mac2 macrophages were predicted to be CD11c and CD206, respectively, and this was confirmed by flow cytometry gating (Fig. 2f). It is interesting that there was little change in the relative proportions of Mac1 and Mac2 macrophages across the estrus cycle other than a slight reduction in Mac1 macrophages at metestrus (Fig. 2f).

**Extended Data Fig. 4:**
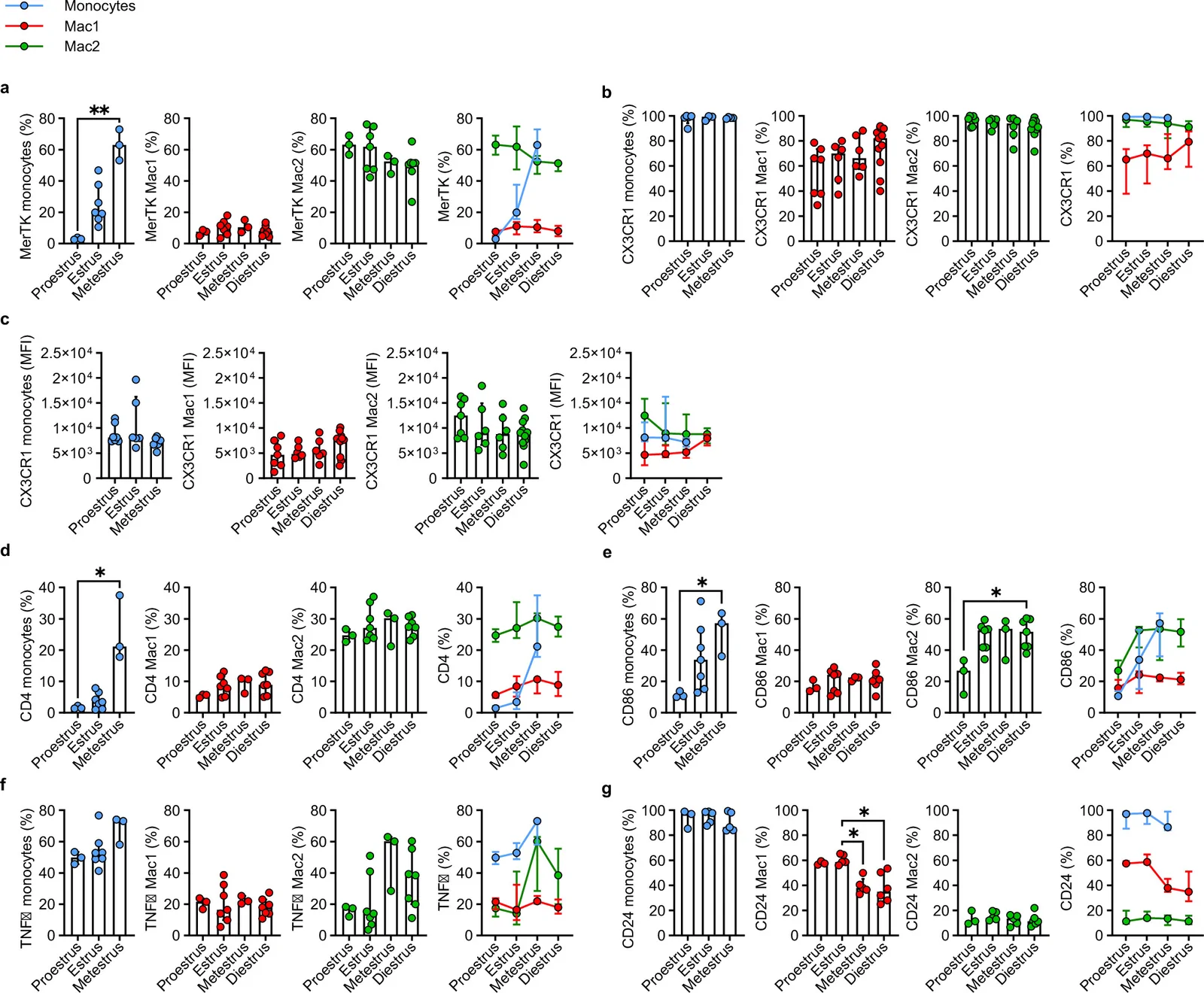
Flow cytometry validation of markers identified by single cell RNAseq. **a**) Summary graphs demonstrating proportions of monocytes, Mac1 and Mac2 macrophages expressing MERTK across different stages of the estrous cycle (proestrus: n = 3, estrus: n = 7, metestrus: n = 3, diestrus (Mac1 and Mac2 macrophages only): n = 7). Monocytes: Kruskal-Wallis test was carried out. Combined graph: Two-way ANOVA was applied (main effects only model) with significant differences in MERTK expression between cell types (**p < 0.01 for monocytes versus Mac1, ****p < 0.0001 for monocytes versus Mac2 and Mac1 versus Mac2 macrophages) **b**) Summary graphs demonstrating proportions of monocytes (proestrus: n = 5, estrus: n = 3, metestrus: n = 4) Mac1 (proestrus: n = 7, estrus: n = 6, metestrus: n = 6, diestrus: n = 11,) and Mac2 (proestrus: n = 7, estrus: n = 6, metestrus: n = 6, diestrus: n = 11) macrophages expressing CX_3_CR1 expression across different stages of the estrous cycle. Combined graph: Two-way ANOVA was applied (main effects only model) with significant differences in CX_3_CR1 expression between cell types (****p < 0.0001 for monocytes versus Mac1 macrophages, and Mac1 versus Mac2 macrophages). **c**) Summary graphs demonstrating levels of CX_3_CR1 expression per cell as indicated by mean fluorescence intensity, by monocytes (proestrus: n = 5, estrus: n = 3, metestrus: n = 4) Mac1 (proestrus: n = 7, estrus: n = 6, metestrus: n = 6, diestrus: n = 11) and Mac2 (proestrus: n = 7, estrus: n = 6, metestrus: n = 6, diestrus: n = 11) macrophages across different stages of the estrous cycle. Combined graph: Two-way ANOVA was applied (main effects only model) with significant differences in CX_3_CR1 MFI between cell types (*p < 0.05 for monocytes versus Mac1 macrophages, and ****p < 0.0001 for Mac1 versus Mac2 macrophages). **d**) Summary graphs demonstrating proportions of monocytes, Mac1 and Mac2 macrophages expressing CD4 across different stages of the estrous cycle (proestrus: n = 3, estrus: n = 7, metestrus: n = 3, diestrus (Mac1 and Mac2 only): n = 7). Monocytes: Kruskal-Wallis test was carried out. Combined graph: Two-way ANOVA was applied (main effects only model) with significant differences in CD4 expression between cell types (****p < 0.0001 for monocytes versus Mac2 macrophages, and Mac1 versus Mac2 macrophages). **e**) Summary graphs demonstrating proportions of monocytes, Mac1 and Mac2 macrophages expressing CD86 across different stages of the estrous cycle (proestrus: n = 3, estrus: n = 7, metestrus: n = 3, diestrus (Mac1 and Mac2 only): n = 7). Monocytes and Mac2 macrophages: Kruskal-Wallis tests were carried out. Combined graph: Two-way ANOVA was applied (main effects only model) with significant differences in CD86 expression between cell types (**p < 0.01 for monocytes versus Mac1 macrophages, ****p < 0.0001 for Mac1 versus Mac2 macrophages). **f**) Summary graphs demonstrating proportions of monocytes, Mac1 and Mac2 macrophages producing TNFα following 3 h incubation with protein transport inhibitor, across different stages of the estrous cycle (proestrus: n = 3, estrus: n = 7, metestrus: n = 3, diestrus (Mac1 and Mac2 only): n = 7). Combined graph: Two-way ANOVA (main effects only model) was applied with significant differences in TNFα production between cell types (****p < 0.0001 for monocytes versus Mac1 macrophages, and monocytes versus Mac2 macrophages). **g**) Summary graphs demonstrating proportions of monocytes, Mac1 and Mac2 macrophages expressing CD24 across different stages of the estrous cycle (proestrus: n = 3, estrus: n = 5, metestrus: n = 5, diestrus (Mac1 and Mac2 only): n = 6). Mac1 macrophages: Kruskal-Wallis test was carried out. Combined graph: Two-way ANOVA was applied with significant differences in CD24 expression between cell types (****p < 0.0001 in all cases). In all cases, at least two independent experiments were carried out. For one and two-way ANOVA and Kruskal-Wallis tests adjusted p values are reported from Tukey and Dunn’s *post hoc* tests respectively. *p < 0.05, **p < 0.01, ***p < 0.001, ****p < 0.0001. Data are presented as median±IQR in **a**, **b**, **c**, **d**, **e**, **f**, **g**. Source data

At protein level, MERTK, a molecule involved in apoptotic cell clearance, was preferentially expressed on Mac2 macrophages, fitting a tissue-repair profile (Fig. 2g). Expression was consistent across the cycle on both macrophage subsets but fluctuated extensively on monocytes (Extended Data Fig. 4a). CX_3_CR1 was present on nearly all monocytes and macrophages, but mean fluorescence intensity (MFI) showed that it was most highly expressed by Mac2 macrophages and monocytes (Fig. 2h); this was consistent across the cycle (Extended Data Fig. 4b,c). The CD gene (*Cd4*, Fig. 2e) and protein (Fig. 2i) were expressed mainly by Mac2 macrophages. CD4 was expressed by macrophage subsets consistently across the estrus cycle, whereas expression on monocytes fluctuated extensively depending on the cycle stage (Extended Data Fig. 4d). The activation marker CD86 was expressed on a higher proportion of Mac2 macrophages (Fig. 2j), but there was a drop-off in expression by both monocytes and Mac2 macrophages during proestrus (Extended Data Fig. 4e). Tumor necrosis factor (TNF) was potently produced by unstimulated monocytes to a greater degree than either macrophage subset (Fig. 2k). There were no substantial differences between cycle stages for TNF production, for any cell type (Extended Data Fig. 4f).

CD24 protein was highest in monocytes, but expression declined through differentiation into Mac1 macrophages and, overall, was substantially lower on Mac2 macrophages (Fig. 2l). Expression on Mac1 macrophages fluctuated across estrus cycle stages, peaking during or shortly after periods of most active monocyte infiltration (proestrus and estrus; Extended Data Fig. 4g). This is consistent with a role for CD24 in facilitating adhesion to endothelial cells^34^ and trajectory analysis indicating that this population was recently differentiated from monocytes (Fig. 2c).

### Different rates of replenishment of uterine macrophage subsets from the bloodstream

**Fig. 3:**
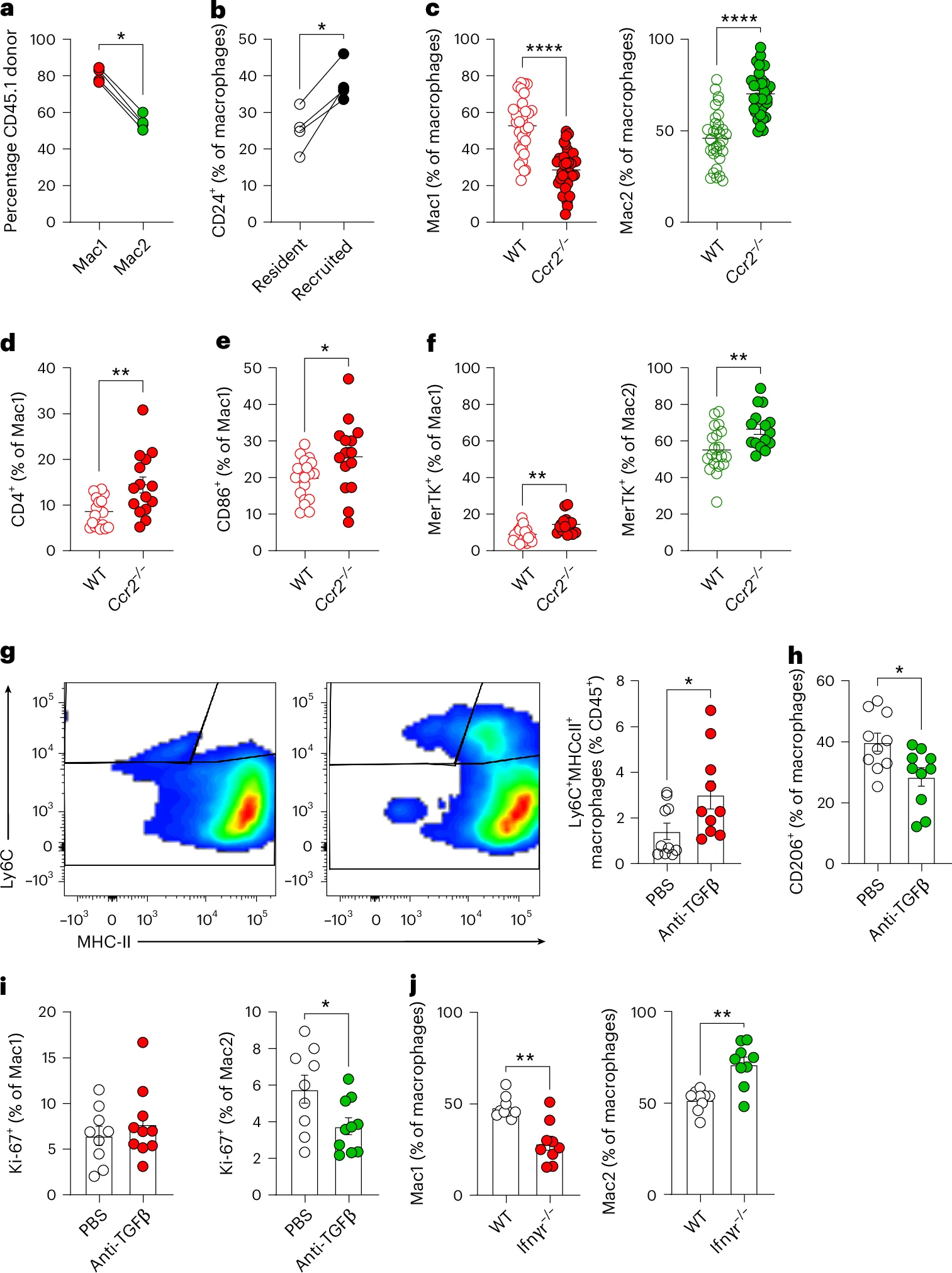
Differentiation of mouse Mac1 and Mac2 macrophages. **a**, Summary graph showing proportions of uterine murine Mac1 and Mac2 macrophage subsets comprising donor (CD45.1) cells recruited from the bloodstream 12 weeks post-irradiation. The results were normalized to chimerism in blood monocytes. A two-tailed Mann–Whitney *U*-test was applied (*n* = 4). **b**, Proportions of total recruited versus resident macrophages expressing CD24. A two-tailed Mann–Whitney *U*-test was applied (*n* = 4). **c**, Proportions of Mac1 and Mac2 macrophage subsets within the total macrophage pool in WT (*n* = 32) and *Ccr2*^*−/−*^ (*n* = 36) mice. An unpaired, two-tailed Student’s *t*-test was applied in both cases: ^****^P < 0.0001. **d**, Proportions of Mac1 macrophages expressing CD4 in WT (*n* = 20) and *Ccr2*^*−/−*^ (*n* = 15) mice. A two-tailed Mann–Whitney *U*-test was applied. **e**, Proportions of Mac1 macrophages expressing CD86 in WT (*n* = 20) and *Ccr2*^*−/−*^ (*n* = 15) mice. A two-tailed, unpaired Student’s *t*-test was applied: ^*^P < 0.05. **f**, Proportions of Mac1 and Mac2 macrophages expressing MERTK in WT (*n* = 20) and *Ccr2*^*−/−*^ (*n* = 15) mice. A two-tailed Mann–Whitney *U*-test was applied in both cases. **g**, Example flow cytometry gating identifying monocytes and macrophages (pre-gated on CD45^+^ live Ly6G^−^SigF^−^CD64^+^CD11b^+^) and summary graphs showing proportions of Ly6C^+^MHC-II^+^ inflammatory intermediates in mice administered phosphate-buffered saline (PBS; left) (*n* = 10) or anti-TGFβ (right) (*n* = 10) intraperitoneally (i.p.). A two-tailed Mann–Whitney *U*-test was applied. Mice were all culled in the diestrus stage of the cycle. **h**, Proportions of Mac2 macrophages in mice administered PBS (*n* = 10) or anti-TGFβ (*n* = 10) i.p. A two-tailed, unpaired Student’s *t*-test was applied. **i**, Proportions of Mac1 and Mac2 macrophages expressing Ki-67 in mice administered PBS (*n* = 10) or anti-TGFβ (*n* = 10) i.p. A two-tailed, unpaired Student’s *t*-test was applied. **j**, Proportions of Mac1 and Mac2 macrophages in WT (*n* = 9) and *Ifnγr*^*−/−*^ (*n* = 9) mice. A two*-*tailed Mann–Whitney *U*-test was applied in both cases. All data represent at least two independent experiments. ^*^P < 0.05, ^**^P < 0.01, ^****^P < 0.0001. Data are presented as the mean ± s.e.m. in **c**, **e**, **h** and **i** and as the median ± IQR in **d**, **f**, **g** and **j**. Source data

This conclusion was supported by shielded BM chimera experiments, which showed that, by 12 weeks post-irradiation, a greater proportion of Mac1 macrophages had been replenished from circulating monocytes compared with Mac2 macrophages (Fig. 3a). In addition, a greater proportion of macrophages derived from recruited donor cells expressed CD24 compared with their tissue-resident counterparts (Fig. 3b).

**Extended Data Fig. 5:**
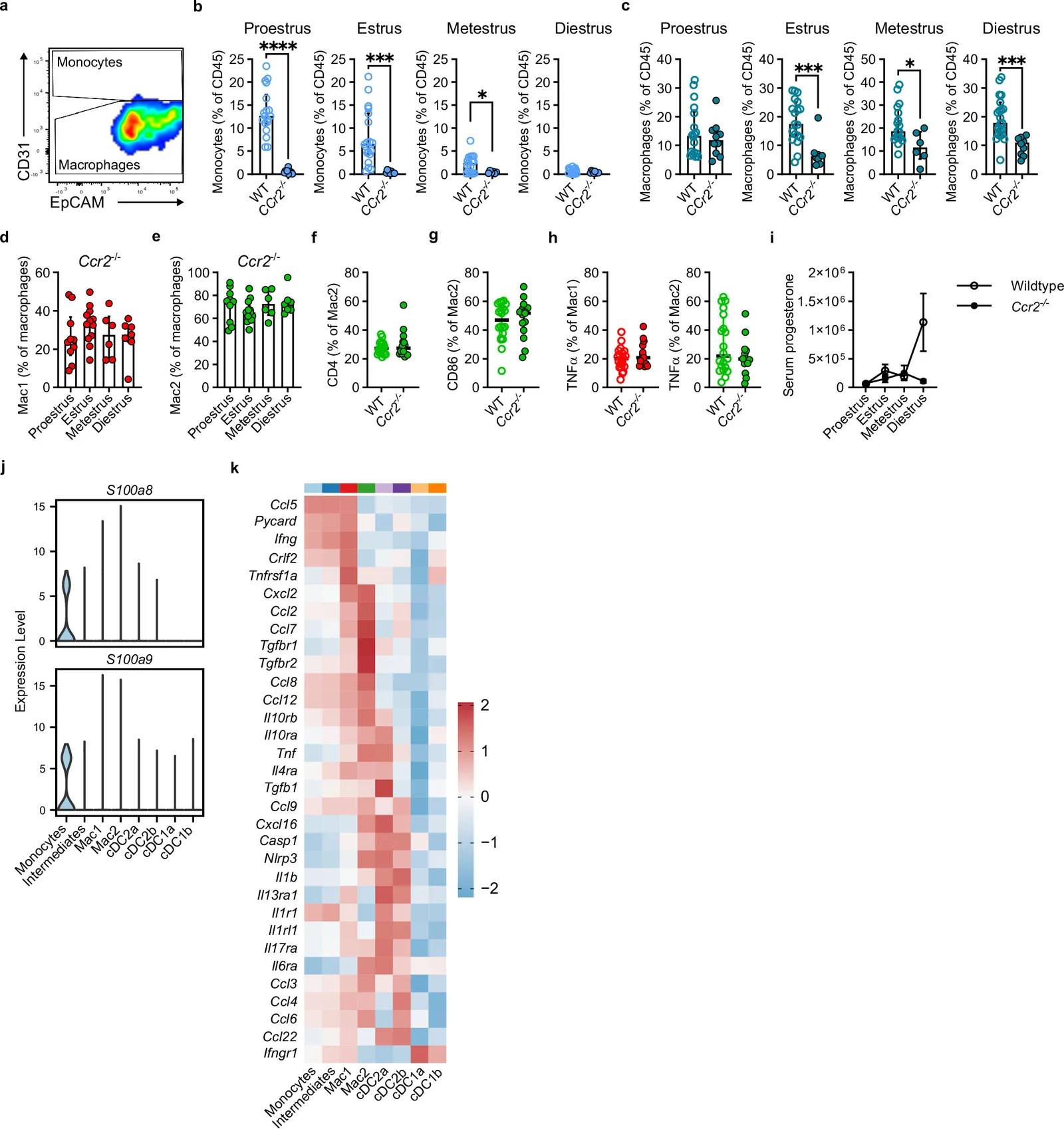
Monocytes and macrophages in *Ccr2*^*−/−*^ mice and single cell RNAseq data. **a**) Flow cytometry plot demonstrating lack of uterine monocytes in *Ccr2*^*−/−*^ mice (pre-gated on CD45^+^live SigF^−^Ly6G^−^CD11b^+^CD64^+^ cells). **b**) Summary data demonstrating proportions of monocytes (within CD45^+^ cells) in WT and *Ccr2*^*−/−*^ mice during different estrous cycle stages (proestrus: WT n = 18, *Ccr2*^*−/−*^ n = 10; estrus: WT n = 19, *Ccr2*^*−/−*^ n = 9; metestrus: WT n = 17, *Ccr2*^*−/−*^ n = 6; diestrus: WT n = 23, *Ccr2*^*−/−*^ n = 10). Two-tailed unpaired t-test was applied in all cases. **c**) Summary data demonstrating proportions of macrophages (within CD45^+^ cells) in WT and *Ccr2*^*−/−*^ mice during different estrous cycle stages (proestrus: WT n = 18, *Ccr2*^*−/−*^ n = 10; estrus: WT n = 19, *Ccr2*^*−/−*^ n = 9; metestrus: WT n = 17, *Ccr2*^*−/−*^ n = 6; diestrus: WT n = 23, *Ccr2*^*−/−*^ n = 10). Two-tailed unpaired t-test was applied in all cases. **d**) Summary data demonstrating proportions of uterine Mac1 macrophages (within total macrophages) at different stages of the estrous cycle in *Ccr2*^*−/−*^ mice (proestrus: n = 10, estrus: n = 13, metestrus: n = 6, diestrus: n = 7). **e**) Summary data demonstrating proportions of uterine Mac2 macrophages (within total macrophages) at different stages of the estrous cycle in *Ccr2*^*−/−*^ mice (proestrus: n = 10, estrus: n = 13, metestrus: n = 6, diestrus: n = 7). **f**) Summary data demonstrating proportions of Mac2 macrophages expressing CD4 in WT (n = 19) and *Ccr2*^*−/−*^ (n = 15) mice; all cycle stages represented. **g**) Summary data demonstrating proportions of Mac2 macrophages expressing CD86 in WT (n = 19) and *Ccr2*^*−/−*^ (n = 15) mice; all cycle stages represented. **h**) Summary data demonstrating proportions of Mac1 and Mac2 macrophages producing TNFα in WT (n = 19) and *Ccr2*^*−/−*^ (n = 15) mice; all cycle stages represented. **i**) Serum progesterone levels as measured by mass spectrometry in WT (proestrus: n = 3, estrus: n = 7, metestrus: n = 3, diestrus: n = 8) and *Ccr2*^*−/−*^ mice (proestrus: n = 3, estrus: n = 6, metestrus: n = 3, diestrus: n = 3). Two-way ANOVA was carried out with Tukey’s multiple comparisons test, *p < 0.05 (for serum progesterone levels during diestrus only). **j**) Violin plots showing the distribution of expression of *S100a8* and *S100a9* in murine uterine myeloid cells from single cell RNAseq data. **k**) Heatmap representing relative cell cluster average expression of genes related to response to and production of cytokines and chemokines, and inflammatory signalling. In all cases, at least two independent experiments were carried out. For two-way ANOVA tests adjusted p values are reported from Tukey *post hoc* tests respectively. *p < 0.05, **p < 0.01, ***p < 0.001, ****p < 0.0001. Data are presented as mean±SEM in **b**, **c**, **d**, **e**, **f**, **g**, **h**. Source data

Uterine monocytes were absent from *Ccr2*^*−/−*^ mice at all cycle stages (Extended Data Fig. 5a,b) and there was a loss of total macrophage numbers at most cycle stages (Extended Data Fig. 5c), consistent with the requirement for constant replenishment from monocytes. This reduction was primarily due to loss of CD11c^+^ Mac1 macrophages, resulting in a macrophage compartment dominated by Mac2 macrophages (Fig. 3c), but their relative proportions remained constant throughout the estrus cycle in *Ccr2*^*−/−*^ mice (Extended Data Figs. 5d,e). It is interesting that residual Mac1 macrophages in *Ccr2*^*−/−*^ mice showed enhanced expression of Mac2-‘like’ markers such as CD4 (Fig. 3d), CD86 (Fig. 3e) and MERTK (Fig. 3f). MERTK was also expressed by a greater proportion of Mac2 macrophages in *Ccr2*^*−/−*^ mice (Fig. 3f). CD4 (Extended Data Fig. 5f) and CD86 (Extended Data Fig. 5g) remained unchanged on Mac2 macrophages. TNF production in both macrophage subsets in *Ccr2*^*−/−*^ mice was comparable to WT levels (Extended Data Fig. 5h). As macrophages can influence ovarian function^35–37^, we assessed whether *Ccr2*^*−/−*^ mice have alterations in hormone levels. Progesterone levels were comparable between WT and *Ccr2*^*−/−*^ mice at all cycle stages except diestrus (Extended Data Fig. 5i).

### Differentiation of macrophage subsets in the uterus is dependent on TGFβ and IFNγ

scRNA-seq analysis of murine uterine myeloid cells confirmed an inflammatory signature in monocytes with expression of *S100a8* and *S100a9* (Extended Data Fig. 5j) and other inflammatory molecules with expression that was sustained in Mac1 macrophages, including high expression of *Ccl5, Pycard* and *Ifng* (Extended Data Fig. 5k). As well as genes involved in tissue repair and remodeling, Mac2 macrophages expressed inflammatory chemokines including *Ccl2*, suggesting involvement in monocyte recruitment to the uterus (Extended Data Fig. 5k). Nevertheless, Mac2 macrophages selectively expressed much higher levels of receptors for anti-inflammatory and type 2 cytokines including *Il10ra* and *Il10rb*, as well as genes involved in the response to TGFβ, including *Tgfbr1* and *Tgfbr2* (Extended Data Fig. 5k). To investigate the role of TGFβ in differentiation of Mac2 macrophages, we used a TGFβ-blocking antibody to inhibit the effects of TGFβ in vivo. This caused the accumulation of inflammatory intermediate monocytes or macrophages co-expressing Ly6C with MHC-II (Fig. 3g), suggesting that TGFβ is required for macrophage differentiation in the uterus. Furthermore, inhibition of TGFβ selectively reduced the abundance of Mac2 macrophages (Fig. 3h) and diminished their expression of the cell cycle marker Ki-67 indicating proliferation (Fig. 3i). These data suggest that TGFβ is required at least in part for the self-renewal and differentiation of Mac2 macrophages in the uterus.

In contrast, Mac1 macrophages selectively expressed *Ifngr1* (Extended Data Fig. 5k) and, indeed, *Ifngr1-*deficient mice had a reduced proportion of Mac1 macrophages and a corresponding increase in Mac2 macrophages (Fig. 3j). Thus, TGFβ and interferon (IFN)γ play distinct roles in shaping differentiation of Mac2 and Mac1 macrophages in the uterus, respectively.

### Uterine monocyte and macrophage populations are conserved in humans

**Fig. 4:**
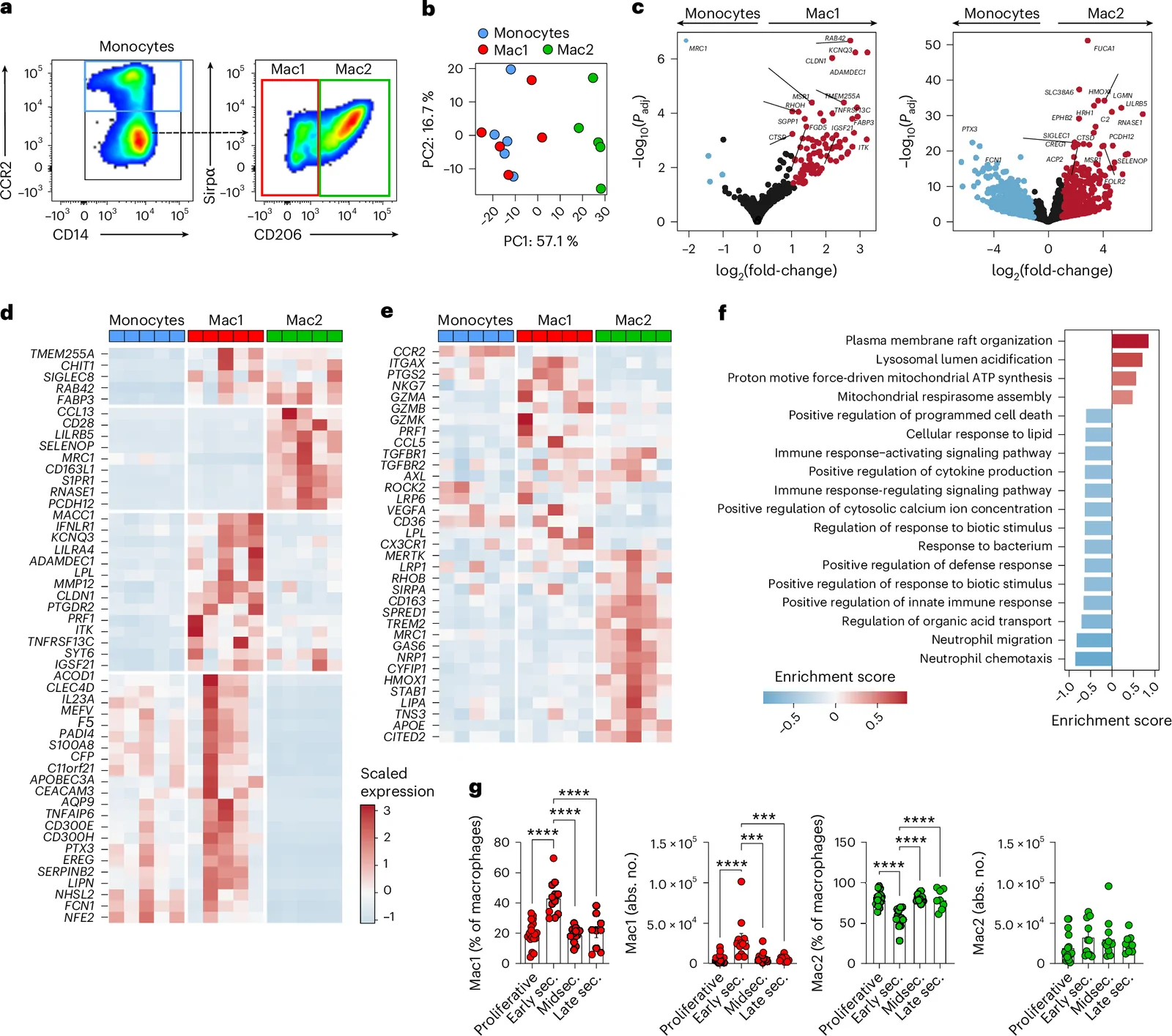
RNA-seq analysis of human endometrial monocytes and macrophages. **a**, FACS-gating strategy with all cells pre-gated on CD45^+^ live CD3^−^CD19^−^CD66b^−^Sig8^−^HLA-DR^+^CD64^+^ cells and monocytes identified as CCR2^+^CD14^+^ cells with differentiated macrophages identified as CCR2^−^CD14^+^ cells. Differentiated macrophages were then split into CD206^−^ (Mac1 macrophage) and CD206^+^ (Mac2 macrophage) subsets. CCR2^+^CD14^+^ monocytes and Mac1 or Mac2 macrophage subsets were isolated for bulk RNA-seq analysis. **b**, Ordination plot of principal component analysis of bulk RNA-seq data using the 250 most variable genes. The first two principal components are plotted. **c**, Volcano plots representing differential gene expression between either sorted Mac1 or Mac2 macrophages with sorted monocytes. Black points indicate genes that were not significantly differentially expressed. Genes were considered significantly differentially expressed when *P*_adj _< 0.05 and the absolute log_2_(fold-change) > 1. **d**, Heatmap representing the relative expression of the top 50 differentially expressed genes between cell subsets. The top genes were selected by identifying the 200 genes with the lowest *P*_adj _value, then selecting the 50 with the greatest log_2_(fold-change). **e**, Heatmap representing the relative expression of genes identified as cluster defining in scRNA-seq analysis of mouse uterine myeloid populations. **f**, GSEA of GO BP terms comparing differentially expressed genes between Mac1 and Mac2 macrophage subsets. All plotted terms had a *P*_adj_ < 0.05. **g**, Summary graphs demonstrating proportions (of total CD45^+^ cells) and absolute numbers (per mg of tissue) of Mac1 (CD206^−^) macrophages and Mac2 (CD206^+^) macrophages at different menstrual cycle stages. A one-way ANOVA was applied in all cases: ^***^P < 0.001, ^****^P < 0.0001; proliferative, *n* = 20; early secretory, *n* = 12; midsecretory, *n* = 12; and late secretory, *n* = 9. For one-way ANOVA *P*_adj_ values are reported from Tukey’s post hoc test. All data represent at least two independent experiment and are presented as the mean ± s.e.m. in **g**. PC, principal component. Source data

**Extended Data Fig. 6:**
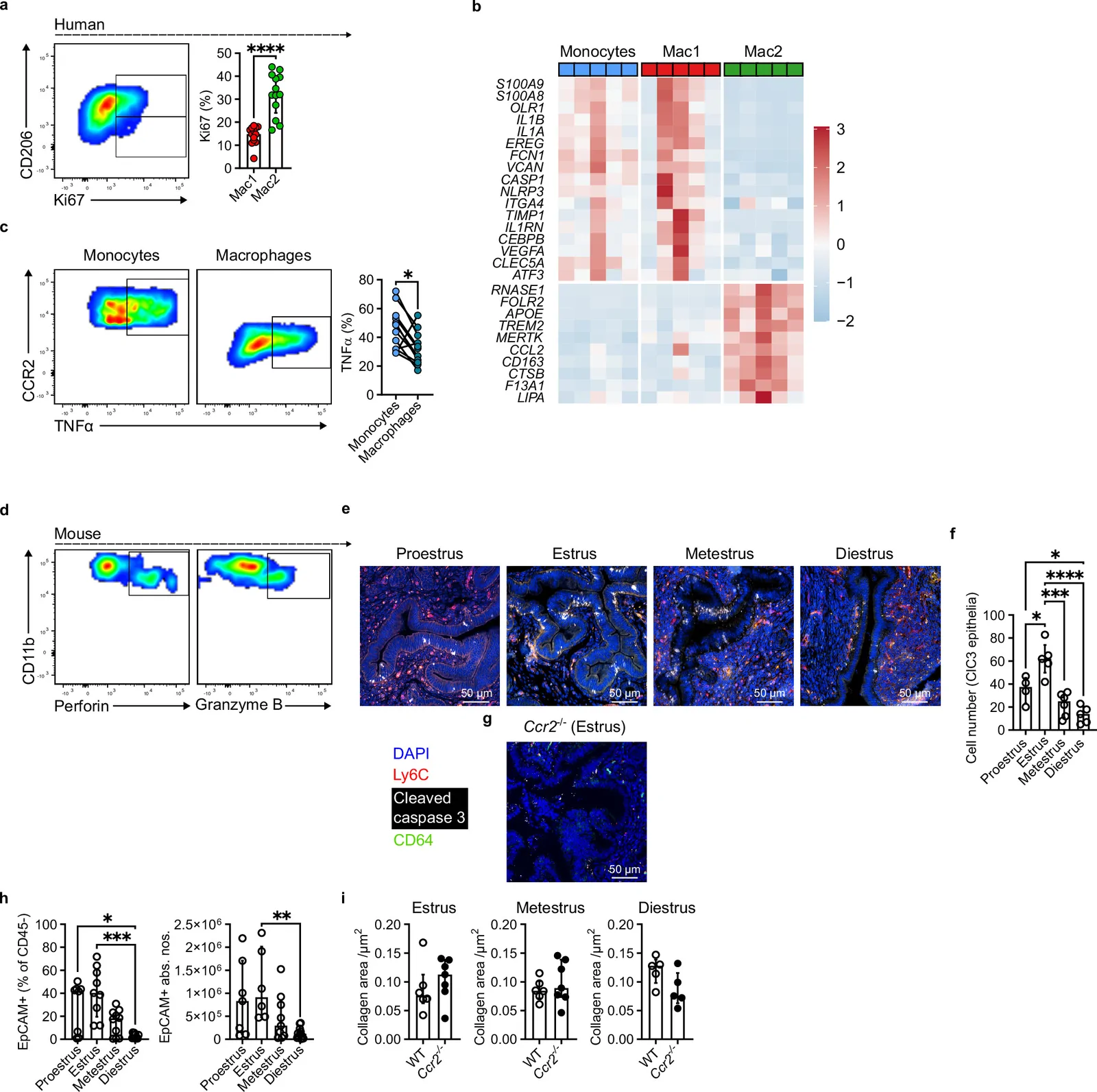
Further characterisation of monocytes and macrophages and epithelial cell apoptosis. **a**) Flow cytometry plot and summary graph showing proportions of human endometrial CD206^−^ (Mac1) macrophages, and CD206^+^ (Mac2 macrophages) expressing Ki67. Two-tailed paired t-test was applied (n = 13). **b**) Heatmap representing the relative expression of genes from bulk RNAseq on FACS-sorted myeloid cell subsets, identifying molecules that have been used to define different macrophage subsets in single cell studies of the human endometrium. **c**) Flow cytometry plots and summary graph showing proportions of human endometrial monocytes (CD45^+^live Sig8^−^CD66b^−^CD3^−^CD19^−^CD56^−^HLA-DR^+^CD64^+^CD14^+^CCR2^+^ cells) and macrophages (CD45^+^ live Sig8^−^CD66b^−^CD3^−^CD19^−^CD56^−^HLA-DR^+^CD64^+^CD14^+^CCR2^−^cells) producing TNFα following 3 h incubation with protein transport inhibitor. Two-tailed paired t-test was applied (n = 12). **d**) Flow cytometry plot showing example gating for perforin and granzyme B protein expression by murine monocytes. **e**) Immunofluorescence microscopy representative images demonstrating Ly6C, CD64 and cleaved caspase 3 staining in cross sections of murine uterine horn during diestrus, proestrus, estrus and metestrus in wildtype mice, and **f**) summary graph demonstrating numbers of cells expressing cleaved caspase 3 at different stages of the estrous cycle (proestrus: n = 4, estrus: n = 5, metestrus: n = 6, diestrus: n = 5). One-way ANOVA was applied. **g**) Immunofluorescence microscopy representative image demonstrating Ly6C, CD64 and cleaved caspase 3 staining in cross sections of murine uterine horn during estrus in a *Ccr2*^*−/−*^ mouse. Images are representative of several independent experiments with similar results. **h**) Proportions (of CD45^−^ cells) and absolute numbers (per uterus) of EpCAM^+^ epithelial cells (pre-gated on live CD45^−^ CD31^−^ cells) throughout different estrous cycle stages (proestrus: n = 8, estrus: n = 9, metestrus: n = 10, diestrus: n = 10). Kruskal Wallis test was applied in both cases. **i**) Summary graphs demonstrating collagen fibres assessed by picrosirius red staining, measured in square micrometers at five regions of interest (ROI) per tissue section/biological replicate (0.5μmx0.5μm in size), in WT and *Ccr2*^*−/−*^ mice during different estrous cycle stages (estrus: WT n = 6, *Ccr2*^*−/−*^ n = 7, metestrus: WT n = 6, *Ccr2*^*−/−*^ n = 7, diestrus: WT n = 5, *Ccr2*^*−/−*^ n = 5). In all cases, at least two independent experiments were carried out. For one way ANOVA and Kruskal-Wallis tests, adjusted p values are reported from Tukey and Dunn’s *post hoc* tests respectively. *p < 0.05, **p < 0.01, ***p < 0.001, ****p < 0.0001. Data are presented as mean±SEM in **a**, **f**. Data are presented as median±IQR in **h**, **i**. Source data

To determine whether phenotypically analogous subsets of monocytes and macrophages could be identified in the human uterus, we pre-gated HLA-DR^+^CD64^+^ cells and identified monocytes as CD14^+^CCR2^+^ (Extended Data Fig. 1f). Putative Mac1 and Mac2 macrophages were then identified as CD14^+^CCR2^−^CD206^−^ and CD14^+^CCR2^−^CD206^+^ cells, respectively (Fig. 4a). Bulk RNA-seq analyses of these FACS-isolated subsets showed that, as in mice, human uterine monocytes and Mac1 macrophages were more closely related to each other than either was to Mac2 macrophages (Fig. 4b), whereas monocytes had a distinct signature from both mature macrophage subsets (Fig. 4c–e). In support of slower replenishment of Mac2 macrophages from recruited monocytes, human uterine CD206^+^ Mac2 macrophages expressed higher levels of Ki-67 than Mac1 macrophages, suggesting greater self-renewal in situ (Extended Data Fig. 6a). As in mice, human uterine Mac1 macrophages exhibited an inflammatory profile, with pathway analysis revealing enrichment of genes involved in programmed cell death, immune activation, cytokine production and neutrophil recruitment (Fig. 4f). Analysis of a selection of genes identified in inflammatory ‘recently recruited’ macrophages found in human endometrial single-cell studies^12–16^ confirmed that both monocytes and Mac1s expressed classic inflammatory genes such as *S100A8*, *S100A9*, *IL1A* and *IL1B* as well as inflammasome components *CASP1* and *NLRP3* and molecules involved in tissue and vascular remodeling, including *TIMP1* and *VEGFA* (Extended Data Fig. 6b).

By contrast, human Mac2 macrophages expressed anti-inflammatory genes (*CD28*^38^*)* and genes associated with alternative activation, endocytosis and efferocytosis (*SELENOP*, *CD163L1* and *S1PR1*^16,39–41^; Fig. 4d). The selection of genes previously used to define human endometrial macrophage subsets in scRNA-seq studies^12,14,15^ suggests a lipid-associated profile in some Mac2 macrophages, with expression of *APOE* and *LIPA* (Extended Data Fig. 6b). Mac2 macrophages were also *FOLR2*^*+*^ (Extended Data Fig. 6b), suggesting a possible ‘tissue-resident’ phenotype. Consistent with our findings in mice, we proposed that this subset was in fact replenished from blood monocytes but at a slower rate than Mac1 macrophages (Fig. 3a). Also in common with our findings in mice, human Mac2 macrophages selectively express *CCL2* (Extended Data Fig. 6b) and thus may be directly involved in monocyte recruitment to the endometrium in humans. Another common feature is human endometrial monocytes being potent producers of TNF protein (Extended Data Fig. 6c).

To explore further the similarity of mouse and human uterine myeloid cells, we constructed homologous gene signatures for both human and mouse Mac1 and Mac2 macrophages and examined their overlap (Fig. 4e). This confirmed that many of the genes involved in tissue repair, angiogenesis and alternative activation of macrophages, which were expressed in murine Mac2 macrophages, were conserved in human Mac2 macrophages (for example, *MERTK*, *SIRPA*, *CD163*, *SPRED1*, *TREM2*, *GAS6*, *NRP1*, *CYFIP1*, *HMOX1* and *STAB1*). As in mice, *ITGAX* encoding CD11c and inflammatory mediators such as *CCL5* and cytotoxicity genes were restricted to human Mac1 macrophages and not expressed by Mac2 macrophages (Fig. 4e). As in mice, the number of human Mac1 macrophages depended on menstrual cycle stage, peaking in the early secretory phase alongside monocyte infiltration (Fig. 4g). In contrast, Mac2 numbers remained consistent through the cycle as in mice. Together, these data show that, despite differences in reproductive cycle physiology, the cyclical changes in uterine monocytes and macrophage populations and their gene expression pathways are conserved between the two species.

### Two functionally distinct subsets of monocytes coexist in the human endometrium

**Fig. 5:**
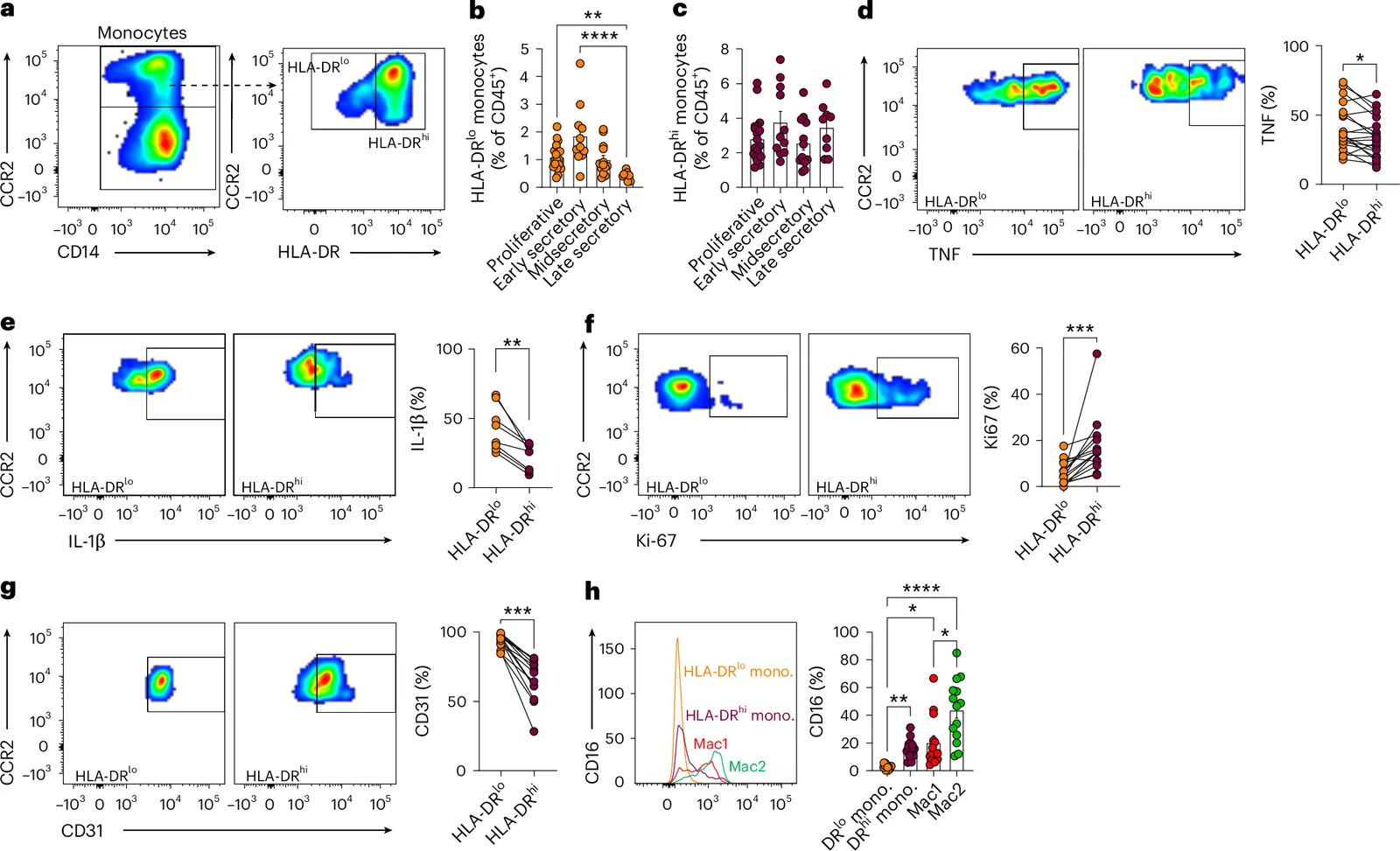
Characterization of two subsets of monocytes in the human endometrium. **a**, Flow cytometry gating strategy with all cells pre-gated on CD45^+^ live CD3^−^CD19^−^CD66b^−^Sig8^−^HLA-DR^+^CD64^+^ cells, monocytes identified as CCR2^+^CD14^+^ cells and then subcategorized into HLA-DR^hi^ and HLA-DR^lo^ subsets of monocytes. **b**, Summary graph demonstrating proportions (of total CD45^+^ cells) of HLA-DR^lo^ monocytes at different menstrual cycle stages. Kruskal–Wallis test was applied. Proliferative, *n* = 18; early secretory, *n* = 11; midsecretory, *n* = 12; and late secretory, *n* = 9. **c**, Proportions (of total CD45^+^ cells) of HLA-DR^hi^ monocytes at different menstrual cycle stages. Kruskal–Wallis test was applied. Proliferative, *n* = 18; early secretory, *n* = 11; mid secretory, *n* = 12; and late secretory, *n* = 9. **d**, Representative flow cytometry plots for intracellular TNF production by monocyte subsets and summary graph showing TNF production by HLA-DR^lo^ and HLA-DR^hi^ monocytes (*n* = 20). A two-tailed Wilcoxon’s matched-pair, signed-rank test was applied. **e**, Representative flow cytometry plots for intracellular IL-1β production by monocyte subsets and summary graph showing proportions of HLA-DR^lo^ and HLA-DR^hi^ monocytes producing IL-1β (*n* = 8). A two-tailed, paired Student’s *t*-test was applied. **f**, Representative flow cytometry plots for intracellular Ki-67 expression by monocyte subsets and summary graph proportions of HLA-DR^lo^ and HLA-DR^hi^ monocytes expressing Ki-67 (*n* = 15). A two-tailed Wilcoxon’s matched-pair, signed-rank test was applied. **g**, Representative flow cytometry plots for CD31 expression by monocyte subsets and summary graph showing proportions of HLA-DR^lo^ and HLA-DR^hi^ monocytes expressing CD31 (*n* = 14). A two-tailed Wilcoxon’s matched-pair, signed-rank test was applied. **h**, Representative flow cytometry histograms showing CD16 expression by monocyte and macrophage subsets and summary graph proportions of HLA-DR^lo^ monocytes, HLA-DR^hi^ monocytes, Mac1 macrophages and Mac2 macrophages expressing CD16 (*n* = 14). Kruskal–Wallis test was applied. All data represent at least two independent experiments. Where Kruskal–Wallis tests have been used *P*_adj_ values are reported from post hoc Dunn’s tests. ^*^P < 0.05, ^**^P < 0.01, ^***^P < 0.001, ^****^P < 0.0001. Data are presented as the median ± IQR in **b**, **c** and **h**. mono., monocyte. Source data

One report has identified two subsets of endometrial monocytes, based on transcriptional profiling^14^. Consistent with this, we identified distinct HLA-DR^hi^ and HLA-DR^lo^ monocytes by flow cytometry (Fig. 5a). HLA-DR^lo^ monocytes accounted for the fluctuations in total monocytes through the menstrual cycle, whereas HLA-DR^hi^ monocytes remained more constant (Fig. 5b,c). These subsets differed functionally, with a greater proportion of HLA-DR^lo^ monocytes producing TNF (Fig. 5d) and interleukin (IL)-1β (Fig. 5e) relative to HLA-DR^hi^ monocytes.

We proposed that HLA-DR^lo^ monocytes have recently been recruited from the bloodstream, whereas HLA-DR^hi^ monocytes may have been present longer in the tissue. In support of this, HLA-DR^hi^ monocytes expressed more Ki-67 (Fig. 5f), indicating proliferation and self-maintenance, whereas HLA-DR^lo^ monocytes expressed high levels of CD31 (Fig. 5g), characteristic of blood monocytes^42^. Although nearly all endometrial monocytes expressed CD14 (Fig. 5a), HLA-DR^lo^ monocytes lacked CD16 expression (Fig. 5h), indicating classic monocyte origin. Low levels of CD16 expression were observed on HLA-DR^hi^ monocytes and Mac1 macrophages (Fig. 5h), whereas a substantially higher proportion of Mac2 macrophages expressed CD16 (Fig. 5h). Thus CD16 expression may reflect tissue residency and differentiation, rather than defining origin from classic and nonclassic monocytes.

### Cytotoxic profile of monocytes and regulation of uterine epithelial cell homeostasis

**Fig. 6:**
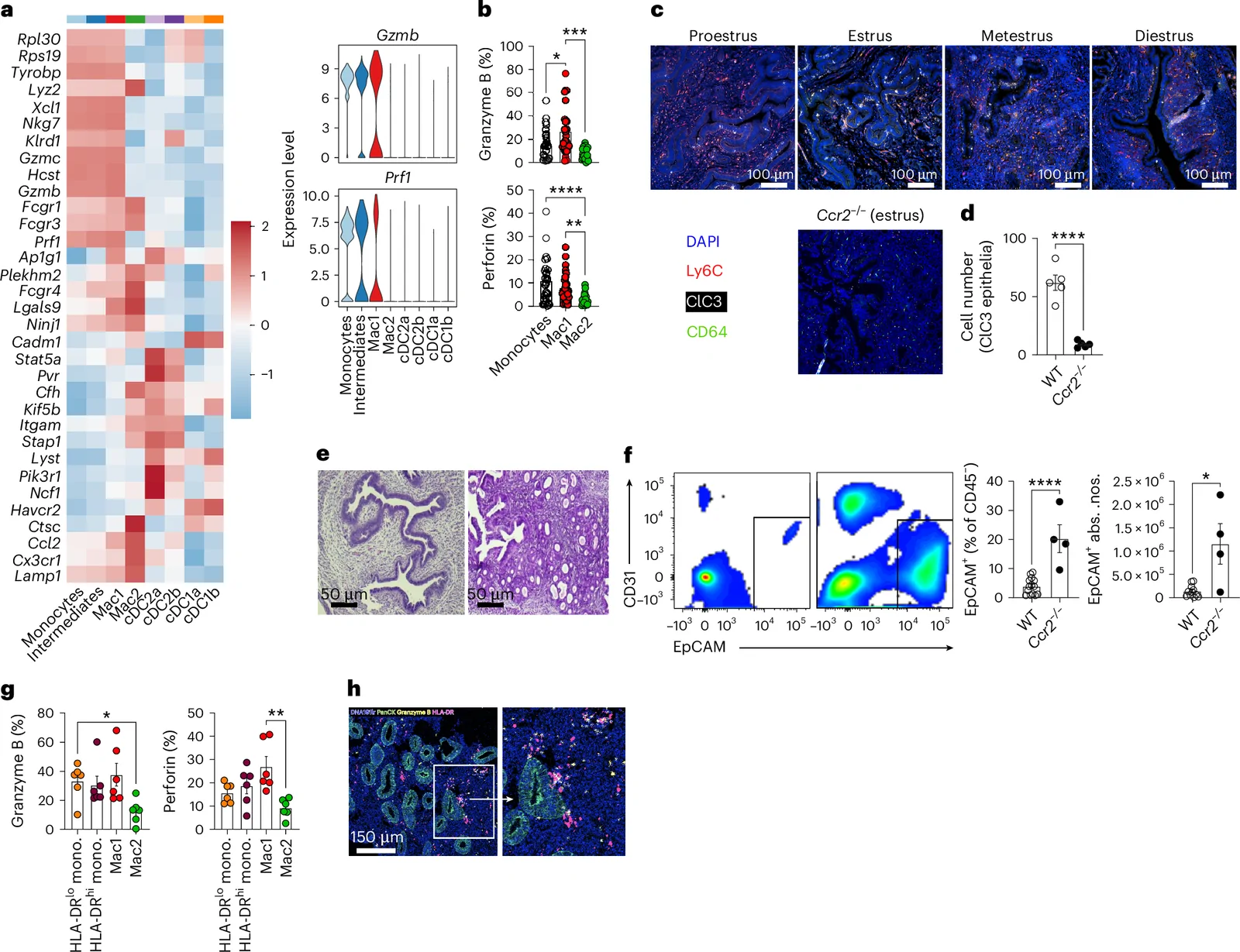
Cytotoxic properties of uterine monocytes and macrophages alongside epithelial cell apoptosis. **a**, Heatmap representing the relative average expression of genes in the GO term ‘Cell killing’ (GO:0001906) in uterine myeloid cell clusters identified by scRNA-seq analysis (murine). Plotted genes were significantly differentially expressed in at least one cluster relative to the other clusters (where *P*_adj_ < 0.01 and absolute log_2_(fold-change) > 1). Violin plots show the expression level of *Gzmb* and *Prf1* in myeloid cell clusters as determined by scRNA-seq analysis. **b**, Summary graphs demonstrating proportions of monocytes and Mac1 and Mac2 macrophages in the mouse uterus expressing granzyme B and perforin protein, as assessed by flow cytometry. For granzyme B one-way ANOVA was applied: monocytes, *n* = 20; Mac1, *n* = 20; and Mac2, *n* = 20. For perforin, Kruskal–Wallis test was applied: monocytes, *n* = 21; Mac1, *n* = 21; and Mac2, *n* = 22. **c**,**d**, Immunofluorescence microscopy images demonstrating Ly6C, CD64 and ClC3 staining in cross-sections of murine uterine horn during proestrus, estrus, metestrus and diestrus in WT mice (**c**) and during estrus in *Ccr2*^*−/−*^ mice (**d**). Images represent several independent experiments with similar results. A summary graph shows numbers of epithelial cells expressing ClC3 in WT (*n* = 5) and *Ccr2*^*−/−*^ mice (*n* = 5) during estrus. A two-tailed, unpaired Student’s *t*-test was applied. **e**, Hematoxylin and eosin staining of cross-sections of murine uterine horn in WT (left) and *Ccr2*^*−/−*^ (right) mice taken during estrus. The images represent several independent experiments with similar results. **f**, Representative flow cytometry plot (during diestrus) with all cells pre-gated on CD45^−^ cells and epithelial cells identified as CD31^−^EpCAM^+^ cells and summary graphs demonstrating proportions (of total CD45^−^ cells) and absolute numbers (per uterus) of EpCAM^+^ cells assessed by flow cytometry after total uterine digest during diestrus in WT (left; *n* = 14) and *Ccr2*^*−/−*^ (right; *n* = 4) mice. A two-tailed, unpaired Student’s *t*-test was applied. **g**, Summary graphs demonstrating proportions of HLA-DR^lo^ monocytes, HLA-DR^hi^ monocytes, Mac1 macrophages and Mac2 macrophages in the human endometrium expressing granzyme B and perforin protein assessed by flow cytometry (*n* = 6). Kruskal–Wallis tests were applied in both cases. **h**, Imaging mass cytometry pictures demonstrating pancytokeratin staining in glandular epithelium and HLA-DR and granzyme B staining in cells clustered next to the glandular epithelium. Sections were taken from endometrial biopsies during the early secretory phase of the menstrual cycle. The images represent five different ROIs within one sample and 13 different biological samples. All data represent at least two independent experiments. For ANOVA and Kruskal–Wallis tests *P*_adj_ values are reported from Tukey’s and Dunn’s post hoc tests, respectively; ^*^P < 0.05, ^**^P < 0.01, ^***^P < 0.001, ^****^P < 0.0001. Data are presented as the mean ± s.e.m. in **b** (granzyme B only), **d** and **f** and as the median ± IQR in **b** (perforin only) and **g**. Source data

Pathway analysis of RNA-seq data indicated cell-killing capacity in mouse and human uterine monocytes and Mac1 macrophages (Figs. 2d and 4f). This was confirmed by gene expression of mouse cells (Fig. 6a), with granzyme B and perforin proteins being expressed at notably higher levels by murine monocytes and Mac1 macrophages relative to Mac2 macrophages (Fig. 6b and Extended Data Fig. 6d). To identify possible target cells, we assessed expression of the apoptotic marker ClC3 in WT and *Ccr2*^*−/−*^ mice that are deficient in uterine monocytes (Extended Data Fig. 5a,b) with reduced Mac1 macrophages (Fig. 3c). Tissue immunofluorescence imaging showed that the expansion of Ly6C^+^ monocytes increased in the WT uterus during proestrus (Fig. 6c) and was followed 24 h later in estrus by a clear increase in ClC3, mostly in the epithelium (Fig. 6c and Extended Data Fig. 6e,f). The expression of ClC3 did not occur in estrus in *Ccr2*^*−/−*^ mice (Fig. 6c,d and Extended Data Fig. 6g), linking monocytes or Mac1 macrophages with epithelial cell apoptosis.

In parallel, *Ccr2*^*−/−*^ mice exhibited structural abnormalities in the uterus, including denser epithelial architecture (Fig. 6e). Flow cytometry revealed extensive fluctuations in epithelial cell numbers during the estrus cycle in WT mice, peaking during estrus (when ClC3 expression is maximal) before declining during metestrus 24 h later and remaining very low during diestrus (Extended Data Fig. 6h). This reduction in epithelial cells during diestrus was not observed in *Ccr2*^*−/−*^ mice, consistent with reduced epithelial cell apoptosis in the absence of monocytes (Fig. 6f). In humans, gene (Fig. 4e) and protein expression of perforin and granzyme B (Fig. 6g and Supplementary Fig. 1a) suggested that similar processes may be occurring in the human uterus where monocytes and Mac1 macrophages produced similar levels of granzyme B and perforin, but with very little being found in Mac2 macrophages (Fig. 6g). Granzyme B appeared to be more highly expressed than perforin in all cases (Fig. 6g) and imaging mass cytometry showed clustering of granzyme B-expressing HLA-DR^+^ monocytes or macrophages next to the glandular epithelial structures (Fig. 6h and Supplementary Fig. 1b). Antibody efficacy for granzyme B was confirmed with immunofluorescence microscopy on a different sample (Supplementary Fig. 1c).

### Interactions between uterine monocytes and fibroblasts in ECM turnover

**Fig. 7:**
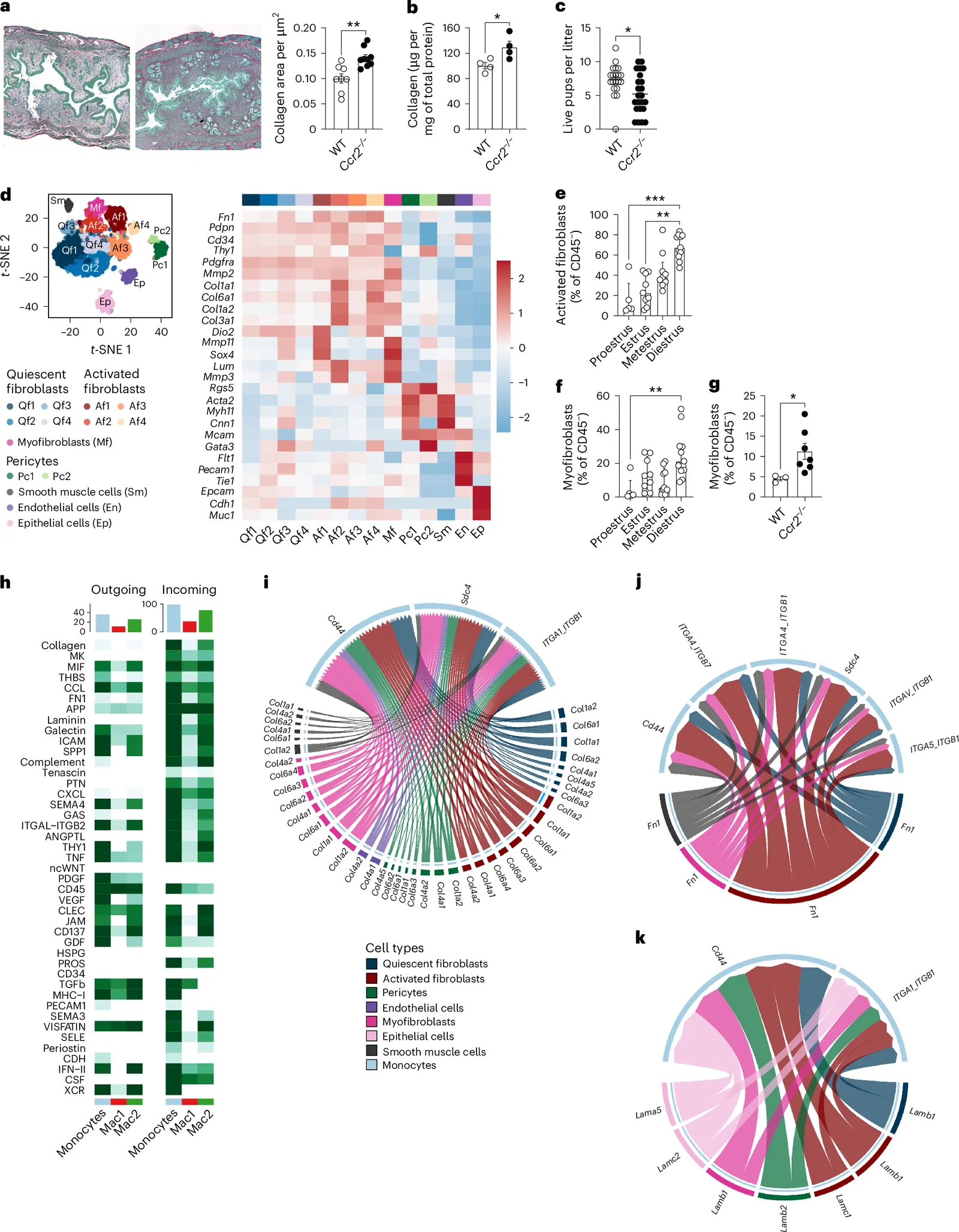
Crosstalk of monocytes, fibroblasts and ECM. **a**, Images showing Picrosirius Red staining representing collagen (red) and Fast Green staining noncollagenous proteins. The summary graph shows collagen fibers per square micrometer of tissue in WT littermate controls (left; *n* = 8) and *Ccr2*^*−/−*^ mice (right; *n* = 9) taken during proestrus. An unpaired Student’s *t*-test was applied. Collagen was measured per square micrometer at five ROIs per tissue section (0.5 μm × 0.5 μm in size). **b**, Summary graph showing the amount of collagen protein per mg of tissue measured by hydroxyproline assay from WT (*n* = 4) and *Ccr2*^*−/−*^(*n* = 4) mice. A two-tailed, unpaired Student’s *t*-test was applied. **c**, Summary graph demonstrating the number of live pups born per litter in WT (*n* = 20) and *Ccr2*^*−/−*^ (*n* = 23) mice bred inhouse at the University of Glasgow. A two-tailed Mann–Whitney *U*-test was applied. **d**, ScRNA-seq analysis of sorted and pooled CD45^−^ uterine cells. Dimensionality reduction of identified CD45^−^ cell clusters was performed using *t*-SNE (*n* = 16 across all cycle stages). The heatmap represents the relative average expression of selected genes between clusters used to identify cell populations. **e**,**f**, Summary graph of flow cytometric analyses showing proportions of activated fibroblasts within CD45^−^ cells (CD45^−^ live EpCAM^−^CD31^−^MCAM^−^PDGFRα^+^CD90^+^ cells; a Kruskal–Wallis test: proestrus, *n* = 5; estrus, *n* = 9; metestrus, *n* = 9; and diestrus, *n* = 11) (**e**) and myofibroblasts (CD45^−^ live EpCAM^−^CD31^−^MCAM^−^PDGFRα^+^CD90^−^ cells; Kruskal–Wallis test, ^**^P < 0.01 in WT mice at different estrus cycle stages: proestrus, *n* = 5, estrus; *n* = 9, metestrus; *n* = 9; and diestrus, *n* = 11) (**f**). **g**, Summary graph of flow cytometric analyses showing proportions of myofibroblasts within CD45^−^ cells in WT and *Ccr2*^*−/−*^ mice during proestrus. A two-tailed Mann–Whitney *U*-test was applied. Littermate controls (WT), *n* = 3; *Ccr2*^*−/−*^, *n* = 7. **h**, Heatmap of outgoing and incoming signaling patterns between monocytes, Mac1 macrophages and Mac2 macrophages and ECM-producing CD45^−^ cells, based on their gene expression signatures. **i**, Chord plot of predicted collagen interactions between CD45^−^ cell subsets and monocytes based on expression of *Cd44*, *Sdc4* and *Itga1* or *Itgb1*. **j**, Chord plot of predicted fibronectin (*Fn1*) interactions between CD45^−^ cell subsets and monocytes based on expression of *Cd44*, *Itga4* or *Itgb7*, *Itga4* or *Itgb1*, *Sdc4*, *Itgav* or *Itgb1* and *Itga5* or *Itgb1*. **k**, Chord plot of predicted laminin interactions between CD45^−^ cell subsets and monocytes based on expression of *Cd44* and *Itga1* or *Itgb1*. All flow cytometry data represent at least two independent experiments. Where Kruskal–Wallis tests have been used, *P*_adj_ values are reported from post hoc Dunn’s tests: ^*^P < 0.05, ^**^P < 0.01, ^***^P < 0.001, ****P < 0.0001. Data are presented as the mean ± s.e.m. in **b** and as the median ± IQR in **c**, **e**, **f** and **g**. In **i**–**k**, integrins are shown as heterodimer pairs, with underscore separating each integrin subunit. Source data

Epithelial remodeling and ECM synthesis and turnover are required for normal functioning of all tissues^43^; in uterus shedding, regeneration and remodeling through reproductive cycles are marked by dynamic changes in the ECM^44^. To investigate whether infiltrating monocytes and macrophages are involved in the regulation of ECM turnover in the uterus, we first assessed total collagen deposition in WT and *Ccr2*^*−/−*^ mice, revealing excess collagen deposition in the uterus of *Ccr2*^*−/−*^ mice, especially during the proestrus phase of the reproductive cycle (Fig. 7a,b and Extended Data Fig. 6i). In parallel, there were breeding abnormalities in *Ccr2*^*−/−*^ mice with fewer live pups per litter compared to WT mice (Fig. 7c).

**Extended Data Fig. 7:**
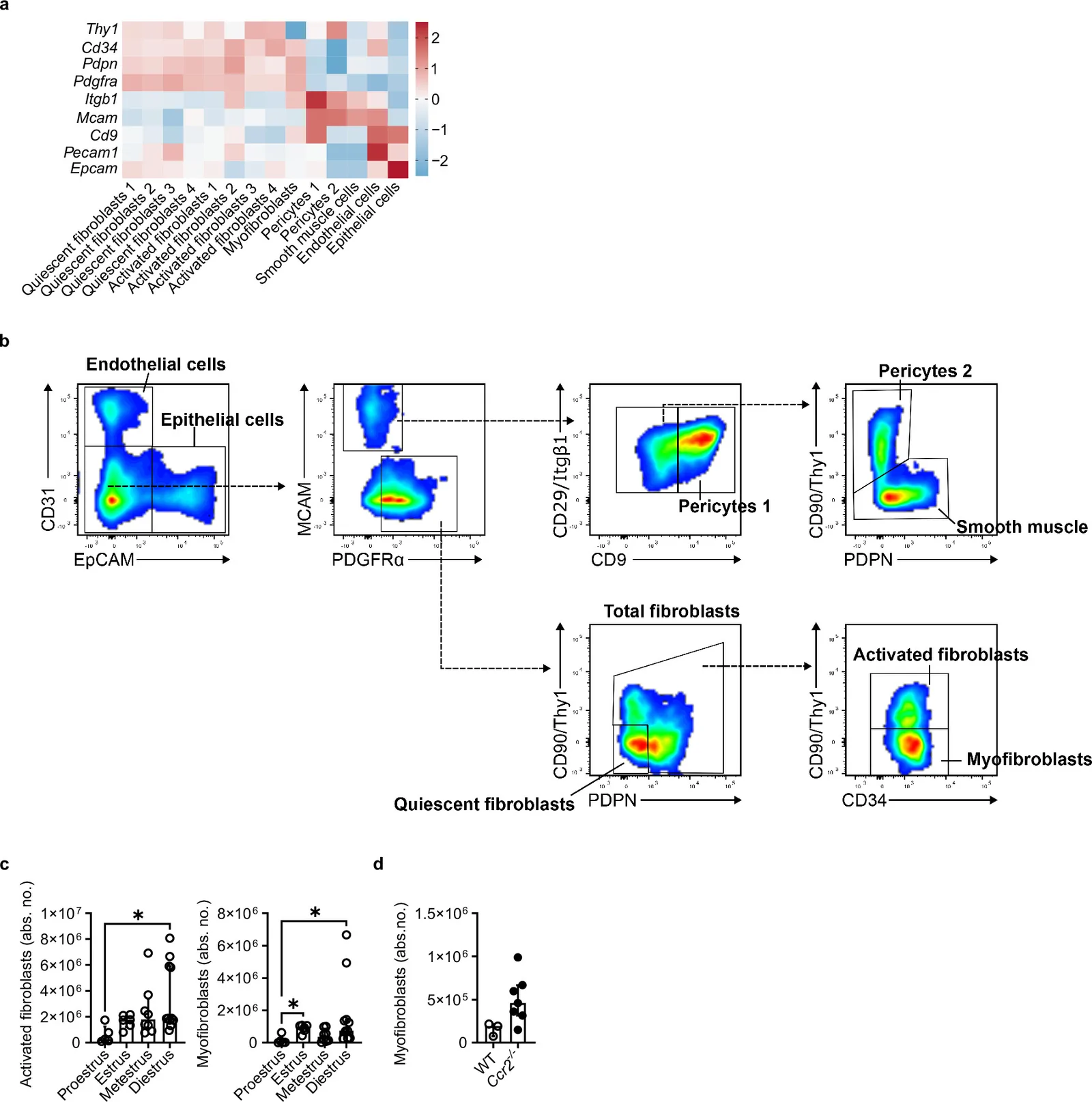
Flow cytometry gating strategies for uterine CD45^−^ cell subsets in mice. **a**) Heatmap representing relative average gene expression of selected genes in CD45^−^ cell clusters identified in single cell RNAseq analysis. **b**) Flow cytometry gating strategy for epithelial cells, endothelial cells, pericytes, smooth muscle cells, quiescent fibroblasts, activated fibroblasts and myofibroblasts in the mouse uterus. **c**) Absolute numbers (per uterus) of activated fibroblasts and myofibroblasts at different estrous cycle stages (proestrus: n = 5, estrus: n = 6, metestrus: n = 8, diestrus: n = 11). Kruskal-Wallis tests were applied in both cases. **d**) Summary data demonstrating absolute numbers (per uterus) of myofibroblasts in the mouse uterus of WT (n = 3) and *Ccr2*^*−/−*^ (n = 7) mice during proestrus. In all cases, at least two independent experiments were carried out. For Kruskal-Wallis tests adjusted p values are reported from Dunn’s *post hoc* tests respectively. *p < 0.05, **p < 0.01, ***p < 0.001, ****p < 0.0001. Data are presented as mean±SEM in **d**. Data are presented as median±IQR in **c**. Source data

As fibroblasts are the primary producers of collagen and other ECM proteins in most tissues^43^, we performed scRNA-seq on CD45^−^ cells from murine uteri pooled from all cycle stages. This revealed the presence of fibroblasts, smooth muscle cells, pericytes and epithelial and endothelial cells (Fig. 7d). Fibroblasts were characterized by their expression of *Pdgfra* and lack of expression of epithelial (*Epcam*, *Cdh1* and *Muc1*), endothelial (*Pecam1*, *Tie1* and *Flt1*), smooth muscle cell (*Acta2*, *Myh11*, *Cnn1* and *Mcam*) and pericyte markers (*Acta2* and *Rgs5*) (Fig. 7d). Fibroblasts were classed as activated or quiescent based on their expression of ECM proteins (*Fn1*, *Col1a1*, *Col6a1*, *Col1a2*, *Col3a1* and *Lum*) and matrix remodeling proteins (*Mmp2*, *Mmp3* and *Mmp11*). Myofibroblasts were identified as a subset of activated fibroblasts expressing *Acta2* and *Myh11* and high levels of *Lum* but without expression of *Thy1* or *Rgs5* (Fig. 7d). Unbiased clustering of genes specific to these subsets can be seen in Supplementary Fig. 2. Based on our scRNA-seq data, we developed a flow cytometry panel for identifying these CD45^−^ cell subsets (Extended Data Fig. 7a,b). Activated fibroblasts and myofibroblasts fluctuated throughout the murine estrus cycle, with both peaking during diestrus (Fig. 7e,f and Extended Data Fig. 7c). Myofibroblasts expressed particularly high levels of ECM proteins (including collagens and lumican) and they were substantially increased in *Ccr2*^*−/−*^ mice compared with WT controls (Fig. 7g and Extended Data Fig. 7d), consistent with elevated collagen deposition seen in these mice (Fig. 7a, b).

**Extended Data Fig. 8:**
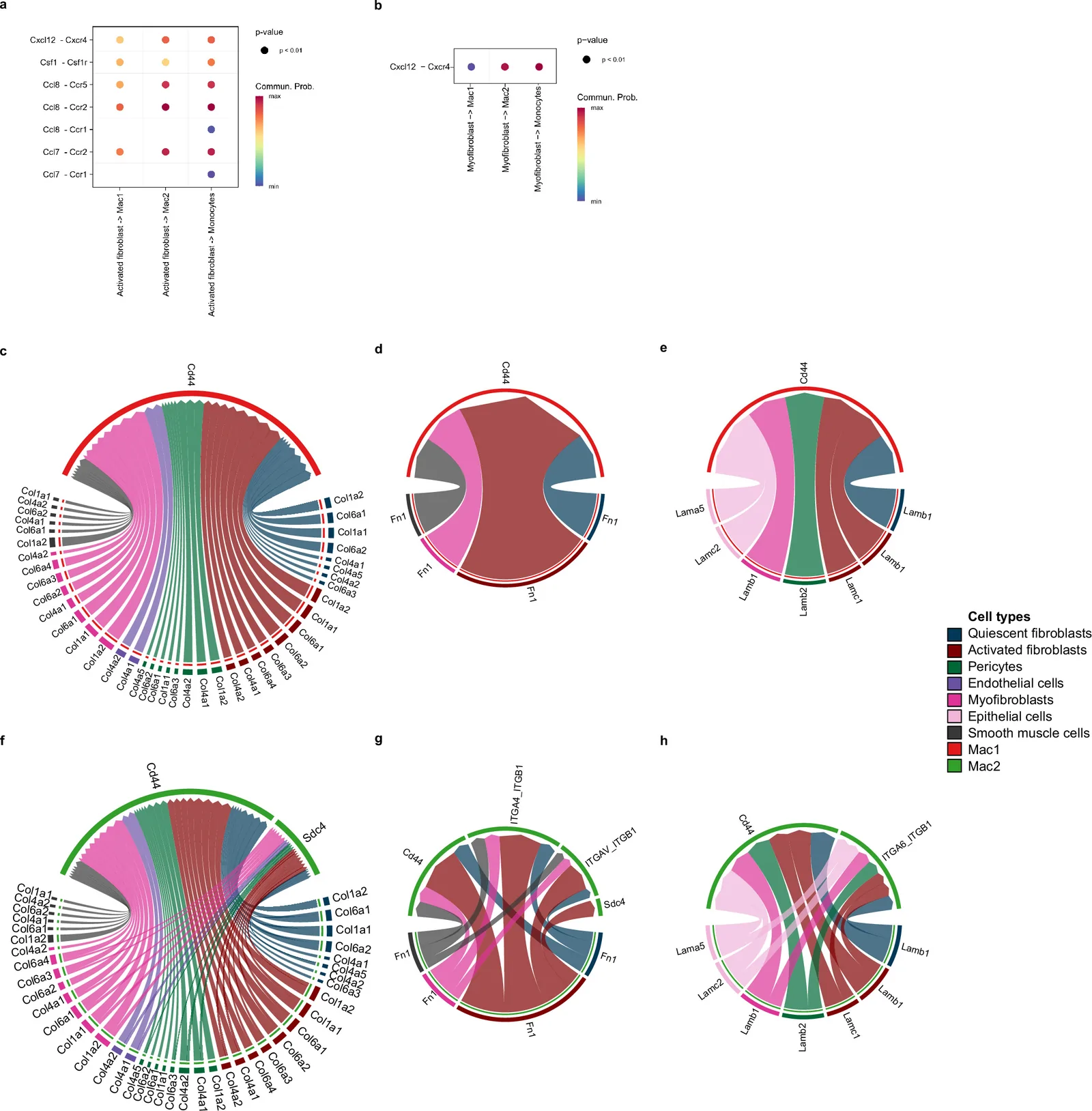
Predicted communication patterns between monocytes, macrophages and fibroblasts. **a**) Heatmap demonstrating the most significant communication patterns related to monocyte recruitment and differentiation, between activated fibroblasts and myeloid cell subsets computed by CellChat. **b**) Heatmap demonstrating the most significant communication patterns related to monocyte recruitment and differentiation, between activated myofibroblasts and myeloid cell subsets computed by CellChat. **c**) Chord plot of predicted collagen interactions between CD45^−^ cell subsets and Mac1 macrophages based on expression of *CD44*. **d**) Chord plot of predicted fibronectin (*FN1*) between CD45^−^ cell subsets and Mac1 macrophages based on expression of *CD44*. **e**) Chord plot of predicted laminin interactions between stromal cell subsets and Mac1 macrophages based on expression of *CD44*. **f**) Chord plot of predicted collagen interactions between CD45^−^ cell subsets and Mac2 macrophages based on expression of *Cd44* and *Sdc4*. **g**) Chord plot of predicted fibronectin (Fn1) between CD45^−^ cell subsets and Mac2 macrophages based on expression of Cd44, Sdc4, Itgav/Itgb1 and Itga4/Itgb. **h**) Chord plot of predicted laminin interactions between stromal cell subsets and Mac2 macrophages based on expression of Cd44 and Itgb6/Itga1. Source data

CellChat analysis^45^ of our myeloid-enriched and CD45^−^ datasets predicted that monocytes are key cells interacting with cells producing ECM proteins and adhesion molecules involved in tissue remodeling (Fig. 7h). Monocyte interactions with collagens (Fig. 7i), fibronectin (Fig. 7j) and laminin (Fig. 7k) were predicted via their expression of *Cd44* and *Sdc4* and integrin heterodimers *Itga1/Itgb1*, *Itga5/Itgb1*, *Itgav/Itgb1*, *Itga4/Itgb1* and *Itga4/Itgb7*. These patterns are typical of macrophages involved in tissue repair^46^, but in our hands they were unexpectedly most strongly associated with uterine monocytes and, to a lesser extent, Mac2 macrophages (Fig. 7h and Extended Data Fig. 8c–h). Interactions between fibroblasts and monocytes are likely to be bidirectional, because activated fibroblasts expressed a range of chemokines involved in monocyte recruitment to tissues (*Cxcl12*, *Ccl8* and *Ccl7*), as well as *Csf1* which is required for monocyte differentiation into macrophages (Extended Data Fig. 8a). Myofibroblasts may also be involved in monocyte chemotaxis along the CXCL12–CXCR4 axis (Extended Data Fig. 8b). Thus uterine monocytes may contribute to tissue remodeling as well as inflammation or cytotoxicity.

Mac2 macrophages also expressed tissue-remodeling genes, but these were distinct from those in monocytes (Fig. 7h and Extended Data Figs. 2 and 3a), implicating processes such as lipolysis (*Lipa*), cell contractility (*Rock2*), Wnt signaling (*Lrp6*), fibrillar adhesion and actin remodeling (*Tns3*), clearance of apoptotic cells (*Itgav, Itgb5* (refs. ^47,48^) and *Axl*) and angiogenesis (*Vegfa*) (Extended Data Fig. 2c). In addition, some molecules found on monocytes were not expressed by Mac2 macrophages (for example, *Itga1* and *Itga5* (Supplementary Fig. 3c)). There were also predicted interactions between Mac1 macrophages and ECM (Extended Data Fig. 8c–e), but this was limited to *Cd44* expression on the Mac1 macrophages. Overall, these data indicate that monocytes and Mac2 macrophages have distinct roles in uterine tissue remodeling.

### Dysregulation of epithelial proliferation, ECM, monocytes and macrophages in mouse and human uterine fibrosis

**Extended Data Fig. 9:**
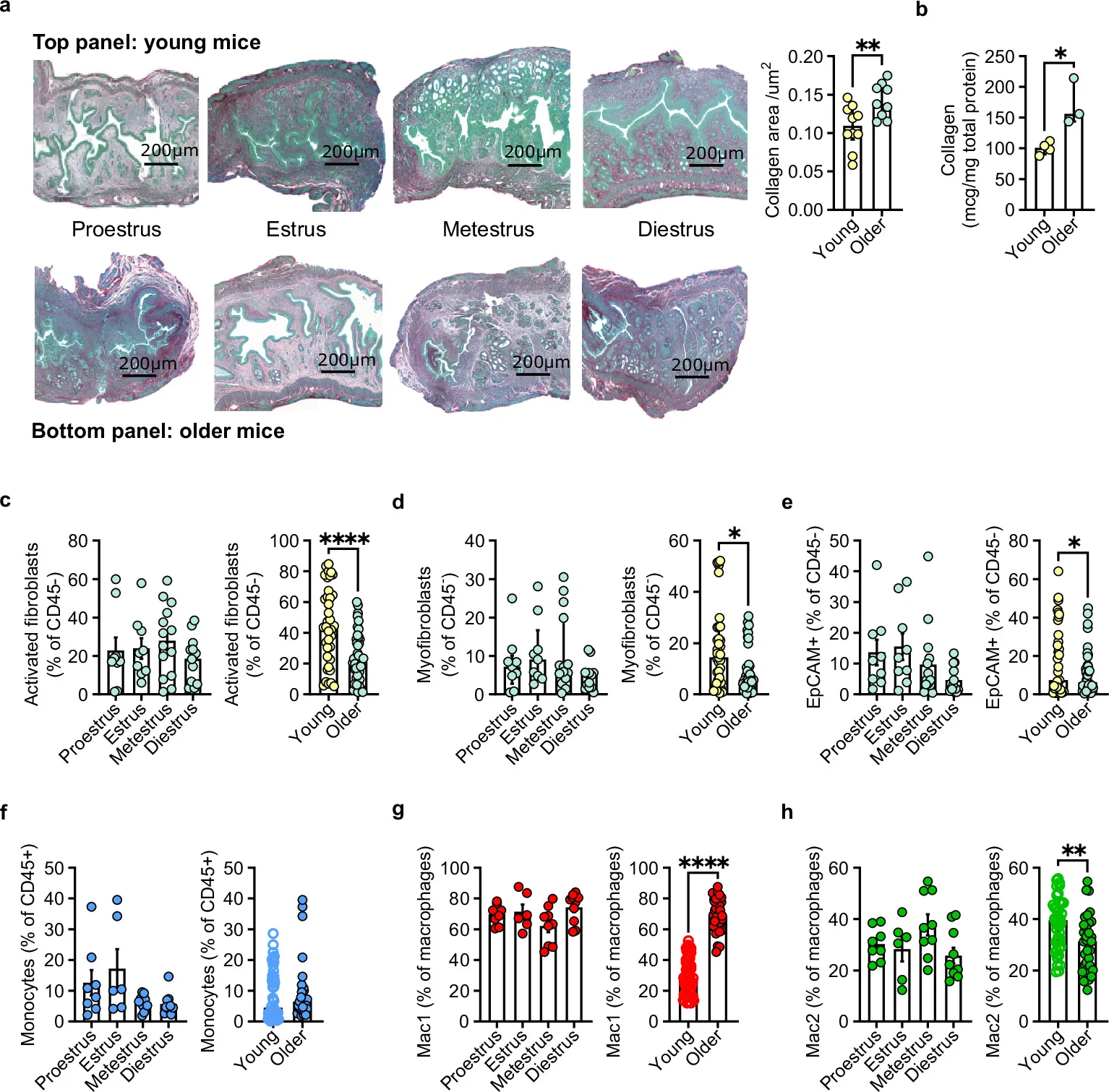
Uterine fibrosis in older mice. **a**) Images showing picrosirius red staining representing collagen (red) and fast green staining non collagenous proteins. Summary graph shows collagen measured per μm^2^ at five regions of interest (ROI) per tissue section/biological replicate (0.5 μm × 0.5 μm in size), across all cycle stages combined in young ( < 16 weeks; n = 10) and older (9-12 months; n = 9) mice. Two-tailed unpaired t-test was applied. **b**) Summary graph showing collagen measured by hydroxyproline assay from young (n = 4) and older (n = 3) WT mice during proestrus. Two-tailed unpaired t test was applied. **c**) Summary graph of flow cytometric analyses showing proportions of activated fibroblasts (CD45^−^live EpCAM^−^CD31^−^MCAM^−^PDGFRα^+^CD90^+^ cells) in older mice at individual estrous cycle stages (proestrus n = 9, estrus n = 9, metestrus n = 14, diestrus n = 13) and total activated fibroblasts in young (n = 37) and older mice (n = 46), data pooled across reproductive cycle stages. Two-tailed Mann-Whitney test was applied. **d**) Summary graph of flow cytometric analyses showing proportions of myofibroblasts within CD45^−^ cells (CD45^−^live EpCAM^−^CD31^−^MCAM^−^PDGFRα^+^CD90^−^ cells) in older mice at individual estrous cycle stages (proestrus n = 9, estrus n = 9, metestrus n = 14, diestrus n = 13,) and total myofibroblasts in young (n = 37) and older mice (n = 46), data pooled across reproductive cycle stages. Two-tailed Mann-Whitney test was applied. **e**) Summary graph of flow cytometric analyses showing proportions of epithelial cells within CD45^−^ cells (CD45^−^live EpCAM^+^CD31^−^ cells) in older mice at individual estrous cycle stages (proestrus n = 9, estrus n = 9, metestrus n = 14, diestrus n = 13) and total epithelial cells in young (n = 37) and older mice (n = 46), data pooled across reproductive cycle stages. Two-tailed unpaired t test was applied. **f**) Summary graph of flow cytometric analyses showing proportions of monocytes within CD45^+^ cells (CD45^+^ live Ly6G^−^SigF^−^CD11b^+^CD64^+^Ly6C^+^ cells) in older mice at individual estrous cycle stages (proestrus n = 8, estrus n = 6, metestrus n = 9, diestrus n = 10) and total monocytes in young (n = 54) and older mice (n = 33), data pooled across reproductive cycle stages. **g**) Summary graph of flow cytometric analyses showing proportions of Mac1 macrophages (CD45^+^ live Ly6G^−^SigF^−^CD11b^+^CD64^+^Ly6C^−^CD11c^+^CD206^−^ cells) in older mice at individual estrous cycle stages (proestrus n = 8, estrus n = 6, metestrus n = 9, diestrus n = 10) and total Mac1 macrophages in young (n = 54) and older mice (n = 33), data pooled across reproductive cycle stages. Two-tailed Mann-Whitney test was applied. **h**) Summary graph of flow cytometric analyses showing proportions of Mac2 macrophages (CD45^+^ live Ly6G^−^SigF^−^CD11b^+^CD64^+^Ly6C^−^CD11c^−^CD206^+^ cells) in older mice at individual estrous cycle stages (proestrus n = 8, estrus n = 6, metestrus n = 9, diestrus n = 10) and total Mac2 macrophages in young (n = 46) and older mice (n = 33), data pooled across reproductive cycle stages. Two-tailed unpaired t-test was applied. All data are representative of at least two independent experiments. *p < 0.05, **p < 0.01, ****p < 0.0001. Data are presented as mean±SEM in **a**, **b**, **c**, **d**, **e**, **f**, **h**. Data are presented as median±IQR in **g**. Source data

Excess deposition of collagen and dysregulation of ECM is a hallmark feature of tissue fibrosis^43^. To explore the role of monocytes in uterine fibrosis, we first used an established mouse model of fibrosis, which spontaneously develops in the uterus during aging^23^. We observed excess collagen deposition in older (9–12 months old) mice (Extended Data Fig. 9a,b) associated with a loss of the cyclic variation in activated fibroblasts (Extended Data Fig. 9c,d and Supplementary Fig. 4b,c), monocytes (Extended Data Fig. 9f and Supplementary Fig. 4e) and macrophages (Extended Data Fig. 9g,h and Supplementary Fig. 4f,h) seen in younger mice. There was a striking increase in monocytes and proinflammatory (Mac1) macrophages (Extended Data Fig. 9f,g and Supplementary Fig. 4e,f), alongside a decline in epithelial cells (Extended Data Fig. 9e and Supplementary Fig. 4d), activated fibroblasts (Extended Data Fig. 9c,d and Supplementary Fig. 4b,c) and pro-repair Mac2 macrophages (Extended Data Fig. 9h and Supplementary Fig. 4g). The columnar epithelium lining the endometrial glands (glandular epithelium) was thinner in older mice, particularly during estrus (Extended Data Fig. 9a and Supplementary Fig. 4a).

**Fig. 8:**
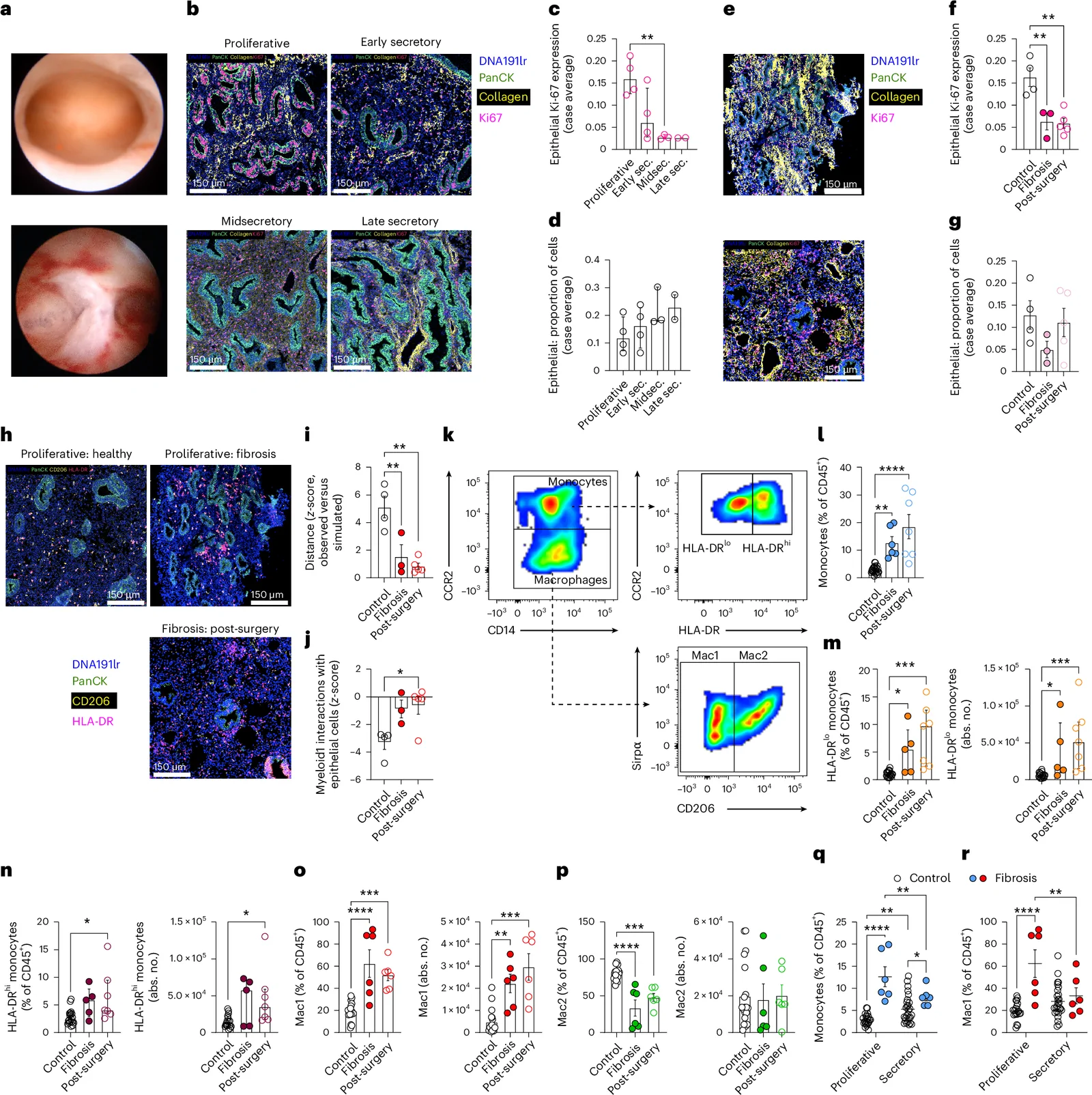
Human endometrial fibrosis (Asherman’s syndrome). **a**, Top: direct visualization of a normal uterine cavity and a normal endometrium during hysteroscopy. Bottom: obliteration of the uterine cavity caused by dense fibrotic bands replacing normal endometrium seen in Asherman’s syndrome. **b**, Imaging mass cytometry pictures demonstrating pancytokeratin staining in glandular epithelium, alongside type 1 collagen and Ki-67 staining in sections taken from endometrial biopsies at different stages of the menstrual cycle in healthy controls. The images represent 5 different ROIs within one sample and 13 biological replicates. **c**, Summary graph demonstrating numbers of epithelial cells expressing pancytokeratin at different stages of the menstrual cycle (proliferative, *n* = 4; early secretory, *n* = 4; mid secretory, *n* = 3; and late secretory, *n* = 2). A one-way ANOVA was applied. **d**, Proportions of epithelial cells and total cells at different stages of the menstrual cycle (proliferative, *n* = 4; early secretory, *n* = 4; mid secretory, *n* = 3; and late secretory, *n* = 2). A Kruskal–Wallis test was applied. **e**, Imaging mass cytometry pictures demonstrating pancytokeratin staining in glandular epithelium, alongside type 1 collagen and Ki-67 staining in sections taken from endometrial biopsies at the proliferative phase of the menstrual cycle in patients with endometrial fibrosis (Asherman’s syndrome) (top) and in patients with Asherman’s syndrome post-surgery (bottom). The images represent five different ROIs within one sample and three biological replicates pre-surgery and five biological replicates post-surgery. **f**, Summary graph demonstrating numbers of pancytokeratin^+^ epithelial cells expressing Ki-67 in sections taken from endometrial biopsies from healthy controls in the proliferative stage (*n* = 4), and in patients with Asherman’s syndrome pre-surgery in the proliferative stage (*n* = 3) and post-surgery (*n* = 5). A one-way ANOVA was applied. **g**, Summary graph demonstrating proportions of epithelial cells of total cells in healthy controls in the proliferative stage (*n* = 4), and in patients with Asherman’s syndrome pre-surgery in the proliferative stage (*n* = 3) and post-surgery (*n* = 5). A one-way ANOVA was applied. **h**, Imaging mass cytometry pictures demonstrating pancytokeratin staining in glandular epithelium, alongside HLA-DR (macrophages) and CD206 (Mac2 macrophages) in sections taken from endometrial biopsies from healthy controls in the proliferative stage, and in patients with Asherman’s syndrome pre-surgery in the proliferative stage and post-surgery. **i**, Summary graph demonstrating proximity of CD206^−^HLA-DR^+^ Mac1 macrophages or monocytes to pancytokeratin^+^ epithelial cells assessed by average distance measured between cell types, in healthy controls in the proliferative stage (*n* = 4), and in patients with Asherman’s syndrome pre-surgery in the proliferative stage (*n* = 3) and post-surgery (*n* = 5). A one-way ANOVA was applied. **j**, Summary graph demonstrating predicted interactions between CD206^−^HLA-DR^+^ Mac1 macrophages or monocytes and pancytokeratin^+^ epithelial cells, in healthy controls in the proliferative stage (*n* = 4), and in patients with Asherman’s syndrome pre-surgery in the proliferative stage (*n* = 3) and post-surgery (*n* = 5). A one-way ANOVA was applied. **k**, Flow cytometry gating strategy with all cells pre-gated on CD45^+^ live CD3^−^CD19^−^CD66b^−^Sig8^−^HLA-DR^+^CD64^+^ cells, monocytes identified as CCR2^+^CD14^+^ cells and then subcategorized into HLA-DR^hi^ and HLA-DR^lo^ subsets of monocytes, and macrophages identified as CCR2^−^CD14^+^ cells and then subcategorized into CD206^−^ Mac1 macrophage and CD206^+^ Mac2 macrophages. An example is shown from a patient with Asherman’s syndrome. **l**, Flow cytometry data summary graph demonstrating proportions of monocytes within CD45^+^ cells in the endometrium of healthy controls in the proliferative stage (*n* = 22), and in patients with Asherman’s syndrome pre-surgery in the proliferative stage (*n* = 6) and post-surgery (n = 7). A one-way ANOVA was applied. **m**, Summary graphs demonstrating proportions within CD45^+^ cells and absolute numbers of HLA-DR^lo^ monocytes in healthy controls in the proliferative stage (*n* = 19), and in patients with Asherman’s syndrome pre-surgery in the proliferative stage (*n* = 5) and post-surgery (*n* = 7). Kruskal–Wallis tests were applied in both cases. **n**, Summary graphs demonstrating proportions within CD45^+^ cells and absolute numbers (per mg of tissue) of HLA-DR^hi^ monocytes in healthy controls in the proliferative stage (*n* = 19), and in patients with Asherman’s syndrome pre-surgery in the proliferative stage (*n* = 5) and post-surgery (*n* = 7). Kruskal–Wallis tests were applied in both cases. **o**, Summary graphs demonstrating proportions within CD45^+^ cells and absolute numbers (per milligram of tissue) of Mac1 macrophages in healthy controls in the proliferative stage (*n* = 20), and in patients with Asherman’s syndrome pre-surgery in the proliferative stage (*n* = 6) and post-surgery (*n* = 6). A one-way ANOVA was applied for proportions and a Kruskal–Wallis test for absolute numbers. **p**, Summary graphs demonstrating proportions within CD45^+^ cells and absolute numbers (per mg of tissue) of Mac2 macrophages in healthy controls in the proliferative stage (*n* = 20), and in patients with Asherman’s syndrome pre-surgery in the proliferative stage (*n* = 6) and post-surgery (n = 6). A one-way ANOVA was applied for proportions. **q**, Summary graphs demonstrating proportions of monocytes within CD45^+^ cells in healthy controls and patients with Asherman’s syndrome in the proliferative and secretory stages of the menstrual cycle (healthy proliferative, *n* = 22; Asherman’s syndrome proliferative, *n* = 6; healthy secretory, *n* = 27; and Asherman’s syndrome secretory, *n* = 6). A two-way ANOVA was applied. **r**, Summary graphs demonstrating proportions of Mac1 macrophages within CD45^+^ cells in healthy controls and in patients with Asherman’s syndrome in the proliferative and secretory stages of the menstrual cycle (healthy proliferative, *n* = 20; Asherman’s syndrome proliferative, *n* = 6; healthy secretory, *n* = 27; and Asherman’s syndrome secretory, *n* = 6). A two-way ANOVA was applied. All data represent at least two independent experiments. For all Hyperion analyses, case average refers to the average of five different ROIs per sample. For one-way ANOVA and Kruskal–Wallis tests, *P*_adj_ values are reported from Tukey’s and Dunn’s post hoc tests, respectively. For two-way ANOVA, *P*_adj_ values are reported from Tukey’s post hoc tests. ^*^P < 0.05, ^**^P < 0.01, ^***^P < 0.001, ^****^P < 0.0001. Data are presented as the mean ± s.e.m. in **c**, **f**, **g**, **i**, **j**, **l**, **o** (Mac1 macrophages, percentage of CD45^+^), **p**, **q** and **r** and as the median ± IQR in **d**, **m**, **n** and **o** (Mac1 absolute numbers). Source data

**Extended Data Fig. 10:**
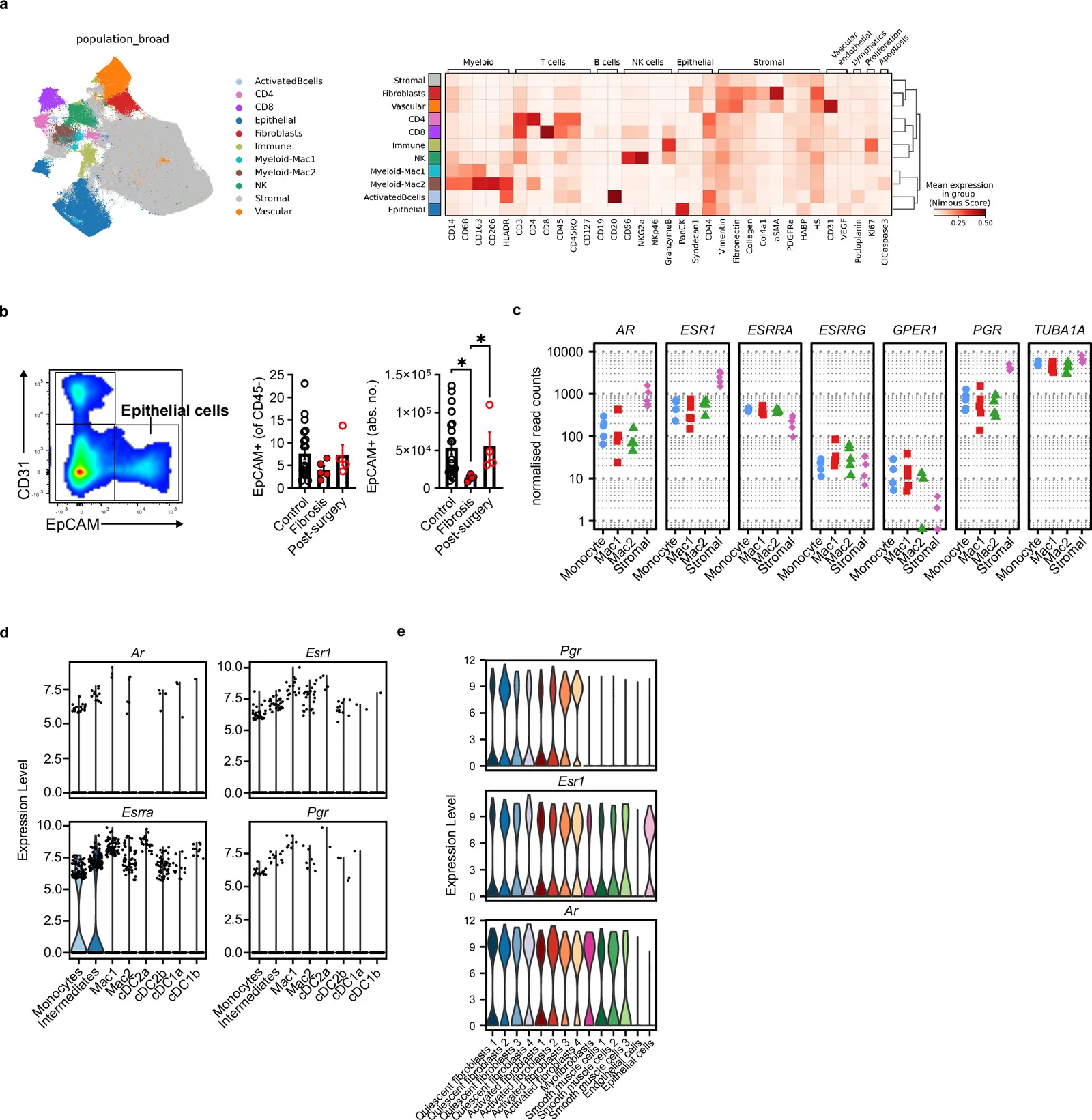
Hyperion, flow cytometry and single cell RNAseq. **a**) Imaging mass cytometry analysis characterising a network of immune and structural cells in the human endometrium revealed by expression patterns of a variety of surface molecules. **b**) Flow cytometry gating strategy for epithelial cells in the human endometrium and summary data demonstrating proportions (of CD45^−^ cells) and absolute numbers (per mg tissue) of EpCAM^+^ epithelial cells in healthy controls (proliferative phase; n = 21) and patients with Asherman Syndrome pre- (proliferative phase; n = 4) and post-surgery (n = 4). Absolute numbers: One way ANOVA was applied. **c**) Normalised read counts comparing gene expression of *AR, ESR1, ESRRA, ESRRG, GPER1*, and *PGR* between FACS-sorted human monocytes, Mac1 macrophages (labelled here as M1), Mac2 macrophages (labelled here as M2) and total stromal cells (bulk RNSseq data). *TUB1A* is presented as a reference housekeeping gene. **d**) Violin plots showing the distribution of expression of *AR, ESR1* and *PGR* within the uterine myeloid cell clusters quantified from mouse single cell RNAseq data. **e**) Violin plots showing the distribution of expression of *AR, ESR1* and *PGR* within the uterine CD45^−^ cell clusters quantified from mouse single cell RNAseq data. Source data

Endometrial fibrosis in humans (Asherman’s syndrome) is characterized by intrauterine adhesions resulting in an obliterated uterine cavity (Fig. 8a), predisposing to infertility and miscarriage^49,50^. We used imaging mass cytometry (Hyperion) to interrogate the spatial relationships between monocytes or macrophages and structural features of the uterus in health and Asherman’s syndrome (Extended Data Fig. 10a). High levels of proliferation (Ki-67) within the glandular epithelium occurred during the proliferative phase, decreasing through the menstrual cycle (Fig. 8b,c). However epithelial cell numbers increased throughout the menstrual cycle (Fig. 8d), likely reflecting the progressive complexity of the glandular network. In contrast, the glandular epithelium showed very little Ki-67 in Asherman’s syndrome (Fig. 8e,f) associated with a reduction in epithelial cells (Fig. 8g and Extended Data Fig. 10b) and similar to uterine fibrosis in mice. Epithelial cell numbers (Extended Data Fig. 10b), structural abnormalities and excess collagen deposition were restored by surgical resection of the intrauterine adhesions and fibrotic tissue (Fig. 8g).

Increased numbers of CD206^−^ (proinflammatory) HLA-DR^+^ monocytes or macrophages clustering next to the glandular epithelium were also seen in Asherman’s syndrome (Fig. 8h). Although similar clustering was observed in the early secretory phase of the healthy uterus (Supplementary Fig. 5a,b), this was not seen in the healthy proliferative phase. Unlike the restoration of tissue structure, the bias toward inflammatory monocytes or macrophages and their localization with the glandular epithelium was still present after surgery (Fig. 8h–j), suggesting that inflammation induced by these cells may account for the recurrence of adhesions or fibrosis, which occurs in nearly all patients.

As in the healthy human uterus, HLA-DR^lo^ and HLA-DR^hi^ subsets of monocytes together with Mac1 or Mac2 macrophages could be seen in the Asherman’s syndrome uterus using flow cytometry (Fig. 8k). However, there was an increase in monocytes in Asherman’s syndrome (Fig. 8l) which was most striking in the proinflammatory HLA-DR^lo^ subset (Fig. 8m,n). There was a substantial increase in inflammatory Mac1 macrophages that were most closely related to monocytes (Fig. 8o), whereas numbers of Mac2 macrophages remained stable (Fig. 8p). These high numbers of monocytes or Mac1 macrophages persisted after surgery (Fig. 8l–o). There were sustained high numbers of monocytes in the endometrium of patients with Asherman’s syndrome regardless of cycle stage (Fig. 8q), but the increase in Mac1 macrophages was seen only in the proliferative phase (Fig. 8r). Together these data reveal high levels of monocyte or macrophage dysfunction in human endometrial fibrosis that are not restored with surgical treatment.

## Discussion

There is a paucity of information about the functions of myeloid cells in the uterus. Here we addressed this by demonstrating that monocytes play a key role in regulating uterine tissue homeostasis, via combining both inflammatory properties and tissue-remodeling features that are carefully controlled throughout reproductive cycles. Abnormalities of these processes cause dysregulation of the ECM and epithelium, leading to structural changes that contribute to reproductive failure, which in all forms currently has limited treatment strategies.

Single-cell studies infer that CD206^−^ inflammatory macrophages are recruited from the bloodstream with CD206^+^ lipid-associated macrophages being embryonically derived^12^. However, our mouse models show that both CD206^−^ and CD206^+^ macrophages are replenished from the bloodstream, with CD206^+^ (Mac2) macrophages turning over more slowly and thus exhibiting more features of tissue residency. Two subsets of monocytes identified previously^3,14,15^ were proposed to correspond to CD14^+^ classic and CD16^+^ nonclassic monocytes from the bloodstream^14^. We showed that endometrial monocytes can be distinguished based on levels of expression of HLA-DR protein, with both subsets low for CD16. The HLA-DR^lo^ subset of monocytes is potently inflammatory, accounting for the dynamic fluctuations in monocytes observed through the menstrual cycle, thus likely representing recently recruited monocytes.

Our study fills gaps in knowledge about monocyte functions and differentiation of uterine macrophage subsets. Our results suggest that TGFβ regulates uterine macrophage differentiation, corresponding to intestinal macrophages^51^, but also that TGFβ is required for the differentiation and proliferation of Mac2 macrophages. Obvious sources of IFNγ, required to maintain numbers of proinflammatory Mac1 macrophages, include uterine CD8^+^ T cells and natural killer cells. Monocyte infiltration in the early secretory phase of the menstrual cycle conflicts with reports of monocytes identified by single-cell analyses where there are no differences between cycle stages in healthy individuals^13^. However, this study identified cells as monocytes based on expression of molecules such as *S100A8, S100A9* and *IL1B* which are also expressed by inflammatory (recently recruited; Mac1) macrophages.

We showed that inflammatory monocytes or macrophages in humans are localized to the glandular epithelium, with increased numbers during the early secretory phase of the menstrual cycle, followed by a marked reduction in glandular epithelial proliferation in the mid secretory and late secretory phases. These data support our mouse results directly linking monocytes to epithelial cell apoptosis. Although the mechanisms for monocyte regulation of epithelium are unclear, we showed that infiltrating monocytes (HLA-DR^lo^ monocytes in humans) constitutively produced TNF, which can induce apoptosis in glandular epithelial cells^52,53^. Monocytes and Mac1 macrophages in both species expressed the cytotoxic mediators perforin and granzymes, with cell killing representing the top pathway identified by RNA-seq analysis of mouse and human uterine monocytes or macrophages. Although not usually associated with monocytes or macrophages, a cytotoxic phenotype was first described for monocytes nearly 50 years ago^54^ and was found subsequently in responses to tumor cells^55^ and IFNγ^56^, during toxoplasmosis^57^, kidney graft rejection^58^ and via activation by toll-like receptor B signaling^59^.

Epithelial cell apoptosis may be of greater importance in mice, which remodel the endometrium rather than shedding it during menstruation as in humans. In mice, stromal decidualization, which generates the window of implantation, occurs only in noncycling mice after induction by fertilized oocytes on days 4–5 of pregnancy. In humans, menstruation occurs after the removal of decidualized endometrium if there is no pregnancy, as part of the menstrual cycle^60^. Thus in humans, infiltrating monocytes may have more physiological relevance in generating an inflammatory milieu that has been proposed as being required to generate an optimal window of implantation^9,10^. Despite these striking physiological differences, many aspects of monocyte recruitment and differentiation remain conserved between mice and humans.

Candidates potentially involved in recruitment of monocytes to the uterus include Mac2 macrophages, which potently express *CCL2* in mice and humans, and activated fibroblasts, with predicted monocyte interactions involving chemokine receptor–ligand signaling. The cyclical fluctuations in monocytes implicate hormonal involvement either directly or indirectly. The latter is more likely because, although monocytes or macrophages express estrogen (*ESR1*) and progesterone receptors, levels were much higher on stromal cells (Extended Data Fig. 10c–e). An obvious mechanism would be the induction of CCL2 on stromal cells by estrogen^61^. The excess collagen production in the uterus of *Ccr2*^*−/−*^ mice links monocytes and ECM, although it is unclear whether this is due to direct interactions of monocytes and ECM-producing fibroblasts. The functional importance of these pathways is evidenced by breeding abnormalities in *Ccr2*^*−/−*^ mice and the excessively high, sustained numbers of epithelium-associated inflammatory monocytes in human endometrial fibrosis (Asherman’s syndrome). Thus, tissue pathology may occur when the normal dynamic interplay of monocytes, ECM and epithelial cells breaks down, leading to impaired clearance of collagen and the development of fibrosis. The persistence of monocyte or macrophage dysfunction after surgical clearance of fibrotic tissue and removal of collagen deposits may contribute to the propensity of adhesions to re-form in Asherman’s syndrome. Our data support previous studies showing increased proinflammatory macrophages in Asherman’s syndrome^62^ and indicate that myeloid cells could be targeted to improve recovery and prevent re-formation of adhesions after surgery.

Overall, these data reveal monocytes as fundamentally important cells in the uterus regulating tissue homeostasis in a multitude of different ways, including inflammation, tissue remodeling and differentiation into macrophage subsets. These data reveal the importance of kinetics through reproductive cycles and the changing dynamics of immune–structural cell crosstalk, but these pathways must be finely tuned and tightly regulated to avoid reproductive disease. Understanding these processes is critical to generate more targeted, specific treatments in reproductive diseases for which there is an urgent unmet need.

## Methods

### Ethical regulations

This research complies with all relevant ethical regulations.

Mouse work in the UK was approved by the University of Manchester Animal Welfare Ethical Review Board and performed under licenses issued by the UK Home Office (project license (PPL) nos. P70/8548 (Allen) and PP8070044 (Travis)). All experiments were performed in accordance with the UK Home Office regulations.

For mouse work involving work performed at Institut Pasteur (France), animal experiments were conducted in accordance with approval of protocol no. 2016-0010 by the Comité d’éthique en expérimentation animale Paris Centre et Sud and the Comité d’Ethique pour l’Expérimentation Animale Institut Pasteur (the ethics committee for animal experimentation), in application of the European Directive 2010/63 EU.

For human work (UK) samples were obtained according to the principles expressed in the Declaration of Helsinki and under local ethical guidelines, and approved by the North of Scotland Research Ethics Service (reference no. 19/NS/0163) and the North-West -Preston Research Ethics Committee (reference no. 21/NW/0195).

### Mice

C57BL/6J (Charles River, strain no. 000664) mice were housed and maintained at the University of Manchester, UK. *Ccr2*^−/−^ mice (C57BL/6 background) originally from the Jackson Laboratory (strain no. 004999^63^) were maintained at the University of Manchester or gifted from the University of Glasgow (G. Graham). Congenic CD45.1 mice (C57BL/6 background, strain no. 002014^64^) were maintained at the University of Manchester. *Ifngar1*^*−/−*^ mice (C57BL/6 background, gifted from W. Müller^65^) were maintained at the University of Manchester. All mice were housed in individually ventilated cages under specific pathogen-free conditions at the University of Manchester, UK. The mice were held in a 12:12 light-to-dark cycle, at 21 °C ± 2 °C, relative humidity 40–60%. Mice had free access to standard pelleted rodent chow and fresh water, with basic bedding and nesting material. Chow constituents included: moisture 12%, crude protein 18.5%, crude oils and fats 5.50%, crude fibers 4.50% and crude ash 6%. For all experiments other than those involving older mice, mice were aged between 9 weeks and 12 weeks. For experiments involving older mice, mice were aged 9–12 months.

### Human samples

Human endometrial biopsies were obtained from patients undergoing colposcopy, hysteroscopy or surgery at St Mary’s Hospital, Manchester, UK. Samples were taken from healthy controls undergoing human papillomavirus screening or through reproductive clinics from individuals not able to conceive due to male factor infertility or unexplained subfertility. In some cases healthy controls consented to procedures for research purposes only. All individuals included for the hysteroscopy had findings of a normal endometrium and all healthy controls had no reproductive diseases (for example, recurrent miscarriage or recurrent implantation failure), no inflammatory or structural disorders of the uterus (for example, endometriosis, Asherman’s syndrome, fibroids or adenomyosis) and no heavy menstrual bleeding. Samples from patients with Asherman’s syndrome (endometrial fibrosis) were taken at the start of adhesiolysis surgery or during hysteroscopy pre-surgery or post-surgery. Pre-surgery samples were taken during the proliferative phase of the menstrual cycle to allow direct comparison with the post-surgery cohort who had all been given long-acting estrogen to induce endometrial proliferation, thus mimicking the proliferative phase of the cycle. All patients provided written, informed consent for the collection of tissue samples and subsequent analysis. Patient diagnosis was confirmed by established clinical and histological criteria. Patient demographics are summarized in Supplementary Table 1. Ethnicity subcategories were defined by the researchers (white, black, Asian, mixed race) with the ethnic identity of participants determined by standard National Health Service reporting systems (defined by participants; Supplementary Table 1). No financial compensation was provided.

### Reproductive cycle staging

In mice, the stages of the estrus cycle were assessed at the time of tissue harvest, based on the proportions of cell types observed in vaginal secretions, as previously described^66^. In humans, patients undergoing colposcopy or hysteroscopy were characterized as being in proliferative or early secretory, midsecretory or late-secretory phases of the menstrual cycle, based on their usual cycle length, date of their last menstrual period and the date of the procedure. Ovulation was estimated at 14 d before the start of their next predicted menstrual period, with samples taken after this date classified as secretory phase (early: days 1–4 of the secretory phase; mid: days 5–9 of the secretory phase; and late: days 10–14 of the secretory phase) and samples taken before ovulation as the proliferative phase.

### Murine uterus digest

Both uterine horns were removed from mice and collected in Roswell Park Memorial Institute-1640 medium (Thermo Fisher Scientific), minced using fine scissors and incubated for 40 min in 1.25 mg ml^−1^ of collagenase D (Sigma-Aldrich), 0.85 mg ml^−1^ of collagenase V (Sigma-Aldrich) and 1 mg ml^−1^ of dispase (Gibco, Invitrogen) in a shaking incubator at 37 °C. Tissue was then filtered through a 100-μm filter and cells were analyzed by flow cytometry.

### Human endometrial biopsy digest

Endometrial Pipelle biopsies were collected in 5 ml of PBS and centrifuged to separate tissue from PBS supernatant, which was then removed. Tissue pellets were then resuspended in 0.5 mg ml^−1^ of collagenase VIII (Sigma-Aldrich), 0.65 mg ml^−1^ of collagenase D (Sigma-Aldrich), 1 mg ml^−1^ of dispase and 0.015 mg ml^−1^ of DNase (Sigma-Aldrich) and incubated for 35 min in a shaking incubator at 37 °C. After incubation, tissue was filtered through a 100-μm filter and tissue suspensions were pooled after digests. Cells were washed and red blood cell lysis was carried out using double distilled water for 10 s followed immediately by 1/10th the volume of 10× PBS. Cells were then analyzed by flow cytometry or used for in vitro stimulation assays.

### Flow cytometry

A total of 0.2–2.0 × 10^6^ cells were stained at 4 °C in the dark, in PBS with 2% fetal calf serum and 2% EDTA (FACS buffer) with Fc block (BioLegend) for 20 min. The antibodies used are listed in Supplementary Tables 2a (mice) and 2b (human). For surface staining, cells were fixed in 2% paraformaldehyde (Sigma-Aldrich) for 30 min, washed and resuspended in FACS buffer before acquisition. For intracellular staining, after incubation with antibodies for surface staining, cells were washed in FACS buffer, then fixed and permeabilized (eBioscience intracellular staining kit) before incubation for 60 min with antibodies for intracellular proteins (room temperature). Cells were washed and resuspended in perm buffer (eBioscience) before acquisition. Cells were analyzed using an LSR Fortessa cytometer (BD Biosciences) and FlowJo software (v10, Treestar). In all cases, cell populations and surface or intracellular molecules were gated based on reference isotype-matched control samples. For lipopolysaccharide (LPS) stimulation experiments, isotype-matched control staining was carried out on stimulated cells for reference gating. Absolute numbers of cell subsets were calculated per uterus in mice and per mg of tissue in humans. The antibodies are listed in Supplementary Table 2a,b.

### RNA-seq

Murine scRNA-seq methods and any associated references are available in Supplementary Note 1. Human bulk RNA-seq methods and any associated references are available in Supplementary Note 2.

### CellChat analysis

CellChat (v2.1.0) was utilized to predict the potential cell–cell communication patterns between cell populations of interest. The algorithm calculated the probability of the ligand and its matching receptor expression on the cells of interest, as well as *P* values <0.05 which indicated a significant interaction. The communication strength and number of cell–cell interactions were then displayed through several bubble, circle and chord diagrams. Heatmaps were utilized to demonstrate the specific roles of cell populations in many signaling pathways and their involvement as senders, receivers, mediators or regulators.

### Histology processing

Fragments of the uterine horns were fixed in 4% formalin for 1 h and embedded in paraffin wax blocks. Tissues were cut into 5-μm sections using a microtome and placed on to superadhesive microscope slides.

### Antibody-mediated TGFβ blockade

TGFβ activity was blocked by injecting C57BL/6J (Charles River, JAX) mice i.p. with 0.5 mg of anti-TGFβ-blocking antibody (clone no. 1d11.16.8, BioXCell) every other day for 2 weeks, as previously described^67^. Similarly, a cohort of C57BL/6J mice were i.p. injected with control immunoglobulin G1 antibody every other day for 2 weeks.

### Hydroxyproline assay

Hydroxyproline content was measured in paraffin-embedded uterine tissue, as previously described^68^. Using the QuickZyme Biosciences Total Collagen Assay and Total Protein Assay kits, uterine tissue was hydrolyzed in HCl at 95 °C for 20 h as per the manufacturer’s instructions. Hydroxyproline content was determined by absorbance at 570 nm against standard kit controls and expressed per total amount of protein assessed in the sample.

### Immunohistochemistry

Formalin-fixed paraffin-embedded (FFPE) sections were deparaffinized and incubated with hematoxylin, followed by eosin to observe the architecture of the tissue. Picrosirius Red and Fast Green were utilized to stain collagen fibers within the FFPE tissue.

### Immunofluorescence and analysis

Deparaffinized FFPE sections were treated with sodium citrate, pH 6.0, to retrieve antigens. Sections were blocked for 1 h with 3% bovine serum albumin (BSA) and 0.1% Triton X-100 and goat serum (1:20) or donkey serum (1:20) in PBS. Immunostaining was performed using F4/80, Ly6c, CD64 and ClC3 in staining buffer (0.5% BSA in PBS) overnight. Sections were washed and stained with goat anti-mouse Alexa Fluor-488, goat anti-rat Alexa Fluor-555 and donkey anti-rabbit Alexa Fluor-647 for 1 h at room temperature. The sections were washed and stained with DAPI. Stained sections were scanned using the three-dimensional Histech Pannoramic P250 Slide Scanner or at least three random images of each section were taken using the Zeiss Axioscope 5FL-LED Fluorescence Microscope.

QuPath (v0.7.0) and ImageJ (v1.54t) were utilized for image analysis and configuration. For collagen quantification, a pixel-identifying model was trained by distinguishing collagen-positive from collagen-negative areas such as epithelial glands across several ROIs and several tissues. The area of collagen was then measured per square micrometer, in five ROIs of size 0.5 μm × 0.5 μm per tissue section.

### Hyperion imaging mass cytometry

Metal isotypes were conjugated to BSA and azide-free antibodies through chelating polymers within the MaxPar Multimetal Labelling Kit (Standard BioTools). The final concentrations of the conjugated antibodies were measured using Nanodrop (Thermo Fisher Scientific) and the antibodies were reconstituted with Antibody Stabilisation Solution (Candor Bioscience) and, then, stored at 4 °C. The antibodies used are listed in Supplementary Table 3.

Deparaffinized FFPE sections were treated with Tris-EDTA, pH 9.0, to retrieve antigens and subsequently blocked with 3% BSA in PBS for 1 h at room temperature. A cocktail of metal-conjugated antibodies (Supplementary Table 2) was prepared in PBS with 0.3% BSA and added to the FFPE sections that were left overnight at 4 °C. FFPE sections were supplemented with a metal-conjugated streptavidin, followed by Cell-ID Intercalator-Iridium (1:400 in PBS, Standard BioTools) incubation for 30 min at room temperature. Slides were washed with deionized water (Merck Millipore) and dried overnight before data acquisition.

Raw imaging mass cytometry (IMC) data were acquired using a Hyperion imaging mass cytometer along with a Helios time-of-flight mass cytometer (CyTOF, Standard BioTools). ROIs were selected at 750 × 750 μm^2^ and laser ablated at 1-μm^2^ pixel resolution and a frequency of 200 Hz. Images of each antibody channel were exported from the raw IMC data as TIFF files using either MCD Viewer or HistoCat (Standard BioTools).

### Hyperion IMC preprocessing, segmentation and analyses

IMC data were analyzed using the open-source imcanalysis workflow, with all analysis code publicly available on GitHub (https://github.com/dr-michael-haley/imcanalysis). Raw IMC acquisitions were converted into per-region marker images and metadata tables, followed by denoising with IMC-Denoise^69^ which removes IMC-specific technical noise, including hot-pixel artifacts and ion-counting shot noise. Cells were segmented with CellPose-SAM^70^ using the nuclear iridium stain and a conservative 1-µm mask expansion was applied to capture adjacent cytoplasm^71^; these expanded masks were then used for downstream single-cell quantification. Marker expression was then quantified using Nimbus-Inference (https://github.com/angelolab/Nimbus-Inference and https://www.nature.com/articles/s41592-025-02826-9), which classifies marker positivity from image context. To integrate samples across experimental batches, BioBatchNet (https://github.com/UoM-HealthAI/BioBatchNet and https://www.biorxiv.org/content/10.1101/2025.03.15.643447v1) was used for batch-effect correction, aiming to reduce technical batch variation while preserving biological differences between cell populations. Corrected single-cell data were then used for unsupervised clustering using Leiden’s algorithm within scanpy (https://github.com/scverse/scanpy) to define cell populations. Populations were annotated using canonical phenotypic marker-expression patterns and established tissue biology. Annotation quality was validated by backgating each population into the original tissue context to confirm expected spatial localization.

Images from raw IMC files were pre-processed using steinbock v0.16.3^72^ and individual cells were segmented using a machine learning algorithm called mesmer by deepcell^73^. Intensity and regionprop values were exported for each cell and compiled together into an Anndata file for further downstream analyses. The mean cell intensity of each marker was normalized to the 99th percentile of its expression. After UMAP and principal component analysis of the dataset, Leiden-based clustering at 0.3 resolution was utilized to identify general populations of immune and stromal cells, which were further subclustered and manually identified by their expression of canonical cell markers for each cell type. Through Python packages such as squidpy (v1.2.3) and scanpy (v1.9.3) in the IMC analysis v2 tutorials^74^, cells were quantified and compared between different cycle stages and conditions.

Cellcharter (v0.3.5) was utilized to determine the proximity between clusters of cells to other cells in tissue niches^75^. To quantify spatial distances between cell populations, we computed *k*-nearest neighbors distances per ROI using a permutation bootstrap. For each cell, we measured distance to the nearest cell of each population, then compared observed values with ROI-matched null distributions generated by shuffling population labels (preserving coordinates and composition). We reported observed mean distance, randomized mean distance, *δ* (observed − randomized) and *z*-score (*δ* or randomized s.d.); negative values indicate closer-than-expected proximity.

Cell–cell interaction enrichment was also assessed with Squidpy^76^ using a 20-µm neighborhood threshold (<20 µm between cells). The *z*-scores were calculated against complete spatial randomness using 1,000 within-region label permutations; positive scores indicate enriched interactions and negative scores depleted interactions.

### Uterus-shielded BM chimeras

For shielded irradiation, 8-week-old to 10-week-old WT female CD45.1 C57BL/6 mice were anesthetized by administration of ketamine (80 mg kg^−1^; Vetoquinol) and xylazine (8 mg kg^−1^; Bayer) i.p. Mice were irradiated with their abdomens positioned beneath lead shielding and received 11 Gy of irradiation as a split dose. Mice were reconstituted by intravenous injection with (5–10) × 10^6^ donor BM cells from CD45.2 (WT or Ccr2^−/−^) congenic mice. Mice were maintained on 0.03% enrofloxacin (Bayer) supplied through drinking water 1 week before and 4 weeks after irradiation. After irradiation, mice were housed in autoclaved cages with sterile drinking water, food, bedding and enrichment. Reconstitution was allowed to occur for a minimum of 8 weeks before analysis. For experiments involving reconstitution of mice with WT BM only (University of Manchester), chimerism of uterine and colonic macrophages was normalized to that of circulating monocytes. For experiments involving comparison of reconstitution of mice with Ccr2^+/+^ and Ccr2^−/−^ BM (Institut Pasteur), chimerism of uterine macrophages was normalized to that of circulating eosinophils, because this was the only circulating immune cell population that demonstrated comparable levels of chimerism in the blood of mice reconstituted with Ccr2^+/+^ or Ccr2^−/−^ BM (Supplementary Figure 3).

### Cytokine production in response to LPS

After tissue digest, murine uterine or human endometrial cells were incubated for 3 h with monensin and brefeldin A (Sigma-Aldrich) in the presence or absence of 100 ng ml^−1^ of LPS (Sigma-Aldrich). Monocytes and macrophages were then identified by flow cytometry and assessed for intracellular cytokine production after cell permeabilization and fixation (eBioscience intracellular staining kit).

### Statistical analysis

Statistical analyses were performed using GraphPad Prism v10 for Mac. Statistical differences were tested and described in the figure legends. Normality tests were performed on all datasets. Groups were compared using a two-tailed, unpaired Student’s *t*-test (normal distribution) or a Mann–Whitney *U*-test (failing normality testing) for comparison of two groups. The two-tailed, paired Student’s *t*-test (normal distribution) or Wilcoxon’s matched-pair, signed-rank test (failing normality testing) was used to compare results (for example, from different cells or tissues) from the same individual. A two-tailed ANOVA with Tukey’s post hoc testing (normal distribution) or a Kruskal–Wallis test with Dunn’s post hoc testing (failing normality testing) was used for multiple-group comparisons. In all cases, data are shown as the mean ± s.e.m. for parametric data and the median ± IQR for nonparametric data. In all cases, *P* ≤ 0.05 was considered statistically significant. ^*^P < 0.05, ^**^P < 0.01, ^***^P < 0.001, ^****^P < 0.0001.

### Reporting summary

Further information on research design is available in the Nature Portfolio Reporting Summary linked to this article.

## Online content

Any methods, additional references, Nature Portfolio reporting summaries, source data, extended data, supplementary information, acknowledgements, peer review information; details of author contributions and competing interests; and statements of data and code availability are available at https://doi.org/10.1038/s41590-026-02651-y.

## Supplementary information

Supplementary Information Supplementary Figs 1–5, Tables 1, 2a, 2b and 3 and Notes 1 and 2.

Reporting Summary

Peer Review File

## Source data

Source Data all Figs. and Extended Data Figs. Statistical source data.

## Data Availability

Raw sequencing data have been deposited in the ArrayExpress database at EMBL-EBI under the following accession nos. for RNA-seq (human) (E-MTAB-15397) and scRNA-seq (murine) (E-MTAB-15391). Source data are provided with this paper.
