# Supplementary material for: The dual roles of uterine monocytes in regulation of tissue homeostasis throughout reproductive cycles in health and disease
Source: Nat Immunol. 2026 Sep 8;27(10):2058–73. doi: 10.1038/s41590-026-02651-y (PMC13630565; doi:10.1038/s41590-026-02651-y)
Supplement: Supplementary file 1 [file 41590_2026_2651_MOESM1_ESM.pdf]

nature immunology

Supplementary information https://doi.org/10.1038/s41590-026-02651-y

The dual roles of uterine monocytes in regulation of tissue homeostasis throughout reproductive cycles in health and disease

In the format provided by the authors and unedited

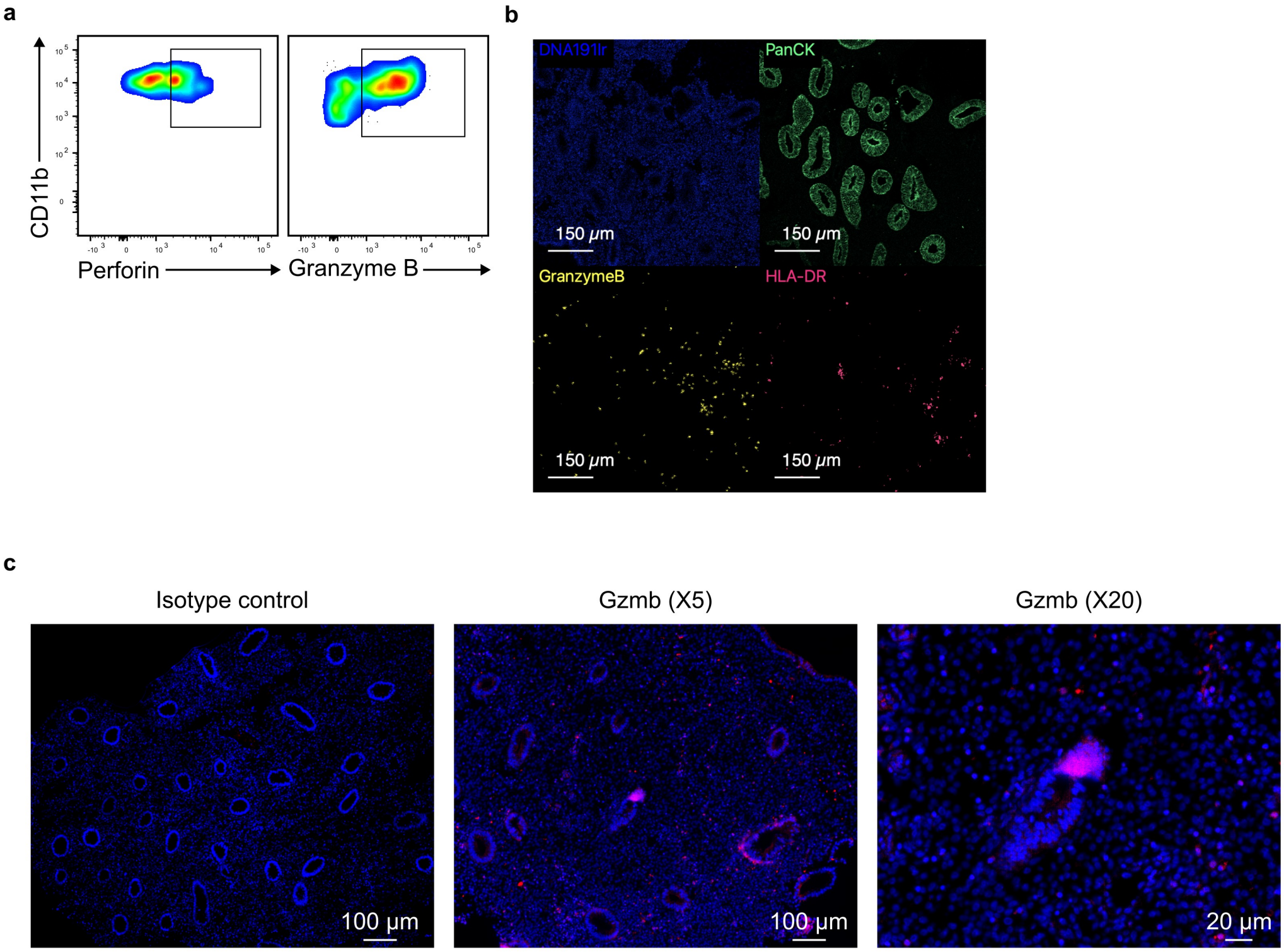
a
CD11b
Perforin
Granzyme B
b
DNA191Ir
PanCK
GranzymeB
HLA-DR
150 μm
c
Isotype control
Gzmb (X5)
Gzmb (X20)
100 μm
20 μm

Supplementary figure 1. Expression of perforin and granzyme in human monocytes and macrophages, and collagen in *Ccr2*$^{-/-}$ mice. A) Flow cytometry plot showing example gating for perforin and granzyme B protein expression by human monocytes, pre-gated on CD45^{+}live Sig8^{-}CD66b^{-}CD3^{-}CD19^{-}CD56^{-}HLA-DR^{+}CD64^{+}CD14^{+}CCR2^{+} cells. **B)** Imaging mass cytometry pictures demonstrating individual panels for DNA, pancytokeratin, granzyme B and HLA-DR within the human endometrium. **C)** Immunofluorescence microscopy images demonstrating granzyme B expression with the same antibody on alternative human endometrial sections. Images are representative of several independent experiments with similar results.

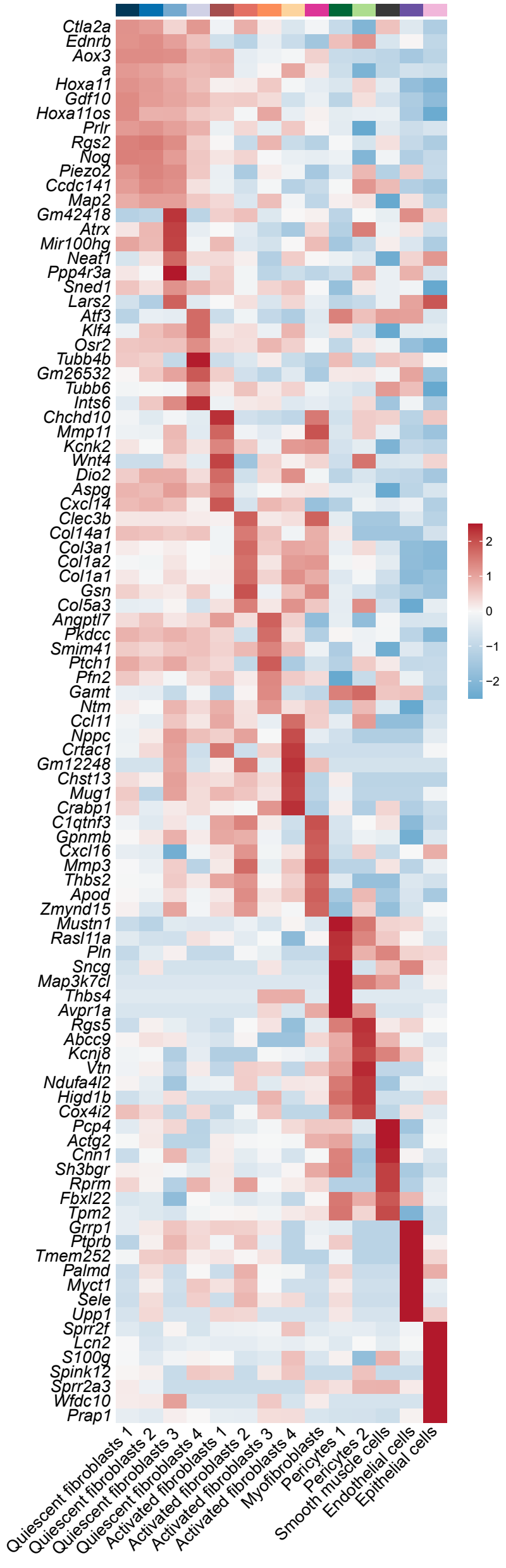

Ctla2a
Ednrb
Aox3
a
Hoxa11
Gdf10
Hoxa11os
Prlr
Rgs2
Nog
Piezo2
Ccdc141
Map2
Gm42418
Atrx
Mir100hg
Neat1
Ppp4r3a
Sned1
Lars2
Atf3
Klf4
Osr2
Tubb4b
Gm26532
Tubb6
Ints6
Chchd10
Mmp11
Kcnk2
Wnt4
Dio2
Aspg
Cxcl14
Clec3b
Col14a1
Col3a1
Col1a2
Col1a1
Gsn
Col5a3
Angptl7
Pkdcc
Smim41
Ptch1
Pfn2
Gamt
Ntm
Ccl11
Nppc
Crtac1
Gm12248
Chst13
Mug1
Crabp1
C1qtnf3
Gpnmb
Cxcl16
Mmp3
Thbs2
Apod
Zmynd15
Mustn1
Rasl11a
Pln
Sncg
Map3k7cl
Thbs4
Avpr1a
Rgs5
Abcc9
Kcnj8
Vtn
Ndufa4l2
Higd1b
Cox4i2
Pcp4
Actg2
Cnn1
Sh3bgr
Rprm
Fbxl22
Tpm2
Grrp1
Ptprb
Tmem252
Palmd
Myct1
Sele
Upp1
Sprr2f
Lcn2
S100g
Spink12
Sprr2a3
Wfdc10
Prap1
2
1
0
−1
−2
Quiescent fibroblasts 1
Quiescent fibroblasts 2
Quiescent fibroblasts 3
Quiescent fibroblasts 4
Activated fibroblasts 1
Activated fibroblasts 2
Activated fibroblasts 3
Activated fibroblasts 4
Myofibroblasts
Pericytes 1
Pericytes 2
Smooth muscle cells
Endothelial cells
Epithelial cells

Supplementary figure 2. Heatmap of top cluster-defining genes for CD45$^-$ cell populations identified in mouse single cell RNAseq analysis. Heatmap representing cell cluster average gene expression of the top seven genes in each cell cluster ranked by log2 fold change relative to other clusters.

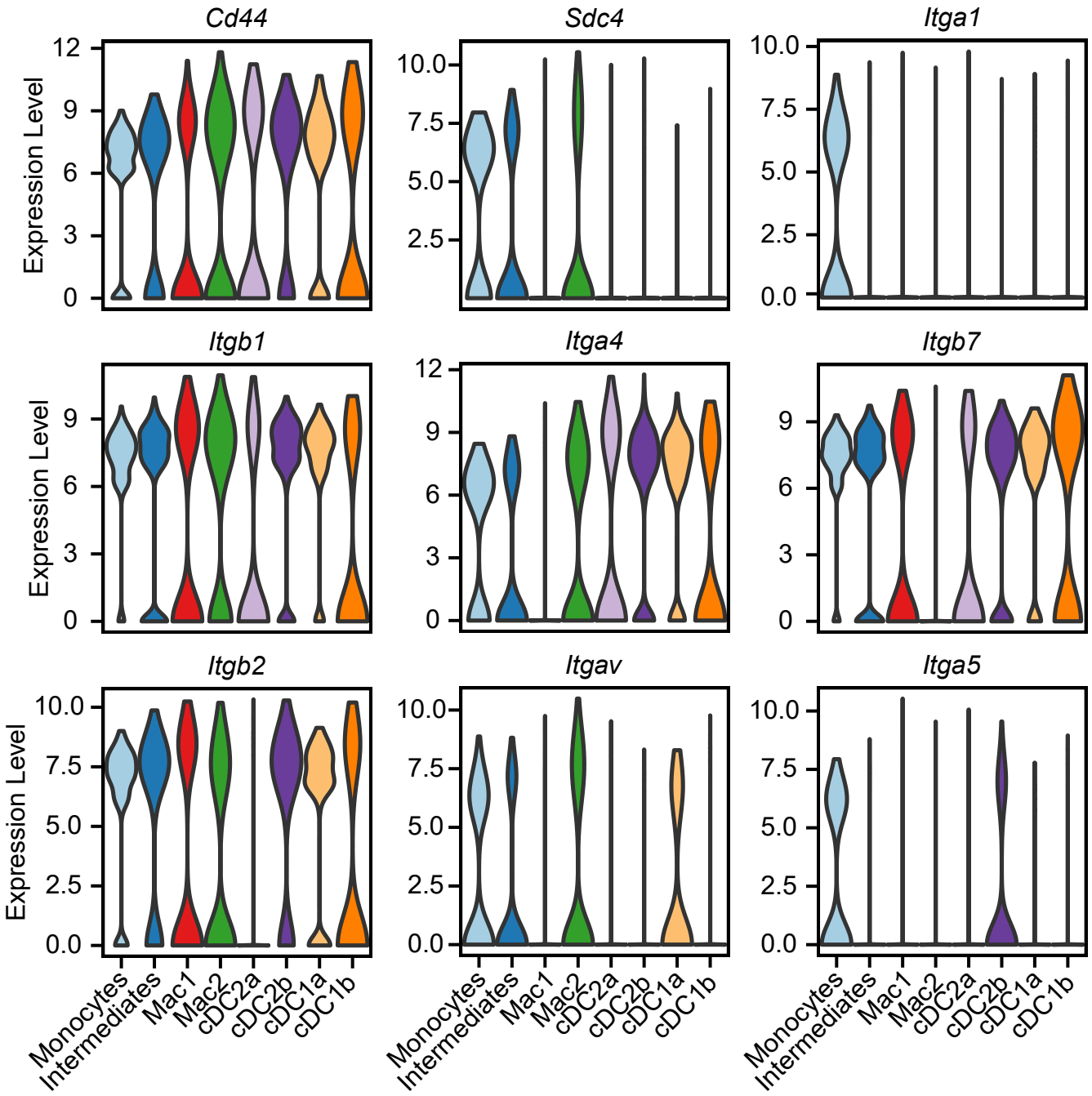

Cd44
Sdc4
Itga1
Itgb1
Itga4
Itgb7
Itgb2
Itgav
Itga5
Expression Level
Monocytes
Intermediates
Mac1
Mac2
cDC2a
cDC2b
cDC1a
cDC1b

Supplementary figure 3. Expression of molecules involved in binding ECM-producing cells in mouse single cell RNAseq analysis of myeloid cells. Violin plots demonstrate the distribution of expression of selected genes by myeloid cell subsets, identified as being able to bind ECM producing $CD45^{-}$ cell subsets.

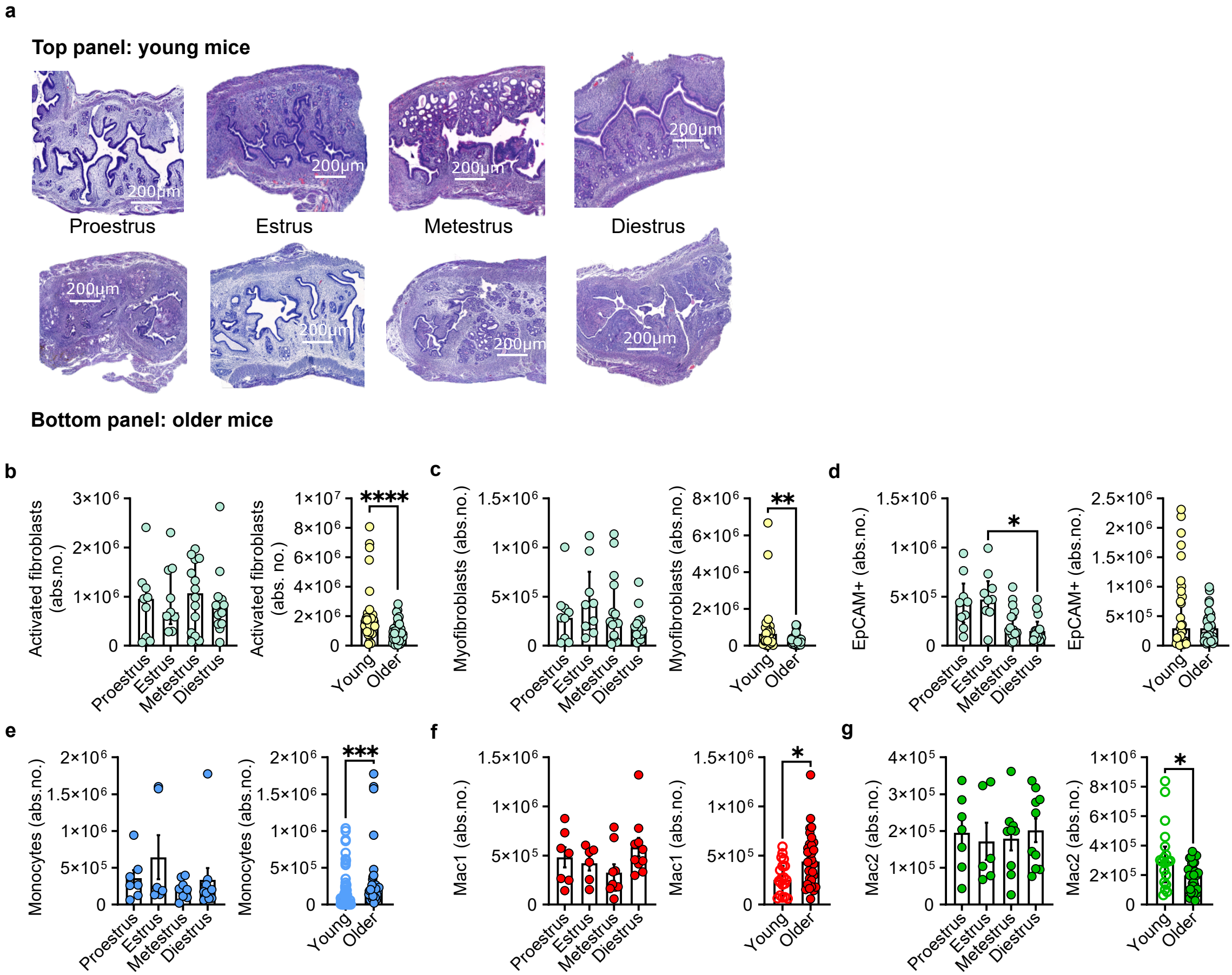
a
Top panel: young mice
200µm
Proestrus
Estrus
Metestrus
Diestrus
Bottom panel: older mice
b
Activated fibroblasts (abs.no.)
Activated fibroblasts (abs. no.)
****
c
Myofibroblasts (abs.no.)
**
d
EpCAM+ (abs.no.)
*
e
Monocytes (abs.no.)
***
f
Mac1 (abs.no.)
*
g
Mac2 (abs.no.)
*
Young
Older

Supplementary figure 4. Uterine histology in older mice and absolute numbers. A) H&E staining of cross sections of murine uterine horn in young (<16 weeks) and older (9-12 months) mice taken during proestrus, estrus, metestrus and diestrus. Images are representative of several independent experiments with similar results. **B)** Summary graph of flow cytometric analyses of absolute numbers (per uterus) of activated fibroblasts ($CD45^{-}$ live $EpCAM^{-}CD31^{-}MCAM^{-}PDGFR\alpha^{+}CD90^{+}$ cells) in older mice at individual estrous cycle stages (proestrus n=9, estrus n=9, metestrus n=14, diestrus n=13) and total activated fibroblasts in young (n=37) and older mice (n=46), data pooled across reproductive cycle stages. Two-tailed Mann-Whitney test was applied. **C)** Summary graph of flow cytometric analyses of absolute numbers (per uterus) of myofibroblasts ($CD45^{-}$ live $EpCAM^{-}CD31^{-}MCAM^{-}PDGFR\alpha^{+}CD90^{-}$ cells) in older mice at individual estrous cycle stages (proestrus n=9, estrus n=9, metestrus n=14, diestrus n=13) and total myofibroblasts in young (n=37) and older mice (n=46), data pooled across reproductive cycle stages. Two-tailed Mann-Whitney test was applied. **D)** Summary graph of flow cytometric analyses of absolute numbers (per uterus) of epithelial cells ($CD45^{-}$ live $EpCAM^{+}CD31^{-}$ cells) in older mice at individual estrous cycle stages (proestrus n=9, estrus n=9, metestrus n=14, diestrus n=13; Kruskal-Wallis test) and total epithelial cells in young (n=37) and older mice (n=46), data pooled across reproductive cycle stages. **E)** Summary graph of flow cytometric analyses (per uterus) of absolute numbers of monocytes ($CD45^{+}$ live $Ly6G^{-}SigF^{-}CD11b^{+}CD64^{+}Ly6C^{+}$ cells) in older mice at individual estrous cycle stages (proestrus n=8, estrus n=6, metestrus n=9, diestrus n=10) and total monocytes in young (n=54) and older mice (n=33), data pooled across reproductive cycle stages. Two-tailed Mann-Whitney test was applied. **F)** Summary graph of flow cytometric analyses (per uterus) of absolute numbers Mac1 macrophages ($CD45^{+}$ live $Ly6G^{-}SigF^{-}$ $CD11b^{+}CD64^{+}Ly6C^{-}CD11c^{+}CD206^{-}$ cells) in older mice at individual estrous cycle stages (proestrus n=8, estrus n=6, metestrus n=9, diestrus n=10) and total Mac1 macrophages in young (n=19) and older mice (n=32), data pooled across reproductive cycle stages. Two-tailed Mann-Whitney test was applied. **G)** Summary graph of flow cytometric analyses (per uterus) of absolute numbers of Mac2 macrophages ($CD45^{+}$ live $Ly6G^{-}SigF^{-}$ $CD11b^{+}CD64^{+}Ly6C^{-}CD11c^{-}CD206^{+}$ cells) in older mice at individual estrous cycle stages (proestrus n=8, estrus n=6, metestrus n=9, diestrus n=10) and total Mac2 macrophages in young (n=19) and older mice (n=33), data pooled across reproductive cycle stages. Two-tailed Mann-Whitney test was applied. In all cases, at least two independent experiments were carried out. For Kruskal-Wallis tests adjusted p values are reported from Dunn's *post hoc* tests respectively. *$p<0.05$, **$p<0.01$, ***$p<0.001$, ****$p<0.0001$. Data are presented as median±IQR in B, C, D, E, F, G.

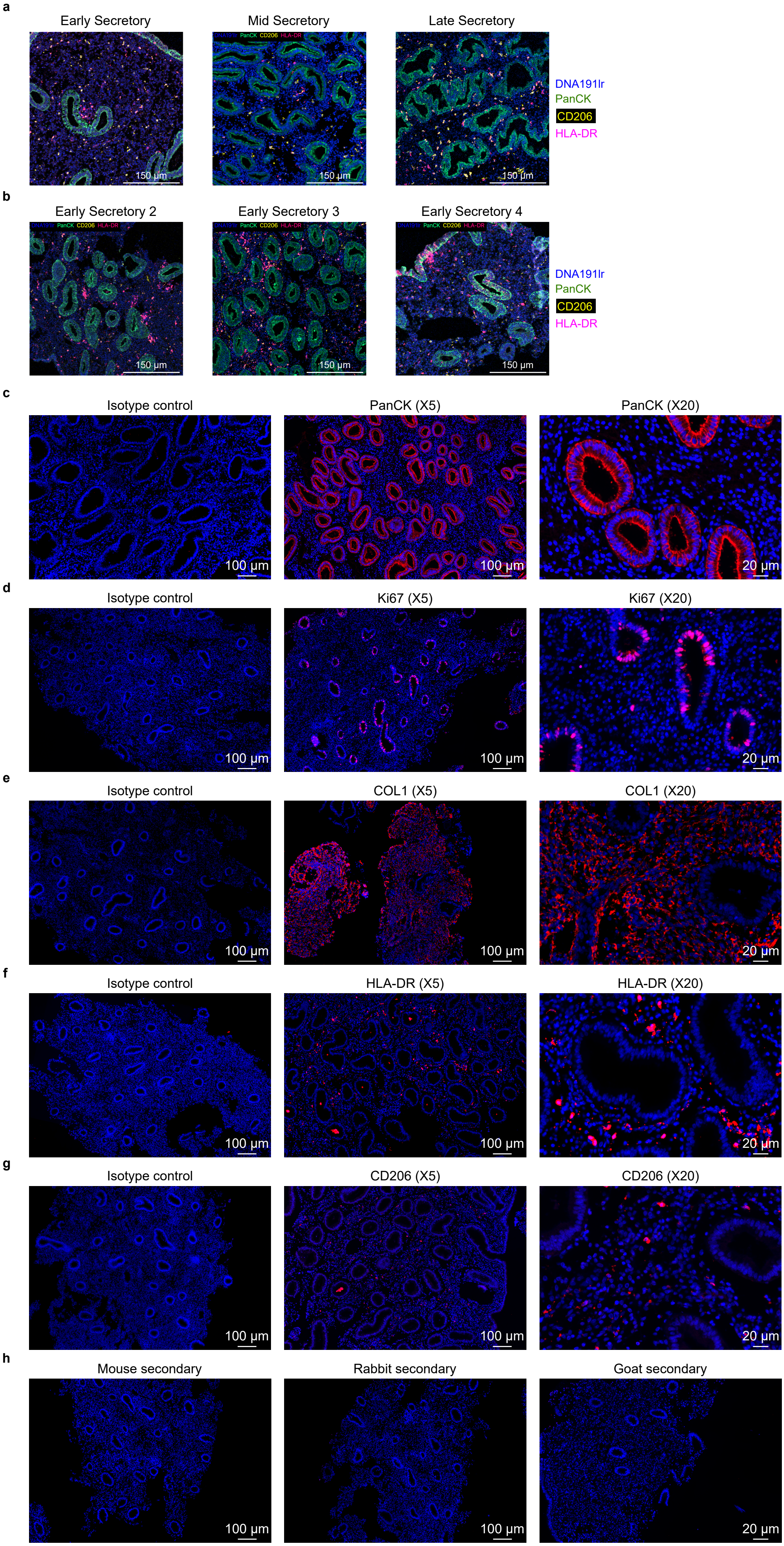
a
Early Secretory
Mid Secretory
Late Secretory
150 µm
DNA191Ir
PanCK
CD206
HLA-DR
b
Early Secretory 2
Early Secretory 3
Early Secretory 4
c
Isotype control
PanCK (X5)
PanCK (X20)
100 µm
20 µm
d
Ki67 (X5)
Ki67 (X20)
e
COL1 (X5)
COL1 (X20)
f
HLA-DR (X5)
HLA-DR (X20)
g
CD206 (X5)
CD206 (X20)
h
Mouse secondary
Rabbit secondary
Goat secondary

Supplementary figure 5. Hyperion and immunofluorescence imaging in the human endometrium. A) Imaging mass cytometry pictures demonstrating pancytokeratin staining in epithelium, alongside inflammatory monocytes/Mac1 macrophages ($CD206^-$ $HLA\text{-}DR^+$ cells) and Mac2 macrophages ($CD206^+HLA\text{-}DR^+$ cells), in sections taken from endometrial biopsies at different phases of the menstrual cycle in healthy controls. Images are representative of 5 different regions of interest (ROI) within one sample, and of 13 samples. **B)** Imaging mass cytometry pictures demonstrating pancytokeratin staining in epithelium, alongside inflammatory monocytes/Mac1 macrophages ($CD206^-$ $HLA\text{-}DR^+$ cells) and Mac2 macrophages ($CD206^+HLA\text{-}DR^+$ cells), in several different samples all taken from endometrial biopsies during the early secretory phase of the menstrual cycle in healthy controls. Images are representative of 5 different regions of interest (ROI) within one sample, and of 4 biological replicates. **C)** Immunofluorescence microscopy images alongside relevant isotype control staining demonstrating pancytokeratin staining, **D)** Ki67 staining, **E)** Col1 staining, **F)** HLA-DR staining and **G)** CD206 staining. **H)** Additional control staining showing secondary staining only and no primary stain. The same antibodies were used for imaging mass cytometry in other experiments.

Supplementary table 1

	Ctrl Proliferative (n=24)	Ctrl Early Sec (n=12)	Ctrl Mid Sec (n=12)	Ctrl Late Sec (n=9)	Asherman Proliferative (pre-surgery; n=6)	Asherman Secretory (pre-surgery; n=6)	Asherman post-surgery (n=7)	p value *<0.05
Age	33±3.70	33±1.23	33±3.96	32±1.34	39±4.51	36±1.32	35±6.616	NS
BMI	28.2±1.29	25.2±0.94	24.8±1.29	24.1±1.29	25.4±1.80	27.8±2.07	23.4±1.243	NS
Ethnicity	White: 16/24 Black: 5/24 Asian: 3/24 Mixed race: 0/24	White: 8/12 Black: 0/12 Asian: 2/12 Mixed race: 2/12	White: 10/12 Black: 1/12 Asian: 0/12 Mixed race: 1/12	White: 7/9 Black: 1/9 Asian: 1/9 Mixed race: 0/9	White: 2/6 Black: 1/6 Asian: 2/6 Mixed race: 1/6	White: 4/6 Black: 1/6 Asian: 1/6 Mixed race: 0/6	White: 7/7 Black: 0/7 Asian: 0/7 Mixed race: 0/7	
Gravidity	2.1±0.45	2.2±0.55	2.0±0.39	1.8±0.43	2.3±1.02	1.8±0.87	2.0±0.787	NS
Live births	1.1±0.22	1.3±0.35	1.3±0.23	1.2±0.32	0.3±0.21	0.3±0.21	0.0±0.00	* (Asherman)

Supplementary table 1.

Patient cohort characteristics. Values are shown as mean±standard error of the mean

Supplementary table 2a

Marker	Fluorophore	Clone	Manufacturer
**CD11c**	BV785	N416	Biolegend
**Ly6c**	PerCP Cy5.5	5HK1.4	Biolegend
**CD64**	APC	X54-5/7.1	Biolegend
**MHC II**	AF700	M5/114.15.2	Biolegend
**Viability dye**	APC Cy7		Invitrogen
**CD206**	BV650	C068C2	Biolegend
**Ly6G**	PerCP Cy5.5	1A8	Biolegend
**Siglec F**	BV650	E20-2440	BD
**Perforin**	FITC	S16009A	Biolegend
**Granzyme B**	BV421	GB11	Biolegend
**Granzyme A**	PE	GzA-3G8.5	Invitrogen
**Granzyme C**	PE	SFC1D8	Biolegend
**CD45**	BV510	30-F11	Biolegend
**CD11b**	BUV395	M1/70	BD
**CCR2**	FITC	SA203G11	Biolegend
**Ki67**	BV421	SolA15	Invitrogen
**LYVE-1**	FITC	ALY7	Invitrogen
**CX3CR1**	BV605	SA011F11	Biolegend
**CD31**	BV785	390	Biolegend
**CD29**	BV421	HMB1-1	Biolegend
**CD34**	AF700	RAM34	Invitrogen
**Podoplanin (GP38)**	PE Dazzle	8.1.1	Biolegend
**EpCAM**	BV650	G8.8	Biolegend
**PDGFRa (CD140a)**	BV605	APA5	Biolegend
**CD9**	FITC	MZ3	Biolegend
**CD106**	PerCP Cy5.5	429 (MVCAM.A)	Biolegend
**CD146**	PE Cy7	ME-9F1	Biolegend
**CD90**	PE	5a-8	Invitrogen
**NK1.1**	PE Cy7	PK136	Biolegend
**NKp46 (CD335)**	PE	29A1.4	Biolegend

Supplementary table 2a.
Flow cytometry antibodies and clones used for staining murine uterine tissue following tissue digest.

Supplementary table 2b

Marker	Fluorophore	Clone	Manufacturer
**CD11c**	AF700	Bu15	Biolegend
**CD14**	PE	M5E2	Biolegend
**CD64**	APC	10.1	Biolegend
**HLA-DR**	BV785	L243	Biolegend
**CD206**	BV650	19.2	BD
**CD117 (c-Kit)**	PerCP Cy5.5	10402	Invitrogen
**FCeRI**	PE Cy7	AER-37 (CRA-1)	Biolegend
**Perforin**	FITC	Dg9	Invitrogen
**Granzyme B**	Texas Red	GRB17	Invitrogen
**PF4 (biotinylated)**	None	170138	Novus Bio
**SPP1**	APC	2F10	Invitrogen
**CD45**	BV510	HI30	Biolegend
**KLRG-1**	FITC	13F12F2	Invitrogen
**CD127**	BV421	A019D5	Biolegend
**CD90**	BV605	5E10	Biolegend
**CD20**	AF700	2H7	Biolegend
**CD19**	AF700	HIB19	Invitrogen
**CCR2**	PE Cy7	K036C2	Biolegend
**NKp46**	BV650	9E2	Biolegend
**NKp44**	PE	P44-8	Biolegend
**CD3**	PE Cy7	UCHT1	Biolegend
**CD5**	PE Cy7	L17F12	Biolegend
**NKG2D**	PE Dazzle	1D11	Biolegend
**Eomesodermin**	APC	X4-83	BD
**CD56**	BV605	NCAM16.2	BD
**CD209**	PerCP Cy5.5	DCN46	BD
**Clec9a**	PE	8F9	Biolegend
**XCR1**	PE Cy7	S15046E	Biolegend
**Axl**	BV421	108724	BD
**EpCAM**	PerCP Cy5.5	9C4	Biolegend
**Podoplanin (GP38)**	PE Dazzle	NC-08	Biolegend
**CD105**	BV421	43A3	Biolegend
**CD31**	BV650	WM59	BD
**CD73**	BV785	AD2	Biolegend
**CD49a**	BUV395	SR84	Biolegend

Supplementary table 2b.

Flow cytometry antibodies and clones used for staining human endometrial tissue following tissue digest.

Supplementary table 3

Marker	Clone	Metal	Manufacturer
**aSMA**	1E9A11	Pr141	Invitrogen
**Vimentin**	O91D3	Nd143	Biolegend
**CD14**	D7A2T	Nd144	Cell Signalling
**CD68**	KP1	Nd145	Biolegend
**CD163**	EDHu-1	Sm147	BIO-RAD
**Heparan sulfate**	10E4	Nd146	Amsbio
**CD206**	E2L9N	Sm149	Cell signalling
**PanCK**	C-11	Nd148	Biolegend
**Syndecan-1**	EPR6454	Nd150	Abcam
**CD31**	JC/70A	Eu151	NOVUS
**CD45**	2B11	Sm152	Invitrogen
**CD44**	IM7	Eu153	Biolegend
**CD127**	EPR23747-333	Sm154	Abcam
**CD56**	123C3	Gd155	BIO-RAD
**CD4**	EPR6855	Gd156	Abcam
**NKG2a**	EPR23737-127	Gd158	Abcam
**NKp46**	195314	Gd160	R&D systems
**CD20**	H1	Dy161	BD Biosciences
**CD8**	C8/144B	Dy162	Biolegend
**CD19**	EPR6906	Dy163	Abcam
**VEGF**	G153-694	Ho165	BD Biosciences
**PDGFRa**	EPR5480	Er166	Abcam
**Granzyme B**	496B	Er167	Thermofisher Scientific
**Ki67**	B56	Er168	Fisher scientific
**CD3**	D7A6E	Er170	Cell signalling
**Collagen**	UNLB	Yb172	Southern Biotech
**CD45RO**	UCHL1	Yb173	Biolegend
**HLA-DR**	TAL1B5	Yb174	Abcam
**ClCaspase3**	5A1E	Lu175	Cell signalling
**DNA191Ir**	201192A	Ir191	Standard Biotools
**DNA193Ir**	201192A	Ir193	Standard Biotools

Supplementary table 3.
Hyperion IMC antibodies and clones used for staining human endometrial tissue (FFPE sections).

SUPPLEMENTARY NOTE 1: Single Cell RNAsequencing methods

Library preparation and sequencing

Library Preparation

Gene expression libraries were prepared from single cells using the Chromium Controller and Single Cell 3' Reagent Kits v3 (10x Genomics, Inc. Pleasanton, USA) according to the manufacturer's protocol (CG000183 Rev A). Briefly, nanoliter-scale Gel Beads-in-emulsion (GEMs) were generated by combining barcoded Gel Beads, a master mix containing cells, and partitioning oil onto a Chromium chip. Cells were delivered at a limiting dilution, such that the majority (90-99%) of generated GEMs contain no cell, while the remainder largely contain a single cell. The Gel Beads were then dissolved, primers released, and any co-partitioned cells lysed. Primers containing an Illumina TruSeq Read 1 sequencing primer, a 16-nucleotide 10x Barcode, a 12-nucleotide unique molecular identifier (UMI) and a 30-nucleotide poly(dT) sequence were then mixed with the cell lysate and a master mix containing reverse transcription (RT) reagents. Incubation of the GEMs then yielded barcoded cDNA from poly-adenylated mRNA. Following incubation, GEMs were broken and pooled fractions recovered. First-strand cDNA was then purified from the post GEM-RT reaction mixture using silane magnetic beads and amplified via PCR to generate sufficient mass for library construction. Enzymatic fragmentation and size selection were then used to optimize the cDNA amplicon size. Illumina P5 & P7 sequences, a sample index, and TruSeq Read 2 sequence were added via end repair, A-tailing, adaptor ligation, and PCR to yield final Illumina-compatible sequencing libraries.

Sequencing

The resulting sequencing libraries comprised standard Illumina paired-end constructs flanked with P5 and P7 sequences. The 16 bp 10x Barcode and 12 bp UMI were encoded in Read 1, while Read 2 was used to sequence the cDNA fragment. Sample index sequences were incorporated as the i7 index read. Paired-end sequencing (28:98) was performed on the Illumina NextSeq500 platform using NextSeq 500/550 High Output v2.5 (150 Cycles) reagents.

Single cell RNAseq analysis

Raw sequencing data from the myeloid population and $CD45^-$ cells were independently processed using the 10x Genomics Cell Ranger pipeline (v6.1.2). Base call (BCL) files generated by the sequencer were converted to FASTQ files using "cellranger mkfastq". The FASTQ files were then mapped against the pre-built mouse reference package from 10X Genomics (mm10-2020-A) using "cellranger count" to produce the gene-cell barcode matrix of the given sample.

The single-cell data were processed in R environment (v4.1) following the workflow documented in Orchestrating Single-Cell Analysis with Bioconductor[1]. Briefly, for each sample, the HDF5 file generated by Cell Ranger was imported into R to create a SingleCellExperiment object. A combination of median absolute deviation (MAD), as implemented by the “isOutlier” function in the scuttle R package (v1.4.0) and exact thresholds was used to identify and subsequently remove low quality cells.

To compute deconvolution size factors, we first used the “quickCluster” function to group cells into clusters of similar expression, then use the “computeSumFactors” function to normalise for cell-specific biases to compute size factors for each cell. Both functions are available from the scran R package (v1.22.1). Normalised expression values were computed using the “calculateCPM” function from the scuttle R package, which make use of the size factors calculated previously, and transformed to log2-scale.

The per-gene variance of the log-expression profile was modelled using the “modelGeneVarByPoisson” function from the scran R package (v1.22.1) and top 2000 highly variable genes (HVGs) were selected. We performed principal component analysis (PCA) on the selected HVGs using the “runPCA “ function from the scater R package (v1.22.0), and the first 20 dimensions of the PCA were used as input to produce the t-stochastic neighbour embedding (t-SNE) projection using the “runTSNE” function from the scater R package. Putative cell clusters were identified using the Louvain algorithm from the igraph R package (v 1.2.11).

To identify cluster-defining marker genes, Wilcox Rank Sum tests were performed between clusters with a minimum log fold change of 0.25 and minimum fraction of cells set to 0.25. Clusters were manually annotated as cell subsets based on marker gene expression. Gene-set enrichment analysis was performed between macrophage and monocyte subsets, based on identified marker genes, for biological process (BP) Gene Ontology (GO) terms using the clusterProfiler package (v 4.14.0). To define significantly differentially expressed genes within BP GO terms, differential gene expression analysis was performed between clusters using DESeq2. Significantly differentially expressed genes were identified as those with an adjusted p value < 0.01 and an absolute log2FoldChange > 1. Heatmaps of BP GO terms represent genes in that pathway which are significantly differentially expressed between at least two clusters. Pseudotime analysis was performed using the slingshot package (v 2.14.0).

1. Amezquita, R.A. *et al.* Orchestrating single-cell analysis with Bioconductor. *Nat Methods* **17**, 137-145 (2020).

SUPPLEMENTARY NOTE 2: Bulk RNA sequencing methods

Sequencing and library preparation

Total RNA was submitted to the Genomic Technologies Core Facility (GTCF). Quality and integrity of the RNA samples were assessed using a 4200 TapeStation (Agilent Technologies) and then libraries generated using the *Illumina® Stranded mRNA Prep. Ligation* kit (Illumina, Inc.) according to the manufacturer's protocol. Briefly, total RNA (typically 0.025-1ug) was used as input material from which polyadenylated mRNA was purified using poly-T, oligo-attached, magnetic beads. Next, the mRNA was fragmented under elevated temperature and then reverse transcribed into first strand cDNA using random hexamer primers and in the presence of Actinomycin D (thus improving strand specificity whilst mitigating spurious DNA-dependent synthesis). Following removal of the template RNA, second strand cDNA was then synthesized to yield blunt-ended, double-stranded cDNA fragments. Strand specificity was maintained by the incorporation of deoxyuridine triphosphate (dUTP) in place of dTTP to quench the second strand during subsequent amplification. Following a single adenine (A) base addition, adapters with a corresponding, complementary thymine (T) overhang were ligated to the cDNA fragments. Pre-index anchors were then ligated to the ends of the double-stranded cDNA fragments to prepare them for dual indexing. A subsequent PCR amplification step was then used to add the index adapter sequences to create the final cDNA library. The adapter indices enabled the multiplexing of the libraries, which were pooled prior to loading on to the appropriate flow-cell. This was then paired-end sequenced (59 + 59 cycles, plus indices) on an Illumina NovaSeq6000 instrument. Finally, the output data was demultiplexed and BCL-to-Fastq conversion performed using Illumina's bcl2fastq software, version 2.20.0.422.

Bulk RNAseq analysis

Unmapped paired-reads of 59bp were assessed using a quality control pipeline consisting of FastQC v0.11.3 (http://www.bioinformatics.babraham.ac.uk/proteins/fastqc/), FastQ Screen v0.14.0 (https://www.bioinformatics.babraham.ac.uk/proteins/fastq_screen/), QualiMap v2.2.2-dev and RSeQC v4.0.0. The reads were trimmed to remove any adapter or poor quality sequence using Trimmomatic v0.39. The first poor quality base of each read was removed, and reads were truncated at a sliding 4bp window, starting 5', with a mean quality <Q20, and removed if the final length was less than 35bp. Additional flags included: 'ILLUMINACLIP:./Truseq3-PE-2_Nextera-PE.fa:2:30:10 HEADCROP:1 SLIDINGWINDOW:4:20 MINLEN:35'.

The filtered reads were mapped to the human reference sequence analysis set (hg38/Dec. 2013/GRCh38) from the UCSC browser using STAR v2.7.7a. The genome index was created using the comprehensive Gencode v43 gene annotation applying a flag suitable for the read length (--sjdbOverhang 58). During mapping the flags '--quantMode GeneCounts' was used to generate read counts into genes. Transcripts with fewer than 100 total counts across all samples were removed prior to normalisation and differential expression. Read count normalisation and differential expression were performed in R using the DESeq2 package (v 1.46.0). To compare cell types, a factor was used to represent the individuals contributing the samples, and a second representing the cell type itself. Log fold change shrinkage was applied using the ashr package (v 2.2.63). Principle components analysis was performed using the "prcomp" function on variance stabilization transformed values for the 250 genes with the highest variance in the dataset. Genes were considered differentially expressed between cell types when adjusted p value (padj) < 0.05 and the absolute log2 fold change > 1. The top 50 differentially expressed genes were identified by selecting the 200 genes with the smallest padj then selecting from those genes the 50 with the greatest absolute log 2 fold change. Signature genes from our mouse scRNAseq data were identified by ranking genes by their average log2 foldchange and selecting the 25 genes with the greatest values. Where possible these genes were matched to their human gene homologs. Gene set enrichment analysis for gene ontology (GO) biological process (BP) terms was performed using the clusterProfiler package (v 4.14.0) with a p value threshold of 0.05. GO BP terms were simplified using the 'simply' function with a cutoff of 0.3.
