# Supplementary material for: The dual roles of uterine monocytes in regulation of tissue homeostasis throughout reproductive cycles in health and disease
Source: Nat Immunol. 2026 Sep 8;27(10):2058–73. doi: 10.1038/s41590-026-02651-y (PMC13630565; doi:10.1038/s41590-026-02651-y)
Supplement: Supplementary file 2 [file 41590_2026_2651_MOESM2_ESM.pdf]

nature portfolio

Corresponding author(s): Elizabeth Mann

Last updated by author(s): 21/07/26

Reporting Summary

Nature Portfolio wishes to improve the reproducibility of the work that we publish. This form provides structure for consistency and transparency in reporting. For further information on Nature Portfolio policies, see our Editorial Policies and the Editorial Policy Checklist.

Statistics

For all statistical analyses, confirm that the following items are present in the figure legend, table legend, main text, or Methods section.

n/a	Confirmed	
☐	☒	The exact sample size (*n*) for each experimental group/condition, given as a discrete number and unit of measurement
☐	☒	A statement on whether measurements were taken from distinct samples or whether the same sample was measured repeatedly
☐	☒	The statistical test(s) used AND whether they are one- or two-sided<br>*Only common tests should be described solely by name; describe more complex techniques in the Methods section.*
☐	☒	A description of all covariates tested
☐	☒	A description of any assumptions or corrections, such as tests of normality and adjustment for multiple comparisons
☐	☒	A full description of the statistical parameters including central tendency (e.g. means) or other basic estimates (e.g. regression coefficient) AND variation (e.g. standard deviation) or associated estimates of uncertainty (e.g. confidence intervals)
☐	☒	For null hypothesis testing, the test statistic (e.g. *F*, *t*, *r*) with confidence intervals, effect sizes, degrees of freedom and *P* value noted<br>*Give P values as exact values whenever suitable.*
☒	☐	For Bayesian analysis, information on the choice of priors and Markov chain Monte Carlo settings
☒	☐	For hierarchical and complex designs, identification of the appropriate level for tests and full reporting of outcomes
☒	☐	Estimates of effect sizes (e.g. Cohen's *d*, Pearson's *r*), indicating how they were calculated

Our web collection on statistics for biologists contains articles on many of the points above.

Software and code

Policy information about availability of computer code

Data collection: No software was used

Data analysis: Flow cytometry analysis: FlowJo (version 10, Treestar)
Generation of graphs and statistical analysis: GraphPad Prism version 10
Predicted receptor-ligand interactions from murine myeloid and CD45- single cell datasets: CellChat (v2.1.0)
QuPath (version 0.7.0) and ImageJ (version 1.54t) were utilised for image analysis and configuration for immunoflourescent microscopy.

Single cell RNA sequencing: Raw sequencing data from the myeloid population and CD45- cells were independently processed using the 10x Genomics Cell Ranger pipeline (v6.1.2). The single-cell data were processed in R environment (v4.1). Briefly, for each sample, the HDF5 file generated by Cell Ranger was imported into R to create a SingleCellExperiment object. A combination of median absolute deviation (MAD), as implemented by the "isOutlier" function in the scuttle R package (v1.4.0) and exact thresholds was used to identify and subsequently remove low quality cells.

To compute deconvolution size factors, we first used the "quickCluster" function to group cells into clusters of similar expression, then use the "computeSumFactors" function to normalise for cell-specific biases to compute size factors for each cell. Both functions are available from the scran R package (v1.22.1). Normalised expression values were computed using the "calculateCPM" function from the scuttle R package, which make use of the size factors calculated previously, and transformed to log2-scale.

The per-gene variance of the log-expression profile was modelled using the "modelGeneVarByPoisson" function from the scran R package (v1.22.1) and top 2000 highly variable genes (HVGs) were selected. We performed principal component analysis (PCA) on the selected HVGs using the "runPCA " function from the scater R package (v1.22.0), and the first 20 dimensions of the PCA were used as input to produce the t-

stochastic neighbour embedding (t-SNE) projection using the “runTSNE” function from the scater R package. Putative cell clusters were identified using the Louvain algorithm from the igraph R package (v 1.2.11).

To identify cluster-defining marker genes, Wilcox Rank Sum tests were performed between clusters with a minimum log fold change of 0.25 and minimum fraction of cells set to 0.25. Clusters were manually annotated as cell subsets based on marker gene expression. Gene-set enrichment analysis was performed between macrophage and monocyte subsets, based on identified marker genes, for biological process (BP) Gene Ontology (GO) terms using the clusterProfiler package (v 4.14.0). To define significantly differentially expressed genes within BP GO terms, differential gene expression analysis was performed between clusters using DESeq2. Significantly differentially expressed genes were identified as those with an adjusted p value < 0.01 and an absolute log2FoldChange > 1. Heatmaps of BP GO terms represent genes in tha

t pathway which are significantly differentially expressed between at least two clusters. Pseudotime analysis was performed using the slingshot package (v 2.14.0).

Bulk RNAseq: Read count normalisation and differential expression were performed in R using the DESeq2 package (v 1.46.0). To compare cell types, a factor was used to represent the individuals contributing the samples, and a second representing the cell type itself. Log fold change shrinkage was applied using the ashr package (v 2.2.63). Principle components analysis was performed using the “prcomp” function on variance stabilization transformed values for the 250 genes with the highest variance in the dataset. Genes were considered differentially expressed between cell types when adjusted p value (padj) < 0.05 and the absolute log2 fold change > 1. The top 50 differentially expressed genes were identified by selecting the 200 genes with the smallest padj then selecting from those genes the 50 with the greatest absolute log 2 fold change. Signature genes from our mouse scRNAseq data were identified by ranking genes by their average log2 foldchange and selecting the 25 genes with the greatest values. Where possible these genes were matched to their human gene homologs. Gene set enrichment analysis for gene ontology (GO) biological process (BP) terms was performed using the clusterProfiler package (v 4.14.0) with a p value threshold of 0.05. GO BP terms were simplified using the ‘simply’ function with a cutoff of 0.3.

Hyperion spatial analyses: Cellcharter (v0.3.5) was utilised to determine the proximity of clusters of cells to other cells in tissue niches. To quantify spatial differences between cell populations, we computed nearest-neighbour distances per ROI using a permutation bootstrap. For each cell, we measured distance to the nearest cell of each population, then compared observed values with ROI-matched null distributions generated by shuffling population labels (preserving coordinates and composition). Cell-cell interaction enrichment was also assessed with Squidpy using a 20um neighbourhood threshold (<20um between cells). Z-scores were calculated against complete spatial randomness using 1000 within-region label permutations: positive scores indicate enriched interactions and negative scores indicate depleted interactions.

For manuscripts utilizing custom algorithms or software that are central to the research but not yet described in published literature, software must be made available to editors and reviewers. We strongly encourage code deposition in a community repository (e.g. GitHub). See the Nature Portfolio guidelines for submitting code & software for further information.

Data

Policy information about availability of data

All manuscripts must include a data availability statement. This statement should provide the following information, where applicable:

- Accession codes, unique identifiers, or web links for publicly available datasets
- A description of any restrictions on data availability
- For clinical datasets or third party data, please ensure that the statement adheres to our policy

Raw sequencing data has been deposited in the ArrayExpress database at EMBL-EBI under the following accession numbers: RNAseq (human): E-MTAB-15397. Single cell RNAseq (murine) E-MTAB-15391.

Research involving human participants, their data, or biological material

Policy information about studies with human participants or human data. See also policy information about sex, gender (identity/presentation), and sexual orientation and race, ethnicity and racism.

Reporting on sex and gender	Females only used in this study as tissue studied is the uterus.
Reporting on race, ethnicity, or other socially relevant groupings	Ethnicity subcategories were defined by the researchers (White, Black, Asian, Mixed race) with ethnic identity of participants determined by standard National Health Service (NHS) reporting systems (defined by participants).
Population characteristics	Female healthy controls (endometrial biopsies taken during colposcopy for research purposes, during colposcopy for HPV screening or during hysteroscopy for investigation of subfertility with normal endometrium and no inflammatory or reproductive diseases). Samples were also taken from patients with uterine fibrosis (Asherman syndrome) during hysteroscopy or surgery. All participants were reproductive age (18-43) to prevent confounding effects of hormone changes on inflammation in peri- or post-menopausal women.
Recruitment	All patients provided written, informed consent for collection of tissue samples and subsequent analyses. Samples were obtained according to the principles expressed in the Declaration of Helsinki and under local ethical guidelines.
Ethics oversight	REC approval numbers: 19/NS/0163 and 21/NW/0195

Note that full information on the approval of the study protocol must also be provided in the manuscript.

Field-specific reporting

Please select the one below that is the best fit for your research. If you are not sure, read the appropriate sections before making your selection.

☒ Life sciences ☐ Behavioural & social sciences ☐ Ecological, evolutionary & environmental sciences

For a reference copy of the document with all sections, see nature.com/documents/nr-reporting-summary-flat.pdf

Life sciences study design

All studies must disclose on these points even when the disclosure is negative.

Sample size

Mouse work: sample sizes were based on investigators own previous data comparing myeloid cell subsets in inflammatory and non-inflammatory conditions (e.g. myeloid cells in the intestine +/- antibiotics). Calculations estimated that to detect a 20% difference with approximately 10% standard deviation, 6 animals per group (cycle stage in this case) would be required. However in some cases there are many more numbers than this e.g. monocyte or macrophage numbers as these can be calculated across all experiments (e.g. sets of experiments assessing cytokine production, different cell surface markers etc.).

Human work: limitations for availability of (control) tissue so we used all available samples from our sampling programmes but as above we attempted to include at least n=6 per group.

Data exclusions

No data exclusions

Replication

All experiments were succesfully repeated at least twice (two independent experiments) but for nearly all this was more than twice.

Randomization

Samples in both mice and humans were taken before knowing cycle stage hence no bias or selection.

Blinding

Investigators were not blinded.
Mouse work: for comparison of reproductive cycle stages in mice, mice were staged after culling (via vaginal lavage) due to Home Office license restrictions thus could not be allocated/randomized by someone else prior to each experiment starting. For comparison of anti-cytokine treatment and PBS and WT versus Ccr2-/- – the same investigator carried out all experiments including analysis so it was not possible to blind.
Human work: investigators were blinded to some degree during tissue collection and processing since clinical data for samples was received days/weeks later.

Behavioural & social sciences study design

All studies must disclose on these points even when the disclosure is negative.

Study description

Briefly describe the study type including whether data are quantitative, qualitative, or mixed-methods (e.g. qualitative cross-sectional, quantitative experimental, mixed-methods case study).

Research sample

State the research sample (e.g. Harvard university undergraduates, villagers in rural India) and provide relevant demographic information (e.g. age, sex) and indicate whether the sample is representative. Provide a rationale for the study sample chosen. For studies involving existing datasets, please describe the dataset and source.

Sampling strategy

Describe the sampling procedure (e.g. random, snowball, stratified, convenience). Describe the statistical methods that were used to predetermine sample size OR if no sample-size calculation was performed, describe how sample sizes were chosen and provide a rationale for why these sample sizes are sufficient. For qualitative data, please indicate whether data saturation was considered, and what criteria were used to decide that no further sampling was needed.

Data collection

Provide details about the data collection procedure, including the instruments or devices used to record the data (e.g. pen and paper, computer, eye tracker, video or audio equipment) whether anyone was present besides the participant(s) and the researcher, and whether the researcher was blind to experimental condition and/or the study hypothesis during data collection.

Timing

Indicate the start and stop dates of data collection. If there is a gap between collection periods, state the dates for each sample cohort.

Data exclusions

If no data were excluded from the analyses, state so OR if data were excluded, provide the exact number of exclusions and the rationale behind them, indicating whether exclusion criteria were pre-established.

Non-participation

State how many participants dropped out/declined participation and the reason(s) given OR provide response rate OR state that no participants dropped out/declined participation.

Randomization

If participants were not allocated into experimental groups, state so OR describe how participants were allocated to groups, and if allocation was not random, describe how covariates were controlled.

Ecological, evolutionary & environmental sciences study design

All studies must disclose on these points even when the disclosure is negative.

Study description	*Briefly describe the study. For quantitative data include treatment factors and interactions, design structure (e.g. factorial, nested, hierarchical), nature and number of experimental units and replicates.*
Research sample	*Describe the research sample (e.g. a group of tagged Passer domesticus, all Stenocereus thurberi within Organ Pipe Cactus National Monument), and provide a rationale for the sample choice. When relevant, describe the organism taxa, source, sex, age range and any manipulations. State what population the sample is meant to represent when applicable. For studies involving existing datasets, describe the data and its source.*
Sampling strategy	*Note the sampling procedure. Describe the statistical methods that were used to predetermine sample size OR if no sample-size calculation was performed, describe how sample sizes were chosen and provide a rationale for why these sample sizes are sufficient.*
Data collection	*Describe the data collection procedure, including who recorded the data and how.*
Timing and spatial scale	*Indicate the start and stop dates of data collection, noting the frequency and periodicity of sampling and providing a rationale for these choices. If there is a gap between collection periods, state the dates for each sample cohort. Specify the spatial scale from which the data are taken*
Data exclusions	*If no data were excluded from the analyses, state so OR if data were excluded, describe the exclusions and the rationale behind them, indicating whether exclusion criteria were pre-established.*
Reproducibility	*Describe the measures taken to verify the reproducibility of experimental findings. For each experiment, note whether any attempts to repeat the experiment failed OR state that all attempts to repeat the experiment were successful.*
Randomization	*Describe how samples/organisms/participants were allocated into groups. If allocation was not random, describe how covariates were controlled. If this is not relevant to your study, explain why.*
Blinding	*Describe the extent of blinding used during data acquisition and analysis. If blinding was not possible, describe why OR explain why blinding was not relevant to your study.*

Did the study involve field work? ☐ Yes ☐ No

Field work, collection and transport

Field conditions	*Describe the study conditions for field work, providing relevant parameters (e.g. temperature, rainfall).*
Location	*State the location of the sampling or experiment, providing relevant parameters (e.g. latitude and longitude, elevation, water depth).*
Access & import/export	*Describe the efforts you have made to access habitats and to collect and import/export your samples in a responsible manner and in compliance with local, national and international laws, noting any permits that were obtained (give the name of the issuing authority, the date of issue, and any identifying information).*
Disturbance	*Describe any disturbance caused by the study and how it was minimized.*

Reporting for specific materials, systems and methods

We require information from authors about some types of materials, experimental systems and methods used in many studies. Here, indicate whether each material, system or method listed is relevant to your study. If you are not sure if a list item applies to your research, read the appropriate section before selecting a response.

Materials & experimental systems

n/a	Involved in the study	
☐	☒	Antibodies
☐	☐	Eukaryotic cell lines
☐	☐	Palaeontology and archaeology
☐	☒	Animals and other organisms
☐	☒	Clinical data
☐	☐	Dual use research of concern
☐	☐	Plants

Methods

n/a	Involved in the study	
☐	☐	ChIP-seq
☐	☒	Flow cytometry
☐	☐	MRI-based neuroimaging

Antibodies

Antibodies used

Flow cytometry anti-mouse:
Armenian hamster CD11c-BV785 Clone N416 Manufacturer Biolegend Cat number 117336 used at 1:100
Rat Ly6C-PerCPCy5.5 Clone 5HK1.4 Manufacturer Biolegend Cat number 128012 used at 1:100
Mouse CD64-APC and PeCy7 Clone X54-5/7.1 Manufacturer Biolegend Cat number 139306 (APC) and 139314 (PeCy7) used at 1:100
Rat MHC class II AF700 Clone M5/114.15.2 Manufacturer Biolegend Cat number 107622 used at 1:100
Fixable viability dye eFluor 780 Manufacturer: Fisher Scientific Cat number 50-112-9035 used at 1:1000
Rat CD206-BV650 Clone C068C2 Manufacturer Biolegend Cat number 141723 used at 1:100
Rat Ly6G-PerCPCy5.5/BV510 Clone 1A8 Manufacturer Biolegend Cat number 127616 and 127633 (BV510) used at 1:100
Rat Siglec F-BV650 Clone E20-2440 Manufacturer BD Cat number 740557 used at 1:100
Rat Siglec F-BV421 Clone S17007L Manufacturer Biolegend Cat number 155509 used at 1:100
Rat Perforin-FITC Clone S16009A Manufacturer Biolegend Cat number 154310 used at 1:100
Mouse Granzyme B-Pacific Blue Clone GB11 Manufacturer Biolegend Cat number 515408 used at 1:100
Rat CD45-FITC and BV510 Clone 30-F11 Manufacturer Biolegend Cat number 103108 (FITC) and 103138 (BV510) used at 1:100
Rat CD11b-BUV395 Clone M1/70 Manufacturer BD Cat number 565976 used at 1:100
Rat CCR2-FITC Clone SA203G11 Manufacturer Biolegend Cat number 150608 used at 1:100
Mouse CX3CR1-BV605 Clone SA011F11 Manufacturer Biolegend Cat number 149027 used at 1:100
Rat CD31-BV785 Clone 390 Manufacturer Biolegend Cat number 102435 used at 1:100
Armenian Hamster CD29-Pacific Blue Clone HMB1-1 Manufacturer Biolegend Cat number 102224 used at 1:100
Rat CD34 AF700 Clone RAM34 Manufacturer ThermoFisher Scientific Cat number 56-0341-82 used at 1:100
Syrian hamster Podoplanin PE Dazzle Clone 8.1.1. Manufacturer Biolegend Cat number 127420 used at 1:100
Rat EpCAM BV650 Clone G8.8 Manufacturer Biolegend Cat number 118241 used at 1:100
Rat PDGFRalpha (CD140a) BV605 Clone APA5 Manufacturer Biolegend Cat number 135916 used at 1:100
Rat CD9 FITC Clone MZ3 Manufacturer Biolegend Cat number 124808 used at 1:100
Rat CD106 PerCPCy5.5 Clone 429 (MVCAM.A) Manufacturer Biolegend Cat number 105716 used at 1:100
Rat CD146 PeCy7 Clone ME-9F1 Manufacturer Biolegend Cat number 134714 used at 1:100
Mouse CD90-PE Clone 5a-8 Manufacturer Thermofisher Scientific Cat number MA5-17749 used at 1:100
Rat TNFalpha APC Clone MP6-XT22 Manufacturer Biolegend Cat number 506308 used at 1:100
Rat MerTK-PE Clone 2B10C42 Manufacturer Biolegend Cat number 151506 used at 1:100
Rat CD24 PE Dazzle Clone M1/69 Manufacturer Biolegend Cat number 101838 used at 1:100

Flow cytometry anti-human:
Mouse CD11c AF700 Clone Bu15 Manufacturer Biolegend Cat number 337220 used at 1:100
Mouse CD14 PE Clone M5E2 Manufacturer Biolegend Cat number 301850 used at 1:100
Mouse CD64 APC Clone 10.1 Manufacturer Biolegend Cat number 305014 used at 1:100
Mouse HLA-DR BV785 Clone L243 Manufacturer Biolegend Cat number 307642 used at 1:100
Mouse CD206 BV650 Clone 19.2 Manufacturer BD Cat number 740598 used at 1:100
Mouse CD64 PeCy7 Clone AER-37 (CRA-1) Manufacturer Biolegend Cat number 334620 used at 1:100
Mouse Perforin-FITC Clone Dg9 Manufacturer Thermofisher Scientific Cat number 11-9994-42 used at 1:100
Mouse Granzyme B Texas Red Clone GB11 Manufacturer Thermofisher Scientific Cat number GRB17 used at 1:100
Mouse CD45 BV510 Clone HI30 Manufacturer Biolegend Cat number 304036 used at 1:100
Mouse CD90 BV605 Clone 5E10 Manufacturer Biolegend Cat number 328128 used at 1:100
Mouse CD20 AF700 Clone 2H7 Manufacturer Biolegend Cat number 302322 used at 1:100
Mouse CD19 AF700 Clone HIB19 Manufacturer Thermofisher Scientific Cat number 56-0199-42 used at 1:100
Mouse CCR2 PeCy7 and BV421 Clone K036C2 Manufacturer Biolegend Cat numbers 357212 (PeCy7) and 357210 (BV421) used at 1:100
Mouse CD3 PeCy7 Clone UCHT1 Manufacturer Biolegend Cat number 300420 used at 1:100
Mouse EpCAM PerCPCy5.5 Clone 9C4 Manufacturer Biolegend Cat number 324214 used at 1:100
Rat Podoplanin (Gp38) PE Dazzle Clone NC-08 Manufacturer Biolegend Cat number 337028 used at 1:100
Mouse CD31 BV650 Clone WM59 Manufacturer Biolegend Cat number 303122 used at 1:100
Mouse Ki67 BV605 Clone Ki-67 Manufacturer Biolegend Cat number 350522 used at 1:100
Mouse TNFalpha BUV395 Clone Mab11 Manufacturer BD Cat number 563996 used at 1:100
Fixable viability dye eFluor 780 Manufacturer: Fisher Scientific Cat number 50-112-9035 used at 1:1000

Hyperion anti-human
Mouse aSMA Pr141 Clone 1E9A11 Manufacturer Invitrogen Cat number 14-9760-82 used at 1:200
Mouse Vimentin Nd143 Clone O91D3 Manufacturer Biolegend Cat number 677802 used at 1:200
Rat CD14 Nd144 Clone D7A2T Manufacturer Biolegend Cat number 43878SF used at 1:200
Mouse CD68 Nd145 Clone KP1 Manufacturer Biolegend Cat number 916104 used at 1:200
Mouse CD163 Sm147 Clone EdHu-1 Manufacturer BIO-RAD Cat number MCA1853 used at 1:200
Mouse Heparan sulfate Nd146 Clone 10E4 Manufacturer Amsbio Cat number 370255-1 used at 1:200
Rabbit CD206 Sm149 Clone E2L9N Manufacturer Cell signalling Cat number 49243SF used at 1:200
Mouse Pan CK Nd148 Clone C-11 Manufacturer Biolegend Cat number 628601 used at 1:200
Rabbit Syndecan-1 Nd150 Clone EPR6454 Manufacturer Abcam Cat number AB226108 used at 1:200
Mouse CD31 Eu151 Clone JC/70A Manufacturer NOVUS Cat number NBP2-80641 used at 1:200
Mouse CD45 Sm152 Clone 2B11 Manufacturer Invitrogen Cat number 14-9457-82 used at 1:200
Rat CD44 Eu153 Clone IM7 Manufacturer Biolegend Cat number 103002 used at 1:200
Rabbit CD127 Sm154 Clone EPR23747-333 Manufacturer Abcam Cat number AB282011 used at 1:200
Mouse CD56 Gd155 Clone 123C3 Manufacturer BIO-RAD Cat number MCA2693GA used at 1:200
Rabbit CD4 Gd156 Clone EPR6855 Manufacturer Abcam Cat number AB181724 used at 1:200
Rabbit NKG2a Gd158 Clone EPR23737-127 Manufacturer Abcam Cat number AB273516 used at 1:200
Mouse NKp46 Gd160 Clone 195314 Manufacturer R&D Systems Cat number MAB1850 used at 1:200
Mouse CD20 Dy161 Clone H1 Manufacturer BD Biosciences Cat number 372902 used at 1:200

Mouse CD8 Dy162 Clone C8/144B Manufacturer Biolegend Cat number 372902 used at 1:200
Rabbit CD19 Dy163 Clone EPR6906 Manufacturer Abcam Cat number AB271904 used at 1:200
Mouse VEGF Ho165 Clone G153-694 Manufacturer BD Biosciences Cat number G153-694 used at 1:200
Rabbit PDGFRa Er166 Clone EPR5480 Manufacturer Abcam Cat number AB248689 used at 1:200
Rat Granzyme B Er167 Clone 496B Manufacturer Thermofisher Scientific Cat number 14-8889-82 used at 1:200
Mouse Ki67 Er168 Clone B56 Manufacturer Fisher Scientific Cat number 15898578 used at 1:200
Rabbit CD3 Er170 Clone D7A6E Manufacturer Cell signalling Cat number 24581SF used at 1:200
Goat Collagen Yb172 Clone UNLB Manufacturer Southern Biotech Cat number 1310-01 used at 1:200
Mouse CD45RO Yb173 Clone UCHL1 Manufacturer Biolegend Cat number 304202 used at 1:200
Mouse HLA-DR Yb174 Clone TAL1B5 Manufacturer Abcam Cat number AB176408 used at 1:200
Rabbit Cleaved caspase 3 Lu175 Clone 5A1E Manufacturer Cell signalling Cat number 94530SF used at 1:200
DNA191lr Ir191 Clone 201192A Manufacturer Standard Biotools. Cat number 201192A used at 1:400

Validation

Flow cytometry antibodies were all pre-validated by Biolegend, Thermofisher Scientific, or BD Biosciences with numerous examples on their websites for each antibody. All antibodies used are commercially available. In addition, we have previously used these antibodies to successfully distinguish and characterise monocyte and macrophage subsets in other primary tissues in humans (blood: PMIDs 32943497, 36922030 and mice (intestine: PMIDs 30355800, 35199335). In all cases, gating was set according to reference staining of control samples stained with isotype-matched controls.

For Hyperion analyses, some antibodies were validated ourselves by Immunofluorescent microscopy (Supplementary figures) and all clones were originally validated on appropriate human tissues, e.g. appendix and tonsil by the Flow Cytometry core facility at the University of Manchester.

Eukaryotic cell lines

Policy information about cell lines and Sex and Gender in Research

Cell line source(s)

State the source of each cell line used and the sex of all primary cell lines and cells derived from human participants or vertebrate models.

Authentication

Describe the authentication procedures for each cell line used OR declare that none of the cell lines used were authenticated.

Mycoplasma contamination

Confirm that all cell lines tested negative for mycoplasma contamination OR describe the results of the testing for mycoplasma contamination OR declare that the cell lines were not tested for mycoplasma contamination.

Commonly misidentified lines
(See ICLAC register)

Name any commonly misidentified cell lines used in the study and provide a rationale for their use.

Palaeontology and Archaeology

Specimen provenance

Provide provenance information for specimens and describe permits that were obtained for the work (including the name of the issuing authority, the date of issue, and any identifying information). Permits should encompass collection and, where applicable, export.

Specimen deposition

Indicate where the specimens have been deposited to permit free access by other researchers.

Dating methods | *If new dates are provided, describe how they were obtained (e.g. collection, storage, sample pretreatment and measurement), where they were obtained (i.e. lab name), the calibration program and the protocol for quality assurance OR state that no new dates are provided.*

☐ Tick this box to confirm that the raw and calibrated dates are available in the paper or in Supplementary Information.

Ethics oversight | *Identify the organization(s) that approved or provided guidance on the study protocol, OR state that no ethical approval or guidance was required and explain why not.*

Note that full information on the approval of the study protocol must also be provided in the manuscript.

Animals and other research organisms

Policy information about studies involving animals; ARRIVE guidelines recommended for reporting animal research, and Sex and Gender in Research

Laboratory animals	C57BL/6J (Charles River; strain #:000664) mice were housed and maintained at the University of Manchester, U.K. Ccr2-/- mice (C57BL/6 background) originally from The Jackson Laboratory (strain #:004999; PMID 9366570) were maintained at the University of Manchester or gifted from the University of Glasgow (Professor Gerard Graham). Congenic CD45.1 mice (C57BL/6 background, strain #:002014 PMID 11698303) were maintained at the University of Manchester. Ifngar1-/- mice (C57BL/6 background gifted from Professor Werner Müller; PMID 8456301) were maintained at the University of Manchester. All mice were housed in individually ventilated cages under specific pathogen-free conditions at the University of Manchester, UK.<br><br>For all experiments other than those involving older mice, mice were aged under 16 weeks. For experiment involving older mice, mice were aged 9-12 months.<br><br>The mice were held in 12:12 light:dark cycle, at 21°C±2°C. Humidity: 40% to 60% relative humidity. Mice had ad libitum access to standard pelleted rodent chow and fresh water, with basic bedding and nesting material. Chow constituents: moisture 12%, crude protein 18.5%, crude oils and fats 5.50%, crude fibres 4.50% and crude ash 6%.
Wild animals	n/a
Reporting on sex	females only as we are studying the uterus
Field-collected samples	n/a
Ethics oversight	Experiments involving mice were approved by the University of Manchester Animal Welfare Ethical Review Board and performed under licenses issued by the U.K. Home Office (PPLs P70/8548 (Allen) and PP8070044 (Travis). All experiments were in performed accordance with the U.K. Home Office regulations. For work performed at Institut Pasteur, animal experiments were conducted in accordance with approval of protocol number 2016-0010 by the Comité d'éthique en expérimentation animale Paris Centre et Sud and the Comités d'Ethique pour l'Expérimentation Animale Institut Pasteur (the ethics committee for animal experimentation), in application of the European Directive 2010/63 EU.

Note that full information on the approval of the study protocol must also be provided in the manuscript.

Clinical data

Policy information about clinical studies

All manuscripts should comply with the ICMJE guidelines for publication of clinical research and a completed CONSORT checklist must be included with all submissions.

Clinical trial registration	n/a
Study protocol	Samples were obtained following informed consent of participants, according to the principles expressed in the Declaration of Helsinki and under local ethical guidelines, and approved by the North of Scotland Research Ethics Service (reference number 19/NS/0163) and the North-West -Preston Research Ethics Committee (reference number 21/NW/0195). No financial compensation was provided.
Data collection	Female healthy controls (endometrial biopsies taken during colposcopy for research purposes, during colposcopy for HPV screening or during hysteroscopy for investigation of subfertility with normal endometrium and no inflammatory or reproductive diseases). Samples were also taken from patients with uterine fibrosis (Asherman syndrome) during hysteroscopy or surgery. Samples were collected at St. Mary's Hospital, Manchester University NHS Foundation Trust between 2019 and 2025.
Outcomes	Diagnosis given by the clinical team for participsnts following hysteroscopic and/or surgical investigation, combined with complex assessment of medical history. Menstrual cycle stage was assessed based on participants usual cycle length, date of their last menstrual period and the date of the procedure. Ovulation was estimated at 14 days before the start of their next predicted menstrual period, with samples taken after this date classified as secretory phase (early: d1-4 of secretory phase; mid: d5-9 of secretory phase; late: d10-14 of secretory phase) and samples taken before ovulation as proliferative phase.

Dual use research of concern

Policy information about dual use research of concern

Hazards

Could the accidental, deliberate or reckless misuse of agents or technologies generated in the work, or the application of information presented in the manuscript, pose a threat to:

No	Yes	
☒	☐	Public health
☒	☐	National security
☒	☐	Crops and/or livestock
☒	☐	Ecosystems
☒	☐	Any other significant area

Experiments of concern

Does the work involve any of these experiments of concern:

No	Yes	
☒	☐	Demonstrate how to render a vaccine ineffective
☒	☐	Confer resistance to therapeutically useful antibiotics or antiviral agents
☒	☐	Enhance the virulence of a pathogen or render a nonpathogen virulent
☒	☐	Increase transmissibility of a pathogen
☒	☐	Alter the host range of a pathogen
☒	☐	Enable evasion of diagnostic/detection modalities
☒	☐	Enable the weaponization of a biological agent or toxin
☒	☐	Any other potentially harmful combination of experiments and agents

Plants

Seed stocks

Report on the source of all seed stocks or other plant material used. If applicable, state the seed stock centre and catalogue number. If plant specimens were collected from the field, describe the collection location, date and sampling procedures.

Novel plant genotypes

Describe the methods by which all novel plant genotypes were produced. This includes those generated by transgenic approaches, gene editing, chemical/radiation-based mutagenesis and hybridization. For transgenic lines, describe the transformation method, the number of independent lines analyzed and the generation upon which experiments were performed. For gene-edited lines, describe the editor used, the endogenous sequence targeted for editing, the targeting guide RNA sequence (if applicable) and how the editor was applied.

Authentication

Describe any authentication procedures for each seed stock used or novel genotype generated. Describe any experiments used to assess the effect of a mutation and, where applicable, how potential secondary effects (e.g. second site T-DNA insertions, mosiacism, off-target gene editing) were examined.

ChIP-seq

Data deposition

☐ Confirm that both raw and final processed data have been deposited in a public database such as GEO.

☐ Confirm that you have deposited or provided access to graph files (e.g. BED files) for the called peaks.

Data access links
May remain private before publication.

For "Initial submission" or "Revised version" documents, provide reviewer access links. For your "Final submission" document, provide a link to the deposited data.

Files in database submission

Provide a list of all files available in the database submission.

Genome browser session
(e.g. UCSC)

Provide a link to an anonymized genome browser session for "Initial submission" and "Revised version" documents only, to enable peer review. Write "no longer applicable" for "Final submission" documents.

Methodology

Replicates	*Describe the experimental replicates, specifying number, type and replicate agreement.*
Sequencing depth	*Describe the sequencing depth for each experiment, providing the total number of reads, uniquely mapped reads, length of reads and whether they were paired- or single-end.*
Antibodies	*Describe the antibodies used for the ChIP-seq experiments; as applicable, provide supplier name, catalog number, clone name, and lot number.*
Peak calling parameters	*Specify the command line program and parameters used for read mapping and peak calling, including the ChIP, control and index files used.*
Data quality	*Describe the methods used to ensure data quality in full detail, including how many peaks are at FDR 5% and above 5-fold enrichment.*
Software	*Describe the software used to collect and analyze the ChIP-seq data. For custom code that has been deposited into a community repository, provide accession details.*

Flow Cytometry

Plots

Confirm that:

☒ The axis labels state the marker and fluorochrome used (e.g. CD4-FITC).

☒ The axis scales are clearly visible. Include numbers along axes only for bottom left plot of group (a 'group' is an analysis of identical markers).

☒ All plots are contour plots with outliers or pseudocolor plots.

☒ A numerical value for number of cells or percentage (with statistics) is provided.

Methodology

Sample preparation

Mouse uterus digest: Both uterine horns were removed from mice and collected in RPMI-1640 (ThermoFisher Scientific), minced using fine scissors and incubated for 40 minutes in 1.25mg/ml collagenase D (Sigma), 0.85mg/ml collagenase V (Sigma) and 1mg/ml dispase (Gibco, Invitrogen) in a shaking incubator at 37°C. Tissue was then filtered through a 100µM filter and cells were analysed by flow cytometry.

Human endometrial biopsy digest: Endometrial Pipelle biopsies were collected in 5ml PBS and centrifuged to separate tissue from PBS supernatant which was then removed. Tissue pellets were then resuspended in 0.5mg/ml collagenase VIII (Sigma), 0.65mg/ml collagenase D (Sigma), 1mg/ml dispase (Gibco, Invitrogen) and 0.015mg/ml DNAse (Sigma), and incubated for 35 minutes in a shaking incubator at 37°C. After incubation, tissue was filtered through a 100µM filter and tissue suspensions were pooled following digests. Cells were washed and red blood cell lysis was carried out using ddH2O for 10 seconds following immediately by 1/10 volume 10x PBS. Cells were then analysed by flow cytometry or used for in vitro stimulation assays.

0.2-2 x 106 cells were stained at 4°C in the dark, in PBS with 2% FCS and 2% EDTA (FACS buffer) with Fc block (Biolegend) for 20 minutes. Antibodies used are listed in Supplementary Tables 2a (mice) and 2b (human). For surface staining, cells were fixed in 2% paraformaldehyde (Sigma-Aldrich) for 30 minutes, washed and resuspended in FACS buffer before acquisition. For intracellular staining, following incubation with antibodies for surface staining, cells were washed in FACS buffer then fixed and permeabilised (eBioscience intracellular staining kit) before incubation for 60 minutes with antibodies for intracellular proteins (room temperature). Cells were washed and resuspended in perm buffer (eBioscience) before acquisition.

Instrument

LSR Fortessa (BD Biosciences) serial number H647800E6030 and BD FACS Aria serial number P656700000042

Software

FlowJo v10 (Treestar)

Cell population abundance

Purity of FACS sorted samples was >95% in all cases (sorted samples include human monocytes, Mac1 macrophages, Mac2 macrophages) assessed by running some of the sorted sample with the gating set up, prior to the sort.
Abundance (mid secretory phase of cycle for FACS sorted cells): monocytes were 1-10% of total CD45+ cells, Mac1 macrophages 1-10% of total CD45+ cells and Mac2 macrophages 40-60% of total CD45+ cells.

Gating strategy

In all cases cells were first pre-gated to exclude cellular debris and other non cellular components on a basis of FSC and SSC, followed by exclusion of doublets by FSC-A versus FSC-H.

Murine monocytes: Live (negative for live/dead dye) CD45+Ly6G-SiglecF-CD64+CD11b+Ly6C+
Murine Mac1 macrophages: Live (negative for live/dead dye) CD45+Ly6G-SiglecF-CD64+CD11b+Ly6C-CD11c+CD206-
Murine Mac2 macrophages: Live (negative for live/dead dye) CD45+Ly6G-SiglecF-CD64+CD11b+Ly6C-CD11c-CD206+
Murine epithelial: Live (negative for live/dead dye) CD45-EpCAM+CD31-
Murine endothelial: Live (negative for live/dead dye) CD45-EpCAM-CD31+
Murine fibroblasts: Live (negative for live/dead dye) CD45-EpCAM-CD31-PDGFRalpha+
Murine myofibroblasts: Live (negative for live/dead dye) CD45-EpCAM-CD31-PDGFRalpha+Podoplanin+CD90-

Murine pericytes: Live (negative for live/dead dye) CD45-EpCAM-CD31-PDGFRalpha-MCAM+CD29+
Murine smooth muscle cells: (negative for live/dead dye) CD45-EpCAM-CD31-PDGFRalpha-MCAM+CD9-CD90-
Human monocytes: Live (negative for live/dead dye) CD45+CD3-CD19-CD66b-Siglec8-HLA-DR+CD64+CD14+CCR2+
Human Mac1 macrophages: Live (negative for live/dead dye) CD45+CD3-CD19-CD66b-Siglec8-HLA-DR+CD64+CD14+CCR2-CD206-
Human Mac2 macrophages: Live (negative for live/dead dye) CD45+CD3-CD19-CD66b-Siglec8-HLA-DR+CD64+CD14+CCR2-CD206+
Human epithelial cells: Live (negative for live/dead dye) CD45-EpCAM+CD31-
Human fibroblasts: Live (negative for live/dead dye) CD45-EpCAM-CD31-Podoplanin+CD90+

☒ Tick this box to confirm that a figure exemplifying the gating strategy is provided in the Supplementary Information.

Magnetic resonance imaging

Experimental design

Design type: *Indicate task or resting state; event-related or block design.*

Design specifications: *Specify the number of blocks, trials or experimental units per session and/or subject, and specify the length of each trial or block (if trials are blocked) and interval between trials.*

Behavioral performance measures: *State number and/or type of variables recorded (e.g. correct button press, response time) and what statistics were used to establish that the subjects were performing the task as expected (e.g. mean, range, and/or standard deviation across subjects).*

Acquisition

Imaging type(s): *Specify: functional, structural, diffusion, perfusion.*

Field strength: *Specify in Tesla*

Sequence & imaging parameters: *Specify the pulse sequence type (gradient echo, spin echo, etc.), imaging type (EPI, spiral, etc.), field of view, matrix size, slice thickness, orientation and TE/TR/flip angle.*

Area of acquisition: *State whether a whole brain scan was used OR define the area of acquisition, describing how the region was determined.*

Diffusion MRI ☐ Used ☐ Not used

Preprocessing

Preprocessing software: *Provide detail on software version and revision number and on specific parameters (model/functions, brain extraction, segmentation, smoothing kernel size, etc.).*

Normalization: *If data were normalized/standardized, describe the approach(es): specify linear or non-linear and define image types used for transformation OR indicate that data were not normalized and explain rationale for lack of normalization.*

Normalization template: *Describe the template used for normalization/transformation, specifying subject space or group standardized space (e.g. original Talairach, MNI305, ICBM152) OR indicate that the data were not normalized.*

Noise and artifact removal: *Describe your procedure(s) for artifact and structured noise removal, specifying motion parameters, tissue signals and physiological signals (heart rate, respiration).*

Volume censoring: *Define your software and/or method and criteria for volume censoring, and state the extent of such censoring.*

Statistical modeling & inference

Model type and settings: *Specify type (mass univariate, multivariate, RSA, predictive, etc.) and describe essential details of the model at the first and second levels (e.g. fixed, random or mixed effects; drift or auto-correlation).*

Effect(s) tested: *Define precise effect in terms of the task or stimulus conditions instead of psychological concepts and indicate whether ANOVA or factorial designs were used.*

Specify type of analysis: ☐ Whole brain ☐ ROI-based ☐ Both

Statistic type for inference (See Eklund et al. 2016): *Specify voxel-wise or cluster-wise and report all relevant parameters for cluster-wise methods.*

Correction: *Describe the type of correction and how it is obtained for multiple comparisons (e.g. FWE, FDR, permutation or Monte Carlo).*

Models & analysis

n/a	Involved in the study
☐	☐ Functional and/or effective connectivity
☐	☐ Graph analysis
☐	☐ Multivariate modeling or predictive analysis

Functional and/or effective connectivity	*Report the measures of dependence used and the model details (e.g. Pearson correlation, partial correlation, mutual information).*
Graph analysis	*Report the dependent variable and connectivity measure, specifying weighted graph or binarized graph, subject- or group-level, and the global and/or node summaries used (e.g. clustering coefficient, efficiency, etc.).*
Multivariate modeling and predictive analysis	*Specify independent variables, features extraction and dimension reduction, model, training and evaluation metrics.*
