# Supplementary material for: The dual roles of uterine monocytes in regulation of tissue homeostasis throughout reproductive cycles in health and disease
Source: Nat Immunol. 2026 Sep 8;27(10):2058–73. doi: 10.1038/s41590-026-02651-y (PMC13630565; doi:10.1038/s41590-026-02651-y)
Supplement: Supplementary file 3 [file 41590_2026_2651_MOESM3_ESM.pdf]

nature portfolio

Peer Review File

The dual roles of uterine monocytes in regulation of tissue homeostasis throughout reproductive cycles in health and disease

Corresponding Author: Dr Elizabeth Mann

Version 0:

Reviewer comments:

Reviewer #1

(Remarks to the Author)
The manuscript by Shahzad studies the influence of monocytes and macrophages over various aspects of uterine biology in both mice and humans. This is an important but understudied area. In general, however, the manuscript was largely descriptive and the perturbational experiments performed in mice were not taken far enough to achieve significant mechanistic insight. We were left largely with a relatively superficial first-tier impression of some phenotypes. In addition, many of the experiments did not use appropriate methodologies. Specific concerns:
1. The connection between monocytes and uterine epithelial cell apoptosis is not substantiated. While not explicitly stated this way, I came away reading the key paragraph on page 7 with the sense that the authors are arguing that macrophages directly kill uterine epithelial cells through a Gzmb/perforin-dependent mechanism. To make this point, there needs at least to be an analysis of Gzmb or perforin KO mice, and a macrophage-specific conditional knockout to make the point conclusively. The idea that uterine macrophages express GzmB and perforin is rather unusual, so this should be thoroughly substantiated in its own right. Please show representative flow plots for Gzmb and Perforin.
2. Throughout the manuscript (Fig. 4G, 5C, 5D, 6B-G), the authors present various cell numbers as percentages of either CD45- or CD45+ cells. This is inappropriate, since a change in the prevalence of one kind of cell type (e.g. fibroblasts) automatically will change the percentage of other cell types, thus obscuring primary from secondary effects. For example, the increase in % EpCAM+ cells of CD45- cells in Ccr2 KO mice (Fig. 4G) could be due to CCR2 deficiency decreasing the number of fibroblasts as its primary effect. There needs to be some assessment of actual tissue density.
3. Collagen “area” is not a robust way of quantitating collagen deposition, especially as a single experimental modality. Please show hydroxyproline content or some other normalizable measure.
4. There is no justification presented for why Thy1 discriminates myofibroblasts from activated fibroblasts.
5. Which fibroblast subset(s) correspond to myometrial versus endometrial fibroblasts? The fluctuation in fibroblast subtype proportions across the estrous cycle could be explained by differences in the relative growth of these two tissue layers.
6. The key data of increased collagen “area” (see above) in older mice (Fig. 6A) is every subtle and required large sample sizes to achieve a minimal level of significance. The authors should also adopt a uniform way to orient and section their tissue blocks of mouse uteri. The varied orientation across samples makes rigorous interpretation impossible.
7. The large sample sizes in Fig. 6B-G needed to achieve frequently minimal levels of significance suggest that biological effects are very small.
8. The lack of a difference in Fig. 6H undercuts the authors’ primary hypothesis that monocytes and macrophages play a key role in uterine fibrosis. That the collagen appeared “disorganized and inconsistent” is not convincing.
9. Macrophages are known to regulate ovarian functon(old papers by Pollard). The authors need to address the possibility that the uterine phenotypes seen in Ccr2 KO mice are due to dysregulated sex steroid hormone production. Also please address the possibility that age-related changes in macrophage dynamics are due to altered hormonal cycling.
10. The manuscript lacks data showing that Ccr2 deficiency affects reproductive outcomes. To the contrary, Menzies et al. (BOR 2012) showed no effect on pregnancy outcomes, while Ccr2 KO mice have not been described to be subfertile. This reduces the significance of the study.
11. Page 10: The authors present their human data as novel. However, it has been known for many decades that uterine fibroblast and epithelial cell numbers change with the menstrual cycle (in fact, that is partially the very essence of the menstrual cycle). In addition, uterine fibroblasts proliferate in the proliferative phase (by definition), not the secretory phase. Something is awry here.
Minor:
1. Page 4, “where an inflammatory environment is required for embryo implantation.” I am not aware of anybody truly showing that this is the case. Please rephrase or provide are reference.
2. Page 4, “equivalent to that seen in the intestine”. I do not see this. Figure 1d shows different replenishment at 8 wks.

3. Page 5. The loss of uterine macrophages in Ccr2-/- mice is already known. Also, show representative flow plots here.
4. Page 6. Gene names should be capitalized; SIRPA not Sipra.
5. Page 7 refers to "glandular" epithelium, but the images mainly show the luminal epithelium
6. Page 8: the smooth muscle 3 cells are probably pericytes since they express Rgs5.
7. Page 9: "Csf1 [is] required for monocyte differentiation into macrophages" greatly oversimplifies the function of Csf1.
8. The Figure 1 legend does not correspond to the panels in the figure.

Reviewer #2

(Remarks to the Author)
In their manuscript "The dual roles of uterine monocytes in regulation of tissue integrity throughout reproductive cycles", Shahzad et al. describe their important findings regarding monocyte and macrophage populations in the cycling uterus. They provide in depth analysis of the different populations of mononuclear phagocytes across the different cycle stages of the uterus and provide data supporting the notion that the different populations might differ substantially in their contributions to healthy tissue maintenance. The authors provide mouse data and human data side-by-side, even including those from patients suffering from Asherman's Disease, further showing the high relevance and broad applicability of their study. This is an important and extensive study that provides a much-needed basis to further understanding of an under-studied tissue with major health implications for women.

I have the following general comments on the current form of the manuscript:
1) The authors define major populations of mononuclear phagocytes by RNA-seq and follow up on the monocytes, Mac1 and Mac2 by FACS. This is very useful and in many cases the authors provide the needed depth across different cycles. This is however not always the case, leading to some ambiguity regarding the stability of the markers used. For example, CD24 was identified as declining from monocytes to Mac1 to Mac2, but a critical assessment regarding CD11c and CD206 stability (used to subset the populations) seems to be missing. The stability of these markers (against others) should be tested across the different stages of the menstrual cycle. Along those lines, can the authors provide more information on the cycle stage underlying their RNAseq data? The authors seem to have stage specific RNAseq data for their epithelial/stromal data – can this be shown individually, for example for Figure 5B. I assume this data is not available for the analysis shown in Figure 2 and understand that providing this might be challenging.
2) The authors suggest that both Mac1 and Mac2 derive from monocytes, and according to their RNAseq data, they suggest that the two populations are derived independently. Based on BM chimera data, kinetics, and Ki67 staining, the authors suggest that Mac2 are replenished slower and proliferate in situ. Given the focus of the study, it would be good to gain more insight into why and when monocytes yield different macrophage populations. Is the idea that monocytes develop into closely related pro-inflammatory Mac1 at the height of their recruitment magnitude, which coincides with tissue inflammation and that Mac2 develop from monocytes that are recruited at different cycles, which require repair? This aspect is also important to properly interpret the CCR2 KO data, for which it is unclear in the current manuscript at which cycle stage the data was from, and whether the impact of CCR2 deficiency on Mac1/Mac2 ratios differs across different stages.

In addition to above points, following concrete comments should be addressed:
- Figure 1B: The data graphs for % vs cell numbers are extremely similar. This might be true, but can the authors double check that the data is accurate? It seems hard to believe that the total cell number so closely reflects percentage after all the necessary tissue digests etc
- Fig. 1D, E: In the text, the authors write that the replenishment of uterine macs closely mimics that of the colon, which really is not true for the 8 week timepoint. In addition: Why is the WT control between 1D and 1E so different? And: The legends do not fit the figure
- Fig. 2G: FMOs or isotype controls are missing. Maybe MFI would be better for all markers as there is no bi-model distribution, and the cut-off seems arbitrary?
- Fig.3B: Monocytes are Mac1s do not seem different across the PCA. Can the authors comment on that?
- Fig.5E-H and accompanying results: The authors make strong claims regarding the interactions between monocytes and fibroblasts, but in the currently depicted form, this seems somewhat biased, as they are only showing the interactions from monocytes and no comparisons to the other subsets. The text describes this somewhat, but it might be better to provide the data in the figure. In its current form, I find the notion surrounding monocyte and their role in collagen regulation somewhat overstated, especially since CCR2 deficiency should affect all subsets according to their shared monocyte dependency as suggested by the authors.
- Related to Figure 6: It is not very clear how the different stages of the cycle were defined in the mice, as the authors only refer to a previous publication. Is the method suited for young and old mice alike? Are there any differences?
- Introduction: The general immunology audience might need a slightly more extensive explanation of the different cycles, when what happens and what is known about macrophages in the uterus to date. As is, the introduction is very brief and does not provide the necessary foundation in my opinion
- Histology: Some panels are small, and the stain is hard to see – maybe zooming into some picture would be useful? Some size bars are not readable. For quantifications of absolute numbers, an explanation is missing (cells with field of view?)
- Data: Information on how many times individual experiments were performed is missing, so there is a potential need for repeats for some data panels.
- The manuscript should be carefully checked for typos and the correct links to data panels

Reviewer #3

(Remarks to the Author)
The author's have undertaken a detailed study of the role of infiltrating monocytes in mouse and human endometrium across the estrus and menstrual cycle, respectively, using flow cytometric analysis involving multiple surface markers and clearly described gating strategies, scRNAseq of mouse uteri, transgenic mice (Ccr2-/- mice), hyperion mass cytometry of human endometrium and computational approaches. They demonstrated that monocyte proportions varied dynamically over the reproductive cycle in mice and women and that they contributed to endometrial remodelling by inducing epithelial apoptosis. Endometrial monocytes and their macrophage derivatives also influence extracellular matrix remodelling, which they suggest differs between monocytes and Mac2s. Finally, they assess the relative roles of monocytes and Macs in ECM in endometrial fibrosis in aging mice and while they demonstrate alterations in cell types associated with fibrosis and the monocyte and macrophage populations in Ccr2-/- mice, the amount of collagen is not different to WT and the "disorganised collagen" appearance is not convincing given the overproduction of endometrial glands in this model. Asherman's syndrome characterised by replacement of endometrium with fibrotic tissue was shown to contain fibrotic tissue (excess collagen), surgery did not improve this, not surprising as fibrosis often recurs in this condition.
Strengths of the manuscript include clarity of the data presented in figures, use of pre-enriched myeloid cells for the scRNAseq study of mouse endometrium, the use of state-of-the-art technologies to identify the mechanisms involved as they undertook a detailed investigation of monocyte function in human and mouse endometrium. They provide new data on their role in endometrial function over the menstrual cycle (albeit needs some revision) and in fibrotic conditions of a generally neglected tissue which has major health implications for very large numbers of women globally.
Weaknesses include
1. There is a fundamental issue in the manuscript in equating endometrial changes in mouse proestrus and human early secretory stages of their reproductive cycles which is not correct and needs addressing. Ovulation occurs in humans in the interval phase at the end of the proliferative phase (circulating estrogen levels rise and dominate during this stage) and not in the early secretory stage, which occurs after ovulation, when progesterone levels begin to rise and endometrial cell proliferation rates substantially reduce. The early secretory stage is observed histologically by epithelial nuclear movement towards the apical lumen appearing as subnuclear vacuoles, due to progesterone-induced secretion of glycogen. In mice, ovulation occurs in the estrus phase and proestrus is most similar to the human proliferative phase where estrogen levels are rising. Metestrus is most equivalent to the human early secretary and mid secretory phase characterised by both estrogen and progesterone. Progesterone is still present in the diestrus and most like the human late secretory phase (Wood GA et al Reprod (2007; Ajayi & Akhigbe PMID: 32190339). This means the peak monocyte infiltration in mice and humans are at functionally different cycle stages (Fig 1), prior to ovulation in mice and following ovulation in humans. The graphs presented in Fig 1A should be replotted to account for these differences (proestrus as the first bar) and to assist data interpretation, clearly indicating the histological criteria used for staging the human samples as the "next predicted menstrual period" can be inaccurate in normal women with variable cycle lengths, and more so for Asherman's syndrome patients. Revision of the text in the Results and Discussion will also be required.
2. A Methodological issue with the immunofluorescent images from the Hyperion imaging system as there are some immunostaining is not representative using traditional IF techniques and of known endometrial biology. More detail is needed on how the Panels were established and verified for accuracy. Some high power images would assist showing where immune cells are assessed and maybe showing controls used when establishing the panels in Ext data figures.
3. Statistical analysis was well described and appropriate and while detailed in the Figure legends, there was no indication of what the bars or data points were. All appeared to be plotted as means and ?SD or ?SEM, but for non-parametric analyses, medians with interquartile range should be plotted as this is what is statistically different. This will alter how the authors describe their data.
Specific Issues
4. While the authors clearly show their gating strategy for distinguishing monocytes from macrophages, it is not clear that they actually achieve this (Ext Fig 1) as some mouse monocytes are Ly6C+ and/or MHC-II+ and some human monocytes are CCR2+. As the authors show, these expressions can change in different stages of the menstrual cycle, which they investigate further throughout the manuscript. Why were additional markers not considered eg F4/80 (mouse), CD16 (human)? However, later in the manuscript, they have used additional markers based on their findings from the single cell data.
5. Fig 1 – the figure legends do not match the panels correctly and as described in the text.
p3, 2nd para –peak monocyte influx in mice is in the pre-ovulation stage proestrus phase (estrogen dominant, equivalent to late proliferative in human), but in humans this occurs post ovulation in the early secretory stage.
6. Extended data Fig 3D does not have a figure legend.
7. P5 middle of bottom para – should be Fig 1a rather than 1c. 7 lines from bottom, Fig1a rather than 1b. Should the last line be Fig 2g?
8. P6 – top para – which stage of the estrus cycle was the experiment shown in ext data Fig 4a undertaken, as Lys6C varies in monocytes and macrophages across the estrus cycle.
9. P 6 2nd para – reword the sentence commencing "In support of less recent turnover of Mac2" to clarify its meaning. Delete '"monocytes and" in sentence describing Fig 3f – only Mac1 and Mac2 are shown in this panel.
10. P6 2nd to last line – refer to Fig 3e
11. Page 7, 1st para – there is a week before the window of implantation occurs in humans (ie mid secretory stage), so the monocytes must differentiate as indicated by Fig 3g, perhaps into Mac2?
12. P 7 2nd para – line 2 – should be Fig 2d – please check all the text throughout the manuscript for accuracy of the referring to Fig panels.
What are the "immature progeny of monocytes"?
13. Fig 4 – Panel D - please quantify the ClC3+ epithelial cells for the estrus cycle stages as done for Ccr2-/- mice. Panel E needs scale bars (magnification looks different between the 2 images) and a more representative image of wt estrus endometrium which should show some gland profiles to match the data shown for estrus in Panel F. Panel G, why are there

2 types of dot points in the graph?
Panel H –Granzyme B is difficult to see visually – please include the control Ab Panels used to generate the set of antibodies for this Fig. In the Fig Legend, indicate the stage of the menstrual cycle forthis section. Maybe also include a late proliferative stage endometrium which is more comparable to mouse proestrus stage. And more examples of HLA-DR+ macrophages clustered in close proximity of human endometrial gland profiles could also be shown in an Ext Data figure.
14. Fig 5 Panel A Only pro-estrus is shown. To demonstrate that more collagen is deposited at this stage, all stages of the estrus cycle should be shown in Ccr2-/- mice. p 8, clarify what a "network" of various endometrial cells is in the scRNA seq data?
Fig 5c –Why are there different number of replicates for some of the cycle stages as the same flow plots are used for determining activated fibroblasts and myofibroblasts? Medians should be plotted for the myofibroblast graph for panel c and d. Has the analysis conducted for Panels f-h been assessed for interactions with Mac2 as the CellChat suggests they are similar (Panel e)? This would strengthen the interpretation that incoming monocytes are more likely to interact with fibroblasts than Mac2s. Similarly for ext Fig 7, what do myofibroblasts interactions with monocytes and Mac2 show? Also monocytes and Mac2 seem to show very similar interaction probabilities in this fig.
15. Fig 6 –were equal numbers of mice used from each cycle stage comparison between young and older mice to produce the graphs in all the various panels as the staining in the images show quite different staining intensities at different stages and was more easily observed in the images showing better examples of longitudinal sections. This needs to be included in the Figure legend. Where non-parametric statistical tests were done, medians and IQRs should be plotted. The tissue block used for the aged estrus uterus is not a good example of a longitudinal section in both Fig 6 and ext Fig 8. The older CCR2-/- mice uteri look like they still have many glands which more likely contributes to the disorganised look of Sirius red staining rather than a real effect .
16. P9 last para – thinner endometrium with fewer gland would be a more accurate term than "thinner glandular layer."
17. Suppl Table 1 gives a good summary of patient demographics, including for Asherman's syndrome patients. However, the severity of the disorder and a menstruation history is very important, but not included. How many Asherman's patients were menstruating and how regular were their cycles and how heavy were their periods. Severe Asherman's women do not menstruate and those that do often have scanty menstrual flow. Is it possible to get this information from the pathology reports? Please note that a total of n=24 samples were obtained in the Control proliferative stage but the numbers add to n=25 for ethnicity.
18. Fig 7 Panel A – How were the Control panels established for these images as there is intraluminal collagen staining artifact in the Mid-secretory image and the number of proliferating stromal cells is high in the mid and late secretory endometrium and does not match the graphed data. Are medians plotted in the figure? Ext Fig 9b shows graphs from the epithelial and stromal cell flow plots but they are not representative of human endometrial histology. The number of glands (epithelial cells) increases across the cycle and generally comprises up to >30% and the relative proportion of stromal cells decreases. It would appear that the dissociation protocol has not worked sufficiently and particularly in the mid and late secretory stages when the epithelial cells are post-mitotic and difficult to dissociate without using very strong enzyme solutions. P10 top para – the sentence suggesting that stromal cells increase across the healthy menstrual cycle is incorrect – most are observed in the proliferative stage and least in the secretory stages and vice-versa for the epithelial cells and should be reflected in the flow cytometry as glands as epithelial cells proliferate at a much higher rate than stromal cells. Very few Ki-67+ cells are seen in glands and stroma in the late secretory stage.
19. Fig 7- Panel C shows too many Ki-67+ "stromal nuclei" suggesting artifact in the stroma of the post surgery group. More details are required how the panel was established for this set of images. There are 2 panel Ds, please correct. Panel E - the post surgery pro-inflammatory Mac1s and monocytes also appear clustered as in the fibrosis section and is reflected in Panel F where there is no difference between fibrosis and post surgery for these cells, similarly for Mac2s. However all 3 cell types are significantly different in the diseased and surgically treated tissues compared to the controls. This needs to be made clearer in the text P10 last para. The broad spectrum of patient presentation in intra uterine adhesions/Ashermans syndrome makes it difficult to see differences, particularly if the patient menstrual data is not known.
Discussion
20. The authors point to the lack of studies demonstrating a role for monocytes in endometrial function and that their roles have not been identified in recent high profile single cell studies in mouse or human reproductive tract. This is true in part but also an over a misinterpretation of the literature. The problem is the overlapping markers for monocytes and macrophages make this difficult to study as the authors have mentiond. The authors' study does provide a very detailed study of the role of monocytes in endometrium. Novel is their use of pre-enriching the myeloid cell populations in their scRNAseq study. However, they did not compare their findings with that in the Bunis et al paper (ref 37) which did include a set of control women and did report the monocytes as a separate population, demonstrating they increase in the mid secretory stage, compared to earlier cycle stages.
21. Surprisingly the study failed to identify well characterised peak of monocyte/myeloid infiltration in the late secretory phase in human endometrium, which contribute in a major way to the destruction of the functionalis layer for shedding into the menstrual blood and its subsequent massive regeneration during the proliferative stage (ref 38, Salamonsen et al . PMID: 34486650 and refs therein). This fundamental difference between mouse and human was not mentioned. Additionally, technical issues (see pt 18 above) failed to reflect the relative numbers of EpCAM+ epithelial cells in human endometrium during the secretory stages, weakening enthusiasm for the importance of this study.
22. Para 3 needs to be rewritten in the context that proestrus in mice is equivalent to the proliferative phase of the cycle and while the authors' showed infiltration in the early secretory stage of the cycle in human samples, this is not the peak of glandular epithelial proliferation, which occurs in the late proliferative stage and rapidly slows following ovulation due to the inhibitory effects of progesterone. Both references 38 and 39 referred to in this paragraph demonstrate that apoptosis in human endometrium mainly occurs in from late secretory to early proliferative stages in controls, and also include the role of TNF. An additional ref that should be discussed is Santamaria et al Nat Commun PMID: 37735465, a scRNA seq study of endometrium from Asherman's syndrome patients.
23. The Abstract will need revision to account for the above (point 22) and other changes required for this manuscript.

24. Other important concepts not included in the Discussion are the role of the sex steroid hormones, estrogen and progesterone which overall regulate the cellular and molecular changes across the estrus and menstrual cycle. Monocytes and macrophages variously express nuclear estrogen receptor isoforms and progesterone receptors, including membrane bound GPCR (Pelekanou V et al 2016 PMID: 26394816; Murpy et al 2009 PMID: 19440537). Were these identified in the scRNAseq work? And what is their role in inducing the influx of monocytes into endometrium?
25. In summary, this is an interesting manuscript, well written and presented, that may contribute novel information on the role of monocytes in endometrial biology and endometrial diseases, but needs revisions to determine how significant the findings will be. It should be noted that there is a dearth of knowledge on endometrial and menstrual disorders which have massive effects on large numbers of women and this manuscript may well contribute new knowledge needed to further understand these conditions and stimulate further research that would benefit these women.

Version 1:

Reviewer comments:

Reviewer #1

(Remarks to the Author)
The revised manuscript, even with the new data, remains largely a survey of monocyte and macrophage dynamics in the mouse uterus, with correlative data from normal humans and humans with Asherman syndrome. Its functional assessment of the importance of these cells in reproductive outcomes remains superficial and diffuse, with phenotypes (e.g. more collagen in Ccr2 KO mice) just described but not dissected. Other "mechanistic" experiments (a requirement for TGF-beta and IFNg in uterine macrophage/monocyte dynamics) are similarly superficial. There are some novel data, but these are relatively minor and do not push the reproductive biology nor the monocyte/macrophage fields forward in major ways. Continued and additional concerns:

The manuscript still states: "monocyte-induced epithelial cell apoptosis" (page 12) and "cytotoxic properties of uterine monocytes and macrophages" (page 43), and this section, with its emphasis on GzmB and perforin expression, still gives the misleading impression that macrophages are killing uterine epithelial cells. The requested GzmB and perforin KO experiments were not performed. In their rebuttal, the authors seem to be arguing that they decided to keep including the GzmB and perforin data because of its inherent interest, but that's not how it reads to me.

Certain key results have already been published in PMID: 21825019 (J. Exp. Med. 2011). This paper showed that mouse uterine macrophages divide into two subsets, are greatly depleted from uterus in Ccr2 KO mice indicating a monocytic origin, and make chemokines that likely recruit monocytes. And in contrast to the results in Fig. 7C, Ccr2 KO mice have previously been shown to have normal litter sizes (PMID: 22278981; coincidentally(?) also from a Glasgow group), as I previously noted. Neither paper is cited and the discrepancy with PMID: 22278981 is not commented on but should be.

There are inappropriate logical elisions. For example, while CCR2 deficiency apparently leads to breeding abnormalities as well as changes in the uterine ECM and epithelial cells, the manuscript implies that the breeding abnormalities are *due* to the uterine ECM and epithelial cell changes. This is not established, and many other possibilities exist (aside from the discrepancy between the breeding defect seen here and PMID: 22278981). Perhaps there is an ovulation defect.

Fig. 8A collagen area (resubmission) has different data for the older mice than that in the original (Fig. 6A).

Fig. 6F remains problematic. Why are all the endothelial cells massively expanded as well? How were absolute epithelial cell numbers determined? This is not a trivial endeavor unless precise amounts of tissues are run through the cytometer or counting beads were used. I do not see mention of this.

I'm not convinced from Fig. 6H and ED 9B the HLA-DR+ cells are the ones that express Granzyme B. The abutting of the gland seems only to be in one spot.

The justification not to assess estrogen levels only in diestrus and not across the entire estrous cycle is that diestrus is when there is the difference between progesterone levels in Ccr2 KO mice versus controls. This is not a compelling argument. Also, I was concerned not that altered hormones are changing monocyte/macrophage dynamics but rather that these altered dynamics were altering hormone levels. Which they appear to do at least for P4 in diestrus.

It remains problematic to score cell abundance as percentages of CD45- or CD45+ cells.

Collagen "area" remains an inappropriate measure of collagen content.

All of the Asherman data is correlative and observational, and interpretations are speculative.

Blurry figures (e.g. 7D, ED 8B, and many other places, but next to figures that are sharp so this is not a download issue), inconsistent formatting (e.g. ED 8F&H), inappropriately placed figures (ED 9D), ED 19 is not called out in the Results section. Multiple references are missing (the text just shows a "ref" placeholder). These errors raise concerns about the level of attention to detail.

The staining in Fig ED 9C looks artefactual.

Reviewer #2

(Remarks to the Author)
The authors have added a substantial amount of data and clarification to this new version of their manuscript. My concerns were addressed in a satisfactory manner.
One aspect that I would appreciate more clarity on is the differentiation of human monocytes into HLA-DR low and high cells. The gating strategy in extended figure 2 somewhat suggests that HLA-DR low monocytes might be excluded from the analysis shown in Figure 1. Figure 5 shows all in all much less of a fluctuation of monocytes across the cycle, even if gated on the HLA-DR low monocytes, which are supposed to be the ones changing in abundance. Can the authors explain this discrepancy?
Another aspect concerns the interpretation of the extensive expression changes of MerTK, CD86, and CD4 on monocytes across the cycle. They seem fairly absent during the proestrus, when monocytes are abundant, and very high in the diestrus, when there are almost no monocytes remaining. Is it still accurate to call these cells monocytes? The gating in Figure 1 A suggests that there are none, which would explain the data, potentially.
References are missing in 6 cases in the manuscript, where it reads '(ref)'.

Reviewer #3

(Remarks to the Author)
The authors have undertaken a thorough revision of their manuscript, undertaking further experiments, and in general have addressed all the points I raised. There are a couple of minor issues remaining or have arisen as a result that need to be addressed, although there is still some misunderstanding on differences between the cyclic changes in mouse and human endometrium.
1. Here are the differences and similarities of the estrus and menstrual cycles. The WOI occurs in fertile women in all ovulatory cycles during the mid-secretory stage when endometrial decidualisation spontaneously occurs, while there is no WOI in cycling mice as the ovulated oocytes need to be fertilised to induce endometrial stromal decidualisation. Thus the WOI occurs on day 4-5 of pregnancy in mice. It can be simulated experimentally by providing a decidualisation stimulus – oil droplet injection into the uterine horn or mating with a castrated male mouse (pseudopregnancy). This is why mice do not menstruate as decidualisation is a terminal differentiation of the human endometrium which needs to be removed to enable endometrial regeneration as a new cycle commences. The authors should highlight these differences and point out that the monocyte differentiation is a similar process in both humans and mice, despite the differences in cycle/pregnancy dynamics. Accordingly, the WOI cannot be used to cover both mouse and human in the Abstract as the manuscript currently stands. The authors would need to induce WOI in pseudopregnant mice to demonstrate the monocyte/Mac role in uterine remodelling during decidualisation to show they mimic the same role in human WOI. It would also still be useful to plot the cycle stages on the graphs as per convention to commence with proestrus.
2. In the Introduction (p3, 1st para) please add in the secretory differentiation of the glandular epithelial cells ie "…the endometrium undergoes cyclical episodes of proliferation (estrogen-dominated proliferative phase of the menstrual cycle) followed by differentiation of the epithelium into secretory cells and fibroblasts into decidual cells (progesterone-dominated)."
3. Fig 8A/Ext data 15A – "A thinner epithelial layer surrounding glands (glandular epithelium) etc" Are the authors describing the glandular epithelium which changes in height – highest/thickest during estrogen-dominant phase and lower in other phases, particularly low estrogen. Endometrial glands are lined with a single layer of pseudostratified columnar epithelium Could the authors be seeing lower epithelium in fewer glands in a thinner endometrium in older mice? Clarify the wording.
4. Fig 9 F, G. What does "case average" mean in the Y axis label – perhaps clarify in the Fig legend.
5. P 16 second to last sentence – Mac1s ae also sustained at a high level in the proliferative stage (Fig 10H) but '(Fig 10G)'.should also be referenced for the monocytes.
6. Discussion p 18 3rd para – the marked reduction of glandular epithelial proliferation is also driven by progesterone, and the secondary effect could also be due to the infiltration of monocytes that induce apoptosis. This may be of greater importance in mice as they remodel the endometrium rather than shedding it as in humans.
7. Discussion p 19. Was ESR2 found in your data set on monocytes and/or Mac1s?
8. Discussion p 20 1st para – delete "(and thus epithelial layers)"
9. There are numerous places in the new text showing "(ref)". Please insert the relevant references.

Version 2:

Reviewer comments:

Reviewer #2

(Remarks to the Author)
I had only few remaining questions/comments following the first resubmission of this manuscript, and those are now all answered. I believe that this manuscript provides a substantial advancement, as the current knowledge provided by the literature regarding uterine monocytes and macrophage abundancies and roles across hormonal cycles is frustratingly absent. The study fills those gaping holes by providing phenotypic and functional data across human and mouse tissue. I commend the authors on such a thorough and holistic piece of work.

Reviewer #3

(Remarks to the Author)
The authors have addressed my residual concerns thereby improving the accuracy of their manuscript, particularly in relation to the differences between mouse and human endometrial cycling.

Open Access This Peer Review File is licensed under a Creative Commons Attribution 4.0 International License, which permits use, sharing, adaptation, distribution and reproduction in any medium or format, as long as you give appropriate credit to the original author(s) and the source, provide a link to the Creative Commons license, and indicate if changes were made.
In cases where reviewers are anonymous, credit should be given to 'Anonymous Referee' and the source.
The images or other third party material in this Peer Review File are included in the article's Creative Commons license, unless indicated otherwise in a credit line to the material. If material is not included in the article's Creative Commons license and your intended use is not permitted by statutory regulation or exceeds the permitted use, you will need to obtain permission directly from the copyright holder.
To view a copy of this license, visit https://creativecommons.org/licenses/by/4.0/

We thank all reviewers for their comments and have now made substantial revisions that we believe has improved the quality of this paper.

Reviewer #1

(Remarks to the Author)
The manuscript by Shahzad studies the influence of monocytes and macrophages over various aspects of uterine biology in both mice and humans. This is an important but understudied area. In general, however, the manuscript was largely descriptive and the perturbational experiments performed in mice were not taken far enough to achieve significant mechanistic insight. We were left largely with a relatively superficial first-tier impression of some phenotypes. In addition, many of the experiments did not use appropriate methodologies.

We have taken on board comments here describing our paper as descriptive and have now carried out additional functional experiments using manipulation of mouse models to provide further mechanistic insight. This has involved using our scRNAseq dataset to identify target molecules expressed by monocytes and macrophage subsets that may facilitate their differentiation into either pro-inflammatory Mac1s or pro-repair Mac2s.

Since Mac2s selectively expressed high levels of TGFβR1 and TGFβR2 alongside many genes involved in TGFβ response pathways (Extended data 4A), these experiments included the use of a blocking antibody to TGFβ which affected overall macrophage differentiation and in particular the development and proliferation of Mac2s (Figure 3G, H and I). In addition, we investigated the role of IFNγ using Ifngr$^{-/-}$ mice which exhibited skewing of their macrophage subsets, with reduced Mac1s (Figure 3J). Cytokine production in Ccr2$^{-/-}$ mice was comparable with the wild type (Extended data 7H) in both subsets.

Critically, we now demonstrate functional consequences for the disruption of tissue integrity in the uterus of Ccr2$^{-/-}$ mice, using historical records from our collaborators at the University of Glasgow who are breeding both Ccr2$^{-/-}$ and wildtype mice in house (we are breeding heterozygotes so it was not possible to analyse data from our own colonies). Ccr2$^{-/-}$ mice have significantly fewer live pups per litter than wildtype mice (Figure 7C).

In addition, we have now assessed the turnover of our specific macrophage subsets (Mac1 and Mac2) by carrying out additional bone marrow chimera experiments, showing that Mac1s are replenished more quickly than Mac2s (Figure 3A), fitting with our scRNAseq trajectory analysis in mice and RNAseq data in humans indicating Mac1s are more closely related to monocytes than Mac2s.

Human monocyte functions have now been explored further, splitting total monocytes into two distinct subsets: HLA-DR^{hi} and HLA-DR^{lo}. The HLA-DR^{lo} subset is more inflammatory with regards to cytokine production (Figure 5D and E), and accounts for the fluctuations in monocyte numbers through the menstrual cycle, whereas the HLA-DR^{hi} subset remains more constant (Figure 5B and C).

We had to start this project with extensive in-depth phenotyping because so little was known about the presence and role of monocytes in the female reproductive tract, and it had not been shown that macrophages in the uterus are turned over from recruited monocytes. Therefore although we have now added crucial functional data to this study, we would argue that describing myeloid cell biology and ontogeny within the uterus in depth is fundamental and important for advancing the field as discussed by reviewers 2 and 3.

Specific concerns:
1. The connection between monocytes and uterine epithelial cell apoptosis is not substantiated. While not explicitly stated this way, I came away reading the key paragraph on page 7 with the sense that the authors are arguing that macrophages directly kill uterine epithelial cells through a Gzmb/perforin-dependent mechanism. To make this point, there needs at least to be an analysis of Gzmb or perforin KO mice, and a macrophage-specific conditional knockout to make the point conclusively. The idea that uterine macrophages express GzmB and perforin is rather unusual, so this should be thoroughly substantiated in its own right. Please show representative flow plots for Gzmb and Perforin.

Representative flow cytometry plots have now been included for mice (Extended data 8D) and humans (Extended data 9A) re expression of perforin and Gzmb. In addition, we also now show immunofluorescence microscopy imaging of Gzmb (Extended data 9C) and we have used alternative Hyperion images demonstrating more clearly that HLA-DR$^+$ cells next to the epithelium express Gzmb (Figure 6H), with individual expression of PanCK, Gzmb and HLA-DR shown in Extended data 9B.

In addition to perforin and Gzmb, monocytes/Mac1s in both mice and humans expressed many other genes involved in cell killing pathways, with cell killing/programmed cell death being one of the top hits in RNAseq datasets from both species in gene pathway analyses (Figures 4F and 6A). We have shown in both mice and humans that monocytes in the uterus constitutively produce high levels of TNFα (Figure 2K (mouse) and 5D (human) with no ex vivo stimulation. As mentioned in the discussion, TNFα has been proposed to play a critical role in epithelial apoptosis in the uterus. While we are not sure of the exact mechanism by which the monocytes contribute to epithelial cell apoptosis, our data showing that epithelial levels of cleaved caspase 3 are diminished in monocyte deficient mice (Fig 6C/D and Extended data 8E/G), alongside expansion of epithelial cells and abnormal/dense epithelial architecture (Figure 6E,F) constitutes strong evidence of a role for monocytes in regulating epithelial cell apoptosis in the uterus. Gzmb-expressing macrophages also cluster next to epithelium in our human samples as assessed by Hyperion (Figure 6H).

In our discussion we point to other instances of monocytes/macrophages expressing Granzyme B, as in inflammatory diseases including rheumatoid arthritis and atherosclerosis (PMID 17604848), and a cytotoxic phenotype has been described for monocytes in response to inflammatory signalling pathways in certain disease settings (toxoplasmosis, kidney graft rejection, anti-tumour responses). Thus it has been known for a long time that monocytes have the capacity to be cytotoxic. What is interesting here

is that this occurs in the uterus in the steady state, rather than in an inflammatory/disease setting.

Given financial and time restraints, we have not included additional data regarding Gzmb or perforin KOs (we currently only have access to sperm) or macrophage-specific KOs. This was also decided because, as discussed above, we are not sure it is the pathway by which monocytes exert cytotoxic effects on epithelial cells. We have made the point about perforin and granzyme expression in the manuscript because, as the reviewer pointed out, this is an unusual feature of monocytes (whereas TNF production is not unusual).

2. Throughout the manuscript (Fig. 4G, 5C, 5D, 6B-G), the authors present various cell numbers as percentages of either CD45- or CD45+ cells. This is inappropriate, since a change in the prevalence of one kind of cell type (e.g. fibroblasts) automatically will change the percentage of other cell types, thus obscuring primary from secondary effects. For example, the increase in % EpCAM+ cells of CD45- cells in Ccr2 KO mice (Fig. 4G) could be due to CCR2 deficiency decreasing the number of fibroblasts as its primary effect. There needs to be some assessment of actual tissue density.

We have now presented absolute numbers as well as proportions for flow cytometry data (either in the main figures themselves or Extended data). We agree this is important and this has been highlighted, in particular in Figure 10 where the proportions of Mac1s go up and Mac2 go down substantially in uterine fibrosis (Asherman Syndrome). However additional assessment of absolute numbers now indicate Mac2s are not lost, they in fact remain constant against a large increase in Mac1 numbers (Figure 10E and F). The expansion of EpCAM+ cells in Ccr2$^{-/-}$ mice is true (now Figure 6F) as shown by absolute numbers.

3. Collagen “area” is not a robust way of quantitating collagen deposition, especially as a single experimental modality. Please show hydroxyproline content or some other normalizable measure.

Hydroxyproline assays have now been carried out in Ccr2$^{-/-}$ mice (Figure 7b) and older wildtype mice (Figure 8b) confirming in both cases that there is increased collagen deposition.

4. There is no justification presented for why Thy1 discriminates myofibroblasts from activated fibroblasts.

Apologies if this was not made clear in the manuscript. Our single cell RNAseq data of murine CD45^{-} cells demonstrate clear identification of all stromal cells and fibroblasts by expression of Pdgfra, distinguishing them from smooth muscle, endothelial and epithelial cells. Within the Pdgfra+ stromal cells/fibroblasts, we have sub-characterised myofibroblasts as cells expressing Acta2. Within these total stromal cells/fibroblasts, these Acta+ cells are the only subset that do not express Thy1. This can all be seen clearly

in Figure 7D, with further clarification of the markers used for surface staining by flow cytometry based on gene expression in Extended data 11A and B). In addition to their expression of Acta2, the subset we have called myofibroblasts (negative for Thy1) also express very high levels of Lumican (this can also be seen in Figure 7D) which other single cell datasets show is specific for myofibroblasts in other mucosal tissues (eg lung).

5. Which fibroblast subset(s) correspond to myometrial versus endometrial fibroblasts? The fluctuation in fibroblast subtype proportions across the estrous cycle could be explained by differences in the relative growth of these two tissue layers.

For the mouse data, we agree it is hard to distinguish contributions of myometrial versus endometrial cells due to the digest of the whole uterus, thus we have referred to all mouse data as "uterine" rather than endometrial, unlike the human data. The fluctuations in fibroblast proportions in the murine uterus through the estrous cycle has now been validated with absolute numbers (Extended data 11C) and numbers are still lowest during proestrus, in particular for the myofibroblasts. Any changes in relative growth of myometrium should predominantly be due to smooth muscle. To this end we have tried to assess endometrial versus myometrial fibroblasts and in particular myofibroblasts with immunofluorescence microscopy to keep tissue structure intact. Although there was clear (but sparse) staining of αSMA in the endometrium likely representing myofibroblasts, obviously all the smooth muscle in the myometrium also expresses αSMA and so it was not possible to discriminate myofibroblasts. Pdgfrb staining indicated the presence of fibroblasts in the endometrium and myometrium and so it is difficult to assess which compartment the fluctuations were are seeing correspond to (this could well be both). However this may partially explain the difference in patterns of fluctuations between mice. These imaging data re mouse uterus have been attached here for reviewers rather than included in the manuscript (these may be added upon request however).

The key point that we are trying to make in the paper regarding fibroblast subsets in mice is the increased numbers of myofibroblasts in Ccr2$^{-/-}$ mice that develop spontaneous fibrosis, in particular during proestrus when numbers should be low (Figure 7F/G). Thus we needed to assess what happened to fibroblasts in WT mice across the cycle to be able to compare Ccr2$^{-/-}$ mice but the WT/cycle data are not fundamentally important to the key message.

The human data has been generated from endometrial biopsies and so definitely corresponds to endometrial fibroblasts.

6. The key data of increased collagen "area" (see above) in older mice (Fig. 6A) is every subtle and required large sample sizes to achieve a minimal level of significance. The authors should also adopt a uniform way to orient and section their tissue blocks of mouse uteri. The varied orientation across samples makes rigorous interpretation impossible.

We have now carried out hydroxyproline assays on the older versus young mice which confirmed excess collagen deposition (in much smaller numbers, as we used banked tissue that we already had available; Figure 8B).

We would like to note that uterine fibrosis occurring as a natural part of the ageing process in mice is a known phenomenon (PMID 38325365). Thus we are not describing this for the first time and our model may be more subtle because our mice are older (ie 9-12 months) rather than aged (18-24 months) so that they still have an estrous cycle, as we wanted to study the kinetics through the cycle of the immune cell subsets and CD45$^-$ cells. We are therefore using an already established model of uterine fibrosis to characterise the contribution of monocytes and macrophages to the chronically inflamed environment in the uterus of aged mice. This is important because our mouse data mirrors what we see in human uterine fibrosis (Asherman Syndrome) in terms of loss of epithelia, sustained increased in monocytes and inflammatory Mac1 macrophages.

We have also amended orientation and sectioning of our tissue blocks and have replaced the original images (Figure 8A and Extended data 15A).

7. The large sample sizes in Fig. 6B-G needed to achieve frequently minimal levels of significance suggest that biological effects are very small.

Cycle stages have been pooled here for clarity/space purposes – especially with the addition of absolute numbers meaning lots of space is needed for these figures. We did not need these numbers for statistical significance but numbers are high overall as we wanted enough mice in each stage to assess the kinetics of epithelial cells and monocyte/mac subsets through the cycle (NB these are now Figures 8C-H and Extended data 15B-G).

8. The lack of a difference in Fig. 6H undercuts the authors' primary hypothesis that monocytes and macrophages play a key role in uterine fibrosis. That the collagen appeared "disorganized and inconsistent" is not convincing.

We think that since the collagen deposition and level of fibrosis is already extensive in the older mice (wildtype), the phenotype in the Ccr2$^{-/-}$ mice may be too subtle to see a difference in total amount of collagen. We have now removed the Ccr2$^{-/-}$ data to avoid any confusion. The main point of this figure was to assess the contribution of CD45$^-$ cells, monocytes and macrophages to the fibrotic environment in mice and we think it is still important to make the point that there are losses of fluctuations in epithelial cells, fibroblasts and macrophages in the fibrotic uterine environment in mice. This is slightly unusual when compared to fibrosis in other tissues which usually show higher numbers of fibroblasts and dysregulated epithelial cells, but this makes sense in the uterus due to the drastically changing tissue environment, meaning the fluctuations themselves are critical for maintaining a "normal" structure.

Importantly these mouse data mirror our findings in human uterine fibrosis (Asherman Syndrome) re sustained loss of epithelia and high numbers of monocytes and inflammatory macrophages with a general suppression of tissue dynamics (reduced fluctuations in epithelial and endometrial thickness meaning the endometrium remains uniformly thin). Thus Asherman Syndrome patients with severe disease have limited menstruation.

For comparisons in Figure 9 and 10 (Asherman Syndrome), we used data from patients with who still have regular menstrual cycles (albeit light periods and an abnormally thin endometrium), and thus can be stratified into cycle stages. This is important so that we can compare them to healthy individuals at the corresponding stages. Furthermore, the post-surgery patients were all on estrogen treatment which mimics the proliferative phase to induce endometrial proliferation/thickening. Thus we needed the pre-surgery patients to be in the proliferative phase of the cycle in order to compare fibrosis patients pre- and post-surgery, as in Figures 9 and 10. Accordingly, we needed our healthy controls also to be in the proliferative phase. We have now added additional data including information from Asherman Syndrome patients in other cycle stages to show that regardless of cycle stage, high numbers of monocytes are sustained and thus the normal/homeostatic kinetics and rhythmicity seen in healthy controls is lost (Figure 10G).

9. Macrophages are known to regulate ovarian function(old papers by Pollard). The authors need to address the possibility that the uterine phenotypes seen in Ccr2 KO mice are due to dysregulated sex steroid hormone production. Also please address the possibility that age-related changes in macrophage dynamics are due to altered hormonal cycling.

This been discussed in the results where additional data have been added investigating circulating levels of progesterone (via mass spectrometry; Extended data 6K) in Ccr2$^{-/-}$ mice compared to wildtype, at different cycle stages. Levels were comparable aside from diestrus where they were higher in Ccr2$^{-/-}$ mice (Extended data 7I). An estradiol ELISA was carried out on wildtype and Ccr2$^{-/-}$ mice in diestrus (based on the progesterone results) and there were no significant differences although numbers were small (Extended data 7J). We don't think the alterations to monocyte and macrophage dynamics in uterine fibrosis in mice is due to altered hormonal cycling because of age, because the exact same dynamics occur in human endometrial fibrosis (patients are reproductive age) – with increased numbers of monocytes and Mac1s (Fig. 8F/G and Ext.data 15E/F for mice, and Fig.10 for humans).

10. The manuscript lacks data showing that Ccr2 deficiency affects reproductive outcomes. To the contrary, Menzies et al. (BOR 2012) showed no effect on pregnancy outcomes, while Ccr2 KO mice have not been described to be subfertile. This reduces the significance of the study.

We now have included additional data in the manuscript, demonstrating that there are significantly fewer live pups per litter in Ccr2$^{-/-}$ mice, as a reproductive outcome (Figure 7C). These are data received from the animal facility at the University of Glasgow who have been breeding Ccr2$^{-/-}$ mice alongside wildtypes for many years and are long term collaborators. We are breeding heterozygotes in Manchester so were unable to use data from our own colonies.

11. Page 10: The authors present their human data as novel. However, it has been known for many decades that uterine fibroblast and epithelial cell numbers change with the menstrual cycle (in fact, that is partially the very essence of the menstrual cycle). In addition, uterine fibroblasts proliferate in the proliferative phase (by definition), not the secretory phase. Something is awry here.

We agree that our assessment of human fibroblasts through the menstrual cycle by flow cytometry did not previously fit with what should be expected (i.e. proliferation of the endometrium in the proliferative phase). We have worked out this is likely due to stromal cells selectively dying quicker than CD45$^+$ cells in our digest protocol for flow cytometry, which is optimised for immune cells (this does not appear to be an issue with the mouse tissue digests). To resolve this, we have now excluded all flow cytometry data regarding human stromal cells/fibroblasts and instead have used analyses from our Hyperion experiments (Figures 9B and C) which does not involve any tissue digest (instead uses FFPE sections) to assess stromal cell fluctuations through the menstrual cycle. This now corresponds to what is expected, that is, stromal cells are highest during the proliferative phase. Although the glandular epithelial cells are highly proliferative in the proliferative phase of healthy individuals, the actual number of glands is much higher by the late secretory phase meaning epithelial cell numbers are highest at this point (Figure 9C).

The novelty of our human data outwith the context of disease lies in the monocyte and macrophage subsets, functions and their proximity to the epithelium. This is important in light of the mouse data we have indicating monocytes contribute to regulating epithelial cell apoptosis. We agree that fibroblast and epithelial cell dynamics have already been characterised, but we needed to assess this in our own hands as these data are essential for our comparison with Asherman Syndrome patients in whom we show epithelial cells are lost, as is evidence of epithelial cell proliferation (Ki67; Fig.9F/G and Ext.data 16b). Epithelial cells are restored with surgery, as expected to a degree, due to the mechanical clearance of adhesions/fibrotic tissue, however the key point we are making is in the fact that the immune dysfunction persists despite apparent restoration of anatomical structures and in particular, the consistently high numbers of inflammatory monocytes and Mac1 macrophages (Fig.10) in close proximity to the epithelium (Fig.9H-J) . We predict that the immune-mediated pathways may play a major role in the propensity of these adhesions to recur, as this happens in nearly all patients within weeks/months. Text has been amended in the manuscript to focus on the novelty regarding the monocytes and macrophages.

Minor:

1. Page 4, “where an inflammatory environment is required for embryo implantation.” I am not aware of anybody truly showing that this is the case. Please rephrase or provide are reference.

This has now been rephrased and additional references added.

2. Page 4, “equivalent to that seen in the intestine”. I do not see this. Figure 1d shows different replenishment at 8 wks.

This has also been rephrased to make the point that although total replenishment is slower than macrophages in the intestine, by 12-14 weeks the level of replenishment matches that of intestinal macrophages.

3. Page 5. The loss of uterine macrophages in Ccr2-/- mice is already known. Also, show representative flow plots here.

Monocytes are lost in the uterus of $Ccr2^{-/-}$ mice as expected, we just wanted to show that in our own experiments there are definitely no monocytes present in the uterus so that we could draw conclusions from the changes that occur in the absence of monocytes (Extended data 7B). There is an overall loss of macrophages as we have also shown (Extended data 7C). However it is not known that the macrophages that remain in $Ccr2^{-/-}$ are skewed towards Mac2 macrophages (Figure 3C) which is a key point here given our new chimera data shows Mac1s macrophages are replenished quicker than Mac2 macrophages, thus more reliant on recruited monocytes (Figure 3A). In addition we have also shown new data characterising the tissue resident macrophage pool in $Ccr2^{-/-}$ mice showing further changes to macrophages including enhanced expression of CD4 (Figure 3D), CD86 (Figure 3E) on Mac1s and enhanced MerTK on Mac1 and Mac2s (Figure 3F).

A representative flow plot showing loss of monocytes but a substantial population of macrophages can be seen in Extended data 7A.

4. Page 6. Gene names should be capitalized; SIRPA not Sipra.

This has now been amended in the manuscript.

5. Page 7 refers to “glandular” epithelium, but the images mainly show the luminal epithelium

This has now been amended in the manuscript.

6. Page 8: the smooth muscle 3 cells are probably pericytes since they express Rgs5.

Smooth muscle 3 cells have now been renamed pericytes, as have smooth muscle 1 since these also express RGS5 (Figure 7D and Extended data 10 and 11A). Flow cytometry gating has been updated accordingly to now include pericytes based upon expression of MCAM, CD9, CD29/Itgb1, PDPN and Thy1 on PDGFRα negative cells (Extended data 11B).

7. Page 9: “Csf1 [is] required for monocyte differentiation into macrophages” greatly oversimplifies the function of Csf1.

The context being referred to here was that alongside a multitude of functions, Csf1 is essential for monocyte differentiation into macrophages and is expressed most highly by activated fibroblasts indicating these cells may play a key role in macrophage differentiation in the uterus (as inferred from Extended data 12A).

8. The Figure 1 legend does not correspond to the panels in the figure.
This has now been amended in the manuscript.

Reviewer #2

(Remarks to the Author)
In their manuscript "The dual roles of uterine monocytes in regulation of tissue integrity throughout reproductive cycles", Shahzad et al. describe their important findings regarding monocyte and macrophage populations in the cycling uterus. They provide in depth analysis of the different populations of mononuclear phagocytes across the different cycle stages of the uterus and provide data supporting the notion that the different populations might differ substantially in their contributions to healthy tissue maintenance. The authors provide mouse data and human data side-by-side, even including those from patients suffering from Asherman's Disease, further showing the high relevance and broad applicability of their study. This is an important and extensive study that provides a much-needed basis to further understanding of an under-studied tissue with major health implications for women.

I have the following general comments on the current form of the manuscript:
1) The authors define major populations of mononuclear phagocytes by RNA-seq and follow up on the monocytes, Mac1 and Mac2 by FACS. This is very useful and in many cases the authors provide the needed depth across different cycles. This is however not always the case, leading to some ambiguity regarding the stability of the markers used. For example, CD24 was identified as declining from monocytes to Mac1 to Mac2, but a critical assessment regarding CD11c and CD206 stability (used to subset the populations) seems to be missing. The stability of these markers (against others) should be tested across the different stages of the menstrual cycle. Along those lines, can the authors provide more information on the cycle stage underlying their RNAseq data? The authors seem to have stage specific RNAseq data for their epithelial/stromal data – can this be shown individually, for example for Figure 5B. I assume this data is not available for the analysis shown in Figure 2 and understand that providing this might be challenging.

CD11c and CD206 were proposed as putative markers to identify the Mac1 and Mac2 subsets based on our single cell RNAseq data in mice (Figure 2E). We validated this at protein level and show the Mac1 and Mac2 subsets as assessed by flow cytometry in Figure 2F through different stages of the estrous cycle. We have now added flow cytometric analyses at each cycle stage of expression of multiple proteins that looked relevant from our single cell RNAseq, on monocytes, Mac1 and Mac2 macrophages, including MerTK (Extended data 5A), CX3CR1 (Extended data 5B and 4C), CD4 (Extended

data 6A), CD86 (Extended data 6B), TNFα (Extended data 6C) and CD24 (Extended data 6D).

Apologies for any confusion re cycle stages underlying the single cell RNAseq data in mice. In all cases, these data represent all stages combined and single cells were sorted from pooled uteri of 16 mice. Single cell sorting was carried out on FACS-enriched populations of myeloid cells and $CD45^-$ cells separately (FACS sorted at the same time from the pooled uteri). These were mixed samples from mice in proestrus, estrus, metestrus and diestrus, so all cycle stages together are represented in the scRNAseq data. Following the scRNAseq experiments, surface and intracellular panels for flow cytometry were designed to identify specific subsets of cells based on their patterns of gene expression. In some cases this was easy/obvious eg $CD45^-$, $CD31^-$ $EpCAM^+$ cells for epithelial cells. These flow cytometry experiments were then used to assess the contribution of the myeloid and $CD45^-$ subsets at each cycle stage. Please note that the analyses in Figure 7E and F (previously Figure 5) represents fibroblast fluctuations through the estrous cycle as assessed by flow cytometry using our gating strategy defined in Extended data 11B, rather than RNAseq analyses. We have made it clearer in the text when we are referring to RNAseq data versus follow-on flow cytometry data.

2) The authors suggest that both Mac1 and Mac2 derive from monocytes, and according to their RNAseq data, they suggest that the two populations are derived independently. Based on BM chimera data, kinetics, and Ki67 staining, the authors suggest that Mac2 are replenished slower and proliferate in situ. Given the focus of the study, it would be good to gain more insight into why and when monocytes yield different macrophage populations. Is the idea that monocytes develop into closely related pro-inflammatory Mac1 at the height of their recruitment magnitude, which coincides with tissue inflammation and that Mac2 develop from monocytes that are recruited at different cycles, which require repair? This aspect is also important to properly interpret the CCR2 KO data, for which it is unclear in the current manuscript at which cycle stage the data was from, and whether the impact of CCR2 deficiency on Mac1/Mac2 ratios differs across different stages.

We have now carried out additional experiments to gain more insight into why and when the monocytes yield different macrophage populations. First, we set up additional bone marrow chimera experiments, assessing the turnover of the specific subsets of Mac1 and Mac2 from recruited monocytes. We show that the Mac1s are replaced from recruited cells more quickly than the Mac2s (Figure 3A). Accordingly, in monocyte-deficient $Ccr2^{-/-}$ mice, there is a loss of Mac1s (that are more closely related to monocytes) and skewing towards Mac2s. In human Asherman Syndrome, monocytes and Mac1s increase while the numbers of Mac2s remain constant, consistent with Mac2s being turned over slower and thus not as much affected by increased monocyte numbers (Figure 10).

Next, we have explored molecules that may be involved in differentiation into Mac1 versus Mac2 based on their gene expression profiles. This includes TGFβ given the high expression of the receptors on the Mac2s plus the numerous TGFβ response genes expressed. TGFβ was inhibited using a blocking antibody administered i.p. every 2 days,

for 2 weeks. This is a regimen our colleagues have used to block the effects of TGFβ in the intestine (PMID: 24098124). These experiments demonstrate that TGFβ is required for monocyte differentiation into mature macrophages from the intermediary/immature Ly6C$^+$ phase (Figure 3G), and blocking TGFβ reduced the proportion of Mac2s (Figure 3H), and reduced their capacity to proliferate (Figure 3I). Another candidate molecule was IFNγ which was expressed at high level by monocytes, and by Mac1 but not Mac2 (Extended data 7L). Indeed, mice deficient in Ifngr1 had reduced levels of Mac1 and a skewing towards Mac2 (Figure 3J). Thus, we think TGFβ plays a role in Mac2 differentiation, with Mac1 differentiation at least in part governed by response to IFNγ. This fits with reviewer 2's idea that monocytes develop into Mac1s under inflammatory conditions with Mac2 differentiation occurring during less inflammatory periods.

We also provide new cycle stage information for the macrophage subsets in Ccr2$^{-/-}$ mice (Extended data 7D and 7E), demonstrating that the Mac1 and Mac2 subsets remain constant throughout the estrous cycle thus there is a constant and overall decrease of Mac1s and increase of Mac2s.

In addition to above points, following concrete comments should be addressed:
- Figure 1B: The data graphs for % vs cell numbers are extremely similar. This might be true, but can the authors double check that the data is accurate? It seems hard to believe that the total cell number so closely reflects percentage after all the necessary tissue digests etc

We agree with reviewer 2 that these values looked unusually similar. Upon re-checking our absolute number calculation spreadsheet – the total cell counts for the digested tissue had not been entered for these experiments meaning the calculations were generated from a default value that was the same for all samples (hence proportion of the same cell count in all cases leading to mirrored absolute numbers!). We apologize for this oversight and have now entered the correct cell counts in this column, and recalculated the absolute numbers (Figure 1B).

- Fig. 1D, E: In the text, the authors write that the replenishment of uterine macs closely mimics that of the colon, which really is not true for the 8 week timepoint.

The text has now been amended to say that there is initially a slower replenishment than the colon up to 8 weeks after irradiation but by 12 weeks the proportions of tissue macrophages replenished from the bloodstream matches that of the colon.

In addition: Why is the WT control between 1D and 1E so different? And: The legends do not fit the figure

Experiments in 1D were carried out in Manchester, experiments in 1E were carried out by collaborators at the Pasteur Institut. We have realised that the values calculated for chimerism in the experiments from the Pasteur (Ccr2$^{-/-}$ Figure 1E) were not normalised (to chimerism in the blood), whereas our Manchester data (Figure 1D) normalised the chimerism of uterine macrophages to blood monocytes. This is required since the

irradiation does not uniformly deplete all immune cells in bone marrow, in particular since these are shielded chimeras. However normalisation to blood monocytes cannot be done in the $Ccr2^{-/-}$ experiments since $Ccr2^{-/-}$ mice do not have circulating monocytes. Therefore we have amended Figure 1E, so that in all experiments (wildtype and $Ccr2^{-/-}$), the chimerism of uterine macrophages has been normalised to blood neutrophils. The wildtype values in terms of replenishment of uterine macrophages now reflect very closely what we see in the Manchester experiments in Figure 1D, and there is very little turnover of uterine macrophages from the bloodstream when bone marrow from $Ccr2^{-/-}$ mice is used to replenish irradiated mice. We have also included additional data showing chimerism in mice replenished with wildtype versus $Ccr2^{-/-}$ bone marrow with a 24 week period before analyses.

The legends have been amended.

- Fig. 2G: FMOs or isotype controls are missing. Maybe MFI would be better for all markers as there is no bi-model distribution, and the cut-off seems arbitrary?

In all flow cytometry experiments, staining positivity was derived using gating strategies based on FMOs. Due to the addition of several new graphs into figure 2 we now show summary data for comparison of markers MerTK, CX3CR1, CD4, CD86, TNFα and CD4 between monocytes, Mac1s and Mac2s (Figure 2G-L) with further information on expression of these markers at every cycle stage in Extended data 5 and 6. Thus for space purposes we have taken out representative FACS plots but we can add in at reviewers request if necessary. We have not used MFI in most cases (except CX3CR1 since expression is almost 100% in all cases for proportion of cells, it was not possible to compare across cell types or cycle stages) because we find batch effects for MFI of running samples on different days especially after the flow cytometer has been serviced.

- Fig.3B: Monocytes are Mac1s do not seem different across the PCA. Can the authors comment on that?

We think gene expression in Mac1s is very closely related to monocytes because they are recently differentiated, ie more recently replenished from blood monocytes compared to the Mac2s. This is reflective of what happens at other mucosal sites e.g. the lung after flu infection which causes recruitment of monocytes and subsequent differentiation into alveolar macrophages. These recently differentiated macrophages retain monocyte reactivity and epigenetic makeup (referred to as "monocyte legacy"; PMID 31932810). However macrophages that have been recruited but have resided in the lung over time have a different signature and appear more like tissue resident populations (termed "immunosedation"), which we think may be reflective of our Mac2s that we show are turning over more slowly in mice, and in humans express FOLR2 which has been associated with tissue residency in single cell studies of the endometrium that have characterised macrophage subsets. This has all been included in the results and discussion.

Figure 4D shows that monocytes do have a different gene signature to Mac1s. As expected, the latter show increased expression of genes associated with macrophage differentiation and maturity such as IFNLR1, ADAMDEC1 and MMP12.

- Fig.5E-H and accompanying results: The authors make strong claims regarding the interactions between monocytes and fibroblasts, but in the currently depicted form, this seems somewhat biased, as they are only showing the interactions from monocytes and no comparisons to the other subsets. The text describes this somewhat, but it might be better to provide the data in the figure. In its current form, I find the notion surrounding monocyte and their role in collagen regulation somewhat overstated, especially since CCR2 deficiency should affect all subsets according to their shared monocyte dependency as suggested by the authors.

These data are now in Figure 7 and in addition to the predicted interactions between monocytes and ECM-producing CD45$^-$ cells (Figure 7I, J and K), we have now added further predicted interactions between Mac1/CD45$^-$ cells and Mac2/CD45$^-$ cells (related to ECM) in Extended data 12 and 13. These interactions are similar especially for Mac2s, but monocytes came out as having the strongest interactions (Figure 7H) because more of them express molecules that bind ECM-producing cells and thus their density of expression of these molecules greater. To make this clearer, we have now added extra violin plots to Extended data 14, showing the relative expression of these specific molecules across the monocyte and macrophage subsets. It is clear, for example, that Itga1 and Itga5 are only expressed substantially on monocytes. However as there are clearly reasonable interactions for the Mac2s also, we have amended the text and the message here so that it does not come across that only the monocytes may be capable of interacting with fibroblasts. We feel that monocytes interacting with fibroblasts and having a role in ECM modulation (even if alongside Mac2s) is notable as the current dogma has this as primarily a role for alternatively activated (CD206$^+$) macrophages, and so this role for monocytes is unusual.

We have shown that CCR2 deficiency results in no monocytes in the uterus but these mice do have substantial numbers of macrophages (albeit reduced and skewed towards Mac2s; Extended data 7A-D)). Thus there must be some redundancy mechanisms present (such as self renewal) to maintain tissue macrophages in the absence of monocyte recruitment. Although CCR2 deficiency does affect all subsets to some degree, the monocytes are much more profoundly affected. Given the excess collagen deposition in the Ccr2$^{-/-}$ mice in the complete absence of monocytes but presence of other macrophage subsets, we suggest that monocytes play a key role in regulation of collagen.

- Related to Figure 6: It is not very clear how the different stages of the cycle were defined in the mice, as the authors only refer to a previous publication. Is the method suited for young and old mice alike? Are there any differences?

We have used the same method for staging young and old mice (microscopic assessment of vaginal lavage and comparison of ratios of leucocytes to cornified and non-cornified epithelial cells). This was clear in both young and older mice – as our older mice were around 9 months of age they were still having regular estrous cycles (for this reason we used older mice rather than aged mice (16-24 months)). We have included here examples from our own experiments (attached for reviewers but not included in the figures – we can add these in if reviewers deem it necessary).

- Introduction: The general immunology audience might need a slightly more extensive explanation of the different cycles, when what happens and what is known about macrophages in the uterus to date. As is, the introduction is very brief and does not provide the necessary foundation in my opinion

The introduction has now been amended.

- Histology: Some panels are small, and the stain is hard to see – maybe zooming into some picture would be useful? Some size bars are not readable. For quantifications of absolute numbers, an explanation is missing (cells with field of view?)

We have now included zoomed in versions of the samples in Extended data 8E and G to make the stain clearer to see (assuming this is referring to the cleaved caspase 3 staining in Figure 6C and D). Size bars have now been amended to a readable size and explanation regarding absolute numbers from the images has now been included in the methods.

- Data: Information on how many times individual experiments were performed is missing, so there is a potential need for repeats for some data panels.

Every experiment in the manuscript was carried out with at least one repeat, and we have amended the figure legends to clarify that all experiments were performed independently at least twice. For most of the Ccr2$^{-/-}$ data characterising CD45^{-} cells, there are fewer data points than we would ideally like because we are breeding heterozygotes so needed to wait for the progeny with full KO phenotype and on top of that only use females, then collect information in every cycle stage. As these experiments were carried out as and when mice were available (alongside littermate controls), there are multiple repeats despite the relatively low n numbers in some cases.

- The manuscript should be carefully checked for typos and the correct links to data panels

We apologies for this oversight. The manuscript has been checked rigorously for typos and linking text to data panels.

Reviewer #3

(Remarks to the Author)
The author's have undertaken a detailed study of the role of infiltrating monocytes in mouse and human endometrium across the estrus and menstrual cycle, respectively, using flow cytometric analysis involving multiple surface markers and clearly described gating strategies, scRNAseq of mouse uteri, transgenic mice (Ccr2-/- mice), hyperion mass cytometry of human endometrium and computational approaches. They demonstrated that monocyte proportions varied dynamically over the reproductive cycle in mice and women and that they contributed to endometrial remodelling by inducing epithelial apoptosis. Endometrial monocytes and their macrophage derivatives also influence extracellular matrix remodelling, which they suggest differs between monocytes and Mac2s. Finally, they assess the relative roles of monocytes and Macs in ECM in endometrial fibrosis in aging mice and while they demonstrate alterations in cell types associated with fibrosis and the monocyte and macrophage populations in Ccr2-/- mice, the amount of collagen is not different to WT and the "disorganised collagen" appearance is not convincing given the overproduction of endometrial glands in this model.

As discussed above re reviewer 1, the $Ccr2^{-/-}$ data in older mice re collagen content has been removed, as the rest of the data in this figure are important to make the point of the involvement of inflammatory monocytes and Mac1s in fibrosis, and the loss of dynamics through the cycle in general within the myeloid and $CD45^{-}$ lineages. This is important due to the similarity with human uterine fibrosis/Asherman Syndrome; we have now included additional data showing that regardless of cycle stage, there are sustained high numbers of monocytes in uterine fibrosis (Figure 10G).

Asherman's syndrome characterised by replacement of endometrium with fibrotic tissue was shown to contain fibrotic tissue (excess collagen), surgery did not improve this, not surprising as fibrosis often recurs in this condition.
Strengths of the manuscript include clarity of the data presented in figures, use of pre-enriched myeloid cells for the scRNAseq study of mouse endometrium, the use of state-of-the-art technologies to identify the mechanisms involved as they undertook a detailed investigation of monocyte function in human and mouse endometrium. They provide new data on their role in endometrial function over the menstrual cycle (albeit needs some revision) and in fibrotic conditions of a generally neglected tissue which has major health implications for very large numbers of women globally.

Weaknesses include
1. There is a fundamental issue in the manuscript in equating endometrial changes in mouse proestrus and human early secretory stages of their reproductive cycles which is not correct and needs addressing. Ovulation occurs in humans in the interval phase at the end of the proliferative phase (circulating estrogen levels rise and dominate during this stage) and not in the early secretory stage, which occurs after ovulation, when progesterone levels begin to rise and endometrial cell proliferation rates substantially reduce. The early secretory stage is observed histologically by epithelial nuclear movement towards the apical lumen appearing as subnuclear vacuoles, due to progesterone-induced secretion of glycogen. In mice, ovulation occurs in the estrus

phase and proestrus is most similar to the human proliferative phase where estrogen levels are rising. Metestrus is most equivalent to the human early secretary and mid secretory phase characterised by both estrogen and progesterone. Progesterone is still present in the diestrus and most like the human late secretory phase (Wood GA et al Reprod (2007; Ajayi & Akhigbe PMID: 32190339). This means the peak monocyte infiltration in mice and humans are at functionally different cycle stages (Fig 1), prior to ovulation in mice and following ovulation in humans. The graphs presented in Fig 1A should be replotted to account for these differences (proestrus as the first bar) and to assist data interpretation, clearly indicating the histological criteria used for staging the human samples as the "next predicted menstrual period" can be inaccurate in normal women with variable cycle lengths, and more so for Asherman's syndrome patients. Revision of the text in the Results and Discussion will also be required.

We have amended the text in the manuscript so that we are not equating proestrus in mice with the early secretory phase in humans, and have simply said that monocyte infiltration occurs in the cycle stage preceding the window of implantation in both species (monocyte infiltrates in proestrus and early secretory phase). This is important as following infiltration, bona fide monocytes will begin to turn into inflammatory "intermediate" type cells as they are differentiating, present in the next cycle stage (window of implantation). This can be seen clearly in estrus: Ly6C^{hi} monocytes begin to express MHC class II as they differentiate into macrophages, passing through an "intermediary" phase expressing Ly6C and MHC class II (Extended data 1A). This phenomenon has been evidenced widely in the intestine and it is this potently inflammatory intermediary population that accumulates in the intestine of mouse in IBD models. In support of this, our mouse scRNAseq data clearly show an "intermediary" population in between the monocytes and the Mac1s (Figure 2).

For analysis purposes we have classified all Ly6C^{+} cells as monocytes but this does include this intermediary population that also expresses MHC class II. It is, however, difficult to mirror these analyses in humans in defining intermediary monocyte/macrophages and which cycle stage they accumulate in, since there is no Ly6C or F4/80 equivalent and human monocytes as well as macrophages express HLA-DR so it is difficult to visualise differentiation trajectories. Thus, we are distinguishing human monocytes from macrophages (following additional gating strategies) based on one marker (CCR2) rather than two.

We request that order of cycle stages on graphs may be amended following this round of review (if positive outcome) as changing all graphs describing mouse data through estrous cycle stages to begin with proestrus, currently applies to Figures 1,2,7,8 and Extended data 1,4,5,6,7,10,14.

We have only collected endometrial biopsies from women with regular cycles for the reasons mentioned by reviewer 3 regarding staging of human samples, which has been explained in the methods. As explained in more detail below, we have specifically used our Asherman Syndrome cohort patient that are still having regular menstrual cycles (albeit very light periods), and thus can be stratified into cycle stages. This is important

because the post-surgery patients are all on estrogen to induce endometrial proliferation, thus mimicking the proliferative phase of the cycle. Therefore we need our pre-surgery cohort to also be in the proliferative phase for comparisons, as well as our healthy controls. We have now included additional data showing that regardless of cycle stage in Asherman Syndrome patients, there are sustained high numbers of monocytes in the endometrium (Figure 10G).

2. A Methodological issue with the immunofluorescent images from the Hyperion imaging system as there are some immunostaining is not representative using traditional IF techniques and of known endometrial biology. More detail is needed on how the Panels were established and verified for accuracy. Some high power images would assist showing where immune cells are assessed and maybe showing controls used when establishing the panels in Ext data figures.

Our Hyperion panels were designed with our Core Facility team at the University of Manchester who have worked with the immunology department within the Lydia Becker Institute and the Manchester Matrix Centre to generate a multitude of pre-designed panels for matrix and immune analyses using optimised antibodies and metal conjugation combinations in human tissue. To verify our specific panel for accuracy, we have now included immunofluorescence staining with traditional techniques using the very same antibodies for our key markers, including matched isotype controls for each, and with additional controls for secondary staining (no primary). This has now been added into Extended data 9C, 17 and 18.

3. Statistical analysis was well described and appropriate and while detailed in the Figure legends, there was no indication of what the bars or data points were. All appeared to be plotted as means and ?SD or ?SEM, but for non-parametric analyses, medians with interquartile range should be plotted as this is what is statistically different. This will alter how the authors describe their data.

In all cases, normality testing has been carried out. Data are plotted as mean+/-SEM for parametric data or median+/- interquartile range for non-parametric data. This has been described in the statistics section of the methods and the figure legends describe which statistical tests apply to each graph.

Specific Issues

4. While the authors clearly show their gating strategy for distinguishing monocytes from macrophages, it is not clear that they actually achieve this (Ext Fig 1) as some mouse monocytes are Ly6C+ and/or MHC-II+ and some human monocytes are CCR2+. As the authors show, these expressions can change in different stages of the menstrual cycle, which they investigate further throughout the manuscript. Why were additional markers not considered eg F4/80 (mouse), CD16 (human)? However, later in the manuscript, they have used additional markers based on their findings from the single cell data.

In mice, after pre-gating to exclude granulocytes and selecting for $CD64^+$ and $CD11b^+$ cells, all $Ly6C^+$ cells should be monocytes given this marks monocytes in all other tissues, and we have now included additional data in Extended data 1B showing that $Ly6C^+$ monocytes express low levels of F4/80 compared with macrophages. F4/80 expression is particularly low on monocytes during proestrus when they infiltrate the uterus but is upregulated during estrus as they begin to turn into macrophages (Extended data 1C), but remains consistently high on the differentiated macrophages. $Ly6C^+$ monocytes begin to express MHC class II as they are turning into macrophages along a differentiation continuum, thus accordingly, levels of MHC class II positively correlate with F4/80 (Extended data 1E). This has been discussed in more detail in the manuscript, in particular the accumulation of the intermediary cells during estrus. In addition, other markers have now been analysed on monocytes and macrophages based on our single cell RNAseq data, including MerTK, CX_3CR1, CD4, CD86, CD24 and TNFα (Extended data 5 and 6). CD24 in particular appears to be an excellent marker of murine uterine monocytes and is expressed more on Mac1 macrophages rather than Mac2 macrophages in keeping with Mac1 macrophages being more recently replenished from monocytes.

In humans, we have used CCR2 as our monocyte marker, given there is no Ly6C equivalent and we know that replenishment of uterine macrophages in mice is dependent upon recruitment of $CCR2^+$ monocytes. This population will likely include monocytes that are in the process of turning into macrophages. We have now shown addition data demonstrating that the $CCR2^+$ cells are more monocyte-like in size/granularity than the $CCR2^-$ population that look more representative of differentiated macrophages (bigger and more granular; Extended data 2B). In addition, we have further sub-characterised our human $CCR2^+$ monocytes, based upon levels of expression of HLA-DR (high or low) and characterised expression of a range of molecules on these monocyte subsets alongside Mac1 and Mac2 (Figure 5), including CD16 (Figure 5H). Although CD16 is expressed on a subset of circulating blood monocytes (non-classical and intermediary), it is also upregulated on macrophages upon maturation in some cases and it would appear this may be happening here. The $HLA\text{-}DR^{lo}$ subset of monocytes that fluctuate through the cycle likely represent recently recruited monocytes. They have virtually no CD16, indicating they may resemble classical blood monocytes. CD16 is slightly higher on the $HLA\text{-}DR^{hi}$ subset of monocytes and Mac1s, but is significantly higher on the Mac2s which we believe to have been in the tissue longest (Figure 5). Thus, whilst this is of interest and has been discussed in the manuscript, we do not believe CD16 is a useful marker to distinguish monocytes and macrophages in the human uterus.

5. Fig 1 – the figure legends do not match the panels correctly and as described in the text.
This has now been amended.

p3, 2nd para –peak monocyte influx in mice is in the pre-ovulation stage proestrus phase (estrogen dominant, equivalent to late proliferative in human), but in humans this occurs post ovulation in the early secretory stage.

As discussed above, text regarding monocyte infiltration and cycle stage in mice versus humans has now all been amended.

6. Extended data Fig 3D does not have a figure legend.
This has now been amended.

7. P5 middle of bottom para – should be Fig 1a rather than 1c. 7 lines from bottom, Fig1a rather than 1b. Should the last line be Fig 2g?
Yes, these errors have now been corrected.

8. P6 – top para – which stage of the estrus cycle was the experiment shown in ext data Fig 4a undertaken, as Lys6C varies in monocytes and macrophages across the estrus cycle.
TNFα production by monocytes and macrophage subsets in mice has now been assessed at all cycle stages and these new data are described in Extended data 6C. Monocytes are potent TNFα producers at all cycle stages, producing more than either macrophage subset in both mice (Figure 2K and Extended data 6C) and humans (Extended data 8C) .

9. P 6 2nd para – reword the sentence commencing "In support of less recent turnover of Mac2" to clarify its meaning. Delete '"monocytes and" in sentence describing Fig 3f – only Mac1 and Mac2 are shown in this panel.
This has now been amended in the text.

10. P6 2nd to last line – refer to Fig 3e
This has now been amended.

11. Page 7, 1st para – there is a week before the window of implantation occurs in humans (ie mid secretory stage), so the monocytes must differentiate as indicated by Fig 3g, perhaps into Mac2?

Of note, absolute numbers of human macrophage subsets have now been added to this figure (now Figure 4) showing that the numbers of Mac2s stay constant through the cycle (although there is an overall trend of more in general during the late secretory phase), but the Mac1s increase during the early secretory phase. This is in keeping with Mac1s being differentiated from monocytes before Mac2s. There is some cleaved caspase 3 staining in HLA-DR cells throughout the cycle and in particular during the proliferative phase (Figure 1C), which could represent apoptotic monocytes/macrophages. It is possible that some may carry out their inflammatory and/or ECM modulating functions then apoptose, but developing this idea is outwith the scope of this paper. It is difficult in humans to characterise the "intermediates" that are still monocytes but turning into macrophages, due to the lack of specific monocyte markers.

12. P 7 2nd para – line 2 – should be Fig 2d – please check all the text throughout the manuscript for accuracy of the referring to Fig panels.

The manuscript has now been thoroughly checked for accuracy of panels and all the new Figures.

What are the "immature progeny of monocytes"?

This phrase has now been changed in the manuscript but refers to immature macrophages that have recently or are still differentiating from monocytes. This has been clarified especially in light of the discussions above re $Ly6C^{+}$MHC class II^{+} intermediary cells.

13. Fig 4 – Panel D - please quantify the CIC3+ epithelial cells for the estrus cycle stages as done for Ccr2-/- mice.

$ClC3^{+}$ epithelial cells have now been quantified at all cycle stages and this has been added to Extended data 8F.

Panel E needs scale bars (magnification looks different between the 2 images) and a more representative image of wt estrus endometrium which should show some gland profiles to match the data shown for estrus in Panel F. Panel G, why are there 2 types of dot points in the graph?

Scale bars have been added to this panel which is now 6E. Apologies we are unsure what reviewer 3 means here in terms of a more representative image of a WT estrus endometrium, or for the two types of dot points in the flow cytometry data (now Figure 6F).

Panel H –Granzyme B is difficult to see visually – please include the control Ab Panels used to generate the set of antibodies for this Fig. In the Fig Legend, indicate the stage of the menstrual cycle forthis section. Maybe also include a late proliferative stage endometrium which is more comparable to mouse proestrus stage. And more examples of HLA-DR+ macrophages clustered in close proximity of human endometrial gland profiles could also be shown in an Ext Data figure.

The granzyme B antibody we have used has been verified using traditional immunofluorescence techniques and this is included in Extended data 9C with the matched isotype control. We have now used a clearer Hyperion image for HLA-$DR^{+}ClC3^{+}$ cells clustering next to the human early secretory phase glandular epithelium (Figure 6H). Healthy control proliferative phase samples do not exhibit clustering of monocyte/Mac1s with the epithelium. We show this in Extended data 16C with 3 additional examples of monocyte/Mac1s next to epithelium in Extended data 16D during the early secretory phase.

We have now included statistical analyses from our Hyperion data determining spatial characteristics e.g. average distance of monocytes/Mac1s from epithelial cells and interactions between monocyte/Mac1s and epithelial cells(Figure 9H-J). Flow cytometry at greater cellular resolution revealed a massive increase in particular in HLA-DR^{lo} monocytes and Mac1 macrophages in Asherman Syndrome patients (Figure 10), with these inflammatory monocytes and macrophages obviously clustering next to the epithelium in Figure 9 (H-J). The spatial proximity of monocytes/macrophages to

epithelium were not possible to compare across the cycle in healthy individuals because although there was more clustering of monocytes/Mac1s next to the epithelium in the early secretory phase, there were generally many more glands in the late secretory phase; although there no was no obvious clustering, the proximity of monocytes/Mac1s to epithelium was always close due to overall increased density of the glands (this can be seen in the late secretory phase in Extended data 15C).

14. Fig 5 Panel A Only pro-estrus is shown. To demonstrate that more collagen is deposited at this stage, all stages of the estrus cycle should be shown in Ccr2-/- mice.
All stages of the estrus cycle have now been shown re WT and $Ccr2^{-/-}$ comparison for collagen deposition (proestrus: Figure 7A and B; other stages Extended data 9D).

p 8, clarify what a "network" of various endometrial cells is in the scRNA seq data?
This simply referred to the multiple different cell types identified from the scRNAseq data. Different language has now been used in the manuscript.

Fig 5c –Why are there different number of replicates for some of the cycle stages as the same flow plots are used for determining activated fibroblasts and myofibroblasts?
This has been amended - there were two experiments in which we did not have a Thy1/CD90 antibody. So two of the activated fibroblast datapoints actually represented all fibroblasts and thus have now been removed.

Medians should be plotted for the myofibroblast graph for panel c and d. Has the analysis conducted for Panels f-h been assessed for interactions with Mac2 as the CellChat suggests they are similar (Panel e)? This would strengthen the interpretation that incoming monocytes are more likely to interact with fibroblasts than Mac2s.
Medians have now been plotted for myofibroblasts in Figure 7F,G.

As discussed with reviewers above re CellChat analysis: These data are now in Figure 7 (monocytes and ECM-producing $CD45^-$ cells) and we have now added further predicted interactions between Mac1/$CD45^-$ cells and Mac2/$CD45^-$ cells (related to ECM) in Extended data 12 and 13. These interactions are somewhat similar especially for Mac2s, but the reason the monocytes came out as having the strongest interactions with ECM-producing cells (Figure 7H) is because they express a greater density of of the respective binding components. To make this clearer, we have now added extra violin plots to Extended data 14, showing the relative expression of these molecules in the monocyte and macrophage subsets. It is clear, for example, that Itga1 and Itga5 are only expressed substantially on monocytes, though the data predict that macrophages, especially Mac2s, will also actively interact with fibroblast ECM, We have amended the text to convey this message as the current dogma is that alternatively activated macrophages modulate ECM and contribute to tissue repair, and so potentially stronger interactions amongst monocytes is notable.

Similarly for ext Fig 7, what do myofibroblasts interactions with monocytes and Mac2 show? Also monocytes and Mac2 seem to show very similar interaction probabilities in this fig.

Myrofibroblast interactions with monocytes, Mac1 and Mac2s are now shown in Extended data 12B, which brought up only one ligand-receptor pair (CXCL12-CXCR4). As described above, for activated fibroblasts and myeloid cells, the interactions themselves are similar for monocytes and Mac2s but it is unusual for monocytes to interact with ECM at a level comparable with that of CD206^{+} macrophages. In addition, there is a greater density of monocytes expressing the appropriate binding partners than Mac2s (Extended data 13). For example it is clear that only monocytes express Itga1 and Itga5.

15. Fig 6 –were equal numbers of mice used from each cycle stage comparison between young and older mice to produce the graphs in all the various panels as the staining in the images show quite different staining intensities at different stages and was more easily observed in the images showing better examples of longitudinal sections. This needs to be included in the Figure legend. Where non-parametric statistical tests were done, medians and IQRs should be plotted. The tissue block used for the aged estrus uterus is not a good example of a longitudinal section in both Fig 6 and ext Fig 8.

We have now re-cut and stained longitudinal sections for Figure 8 (previously Figure 6) and Extended data 15 (previously Extended data 8), for all cycle stages in young and old based on this comment and from other reviewers. For the young versus older mice re collagen content, there were equal proportions of mice from each cycle stage for each group. In addition, we have carried out a hydroxyproline assay on selected samples to definitively show excess collagen deposition in the older mice (Figure 8B). Due to the expense of the kit (we were required to use this for Ccr2$^{-/-}$ experiments also from reviewer 1), we confined the analysis to mice in proestrus (this has also been clarified in the figure legend). Data from both collagen-rich areas plotted from PSR imaging, and the hydroxyproline assay, passed normality testing (Shapiro-Wilk) and thus mean +/- SEM are shown, with comparison by student's t-test (described in figure legends).

The older CCR2-/- mice uteri look like they still have many glands which more likely contributes to the disorganised look of Sirius red staining rather than a real effect .

The PSR staining in the older Ccr2$^{-/-}$ mice has now been removed since we did not see a difference in collagen and there was a lack of clarity re disorganised structure.

(As discussed above with reviewer 1): We think that since the collagen deposition and level of fibrosis is already extensive in the older mice (wildtype), the phenotype in the Ccr2$^{-/-}$ mice may be too subtle to see a difference in total amount of collagen. We have now removed the Ccr2$^{-/-}$ data to avoid any confusion. The main point of this figure was to assess the contribution of CD45^{-} cells, monocytes and macrophages to the fibrotic environment in mice and we think it is still important to point out the loss of fluctuations in epithelial cells, fibroblasts, monocytes and macrophages in the fibrotic uterine environment in mice. This may seem unusual when compared to fibrosis in other tissues, where commonly higher numbers of fibroblasts are seen alongside dysregulated

epithelial cells, but this makes sense in the uterus due to the drastically changing tissue environment, meaning the fluctuations themselves are critical for maintaining a "normal" structure.

Importantly these mouse data mirror our findings in human uterine fibrosis (Asherman Syndrome) re sustained loss of epithelia and high numbers of monocytes and inflammatory macrophages with a general loss of tissue dynamics (reduced fluctuations in epithelial and endometrial structure so the endometrium remains uniformly thin).

For ease of comparison in Figure 9 and 10 (Asherman Syndrome), we have used data from patients with regular menstrual cycles (albeit light periods and still an abnormally thin endometrium) and thus can be stratified into cycle stages. This is important so that we can compare them to healthy controls in specific cycle stages. Furthermore the post-surgery patients are all on estrogen treatment which mimics the proliferative phase to induce endometrial proliferation/thickening. Thus we need the pre-surgery patients to also be in the proliferative phase of the cycle to be able to compare fibrosis patients pre- and post-surgery. Accordingly we then needed our healthy controls to also be in the proliferative phase. We have now added data including information from Asherman Syndrome patients in other cycle stages to show that regardless of cycle stage, there are always sustained high numbers of monocytes and thus the normal/homeostatic kinetics and rhythmicity seen in healthy controls is lost (Figure 10G).

16. P9 last para – thinner endometrium with fewer gland would be a more accurate term than "thinner glandular layer."
This has now been amended.

17. Suppl Table 1 gives a good summary of patient demographics, including for Asherman's syndrome patients. However, the severity of the disorder and a menstruation history is very important, but not included. How many Asherman's patients were menstruating and how regular were their cycles and how heavy were their periods. Severe Asherman's women do not menstruate and those that do often have scanty menstrual flow. Is it possible to get this information from the pathology reports? Please note that a total of n=24 samples were obtained in the Control proliferative stage but the numbers add to n=25 for ethnicity.
The methods (re sampling) and text in the discussion have now been amended to clarify that we have deliberately selected Asherman Syndrome patients regular cycles (i.e. all were menstruating, albeit all with light/very light periods). This was important because we are comparing patients with endometrial fibrosis to those post-surgery when patients are all on estrogen to induce endometrial proliferation (thus reflecting proliferative phase). Therefore, for these comparisons, we selected pre-surgery patients who were in the proliferative phase only, to compare to healthy controls in the proliferative phase. We have however now added in additional analyses by flow cytometry in Figure 10, to show that regardless of cycle stage, monocyte numbers remain high in the endometrium of Asherman Syndrome patients (Figure 10G; now including secretory phase (2 from early, 2 from mid, 2 from late secretory and the same ratios for healthy controls). The demographics table has been updated accordingly. As before, all Asherman Syndrome patients have regular cycles and light periods.

N numbers for the proliferative controls have been amended.

18. Fig 7 Panel A – How were the Control panels established for these images as there is intraluminal collagen staining artifact in the Mid-secretory image and the number of proliferating stromal cells is high in the mid and late secretory endometrium and does not match the graphed data. Are medians plotted in the figure? Ext Fig 9b shows graphs from the epithelial and stromal cell flow plots but they are not representative of human endometrial histology. The number of glands (epithelial cells) increases across the cycle and generally comprises up to >30% and the relative proportion of stromal cells decreases. It would appear that the dissociation protocol has not worked sufficiently and particularly in the mid and late secretory stages when the epithelial cells are post-mitotic and difficult to dissociate without using very strong enzyme solutions.

As explained above: Our Hyperion panels were designed with our Core Facility team at the University of Manchester who have worked with the immunology department within the Lydia Becker Institute and the Manchester Matrix Centre to generate a multitude of pre-designed panels for matrix and immune analyses using optimised antibodies and metal conjugation combinations in human tissue. To verify our specific panel for accuracy, we have now included immunofluorescence staining with traditional techniques using the very same antibodies for our key markers, including matched isotype controls for each, and with additional controls for secondary staining (no primary). This has now been added into Extended data 9C, 17 and 18.

We agree there was an intraluminal collagen artifact in the previous version of the submitted manuscript. We have chosen different representative images for PanCK, Collagen and Ki67 across the cycle which can now been seen in Figure 9A. Paired with these representative images, additional statistical analyses has been carried out in conjunction with a Hyperion expert working within the Core Facility, demonstrating Ki67 in stromal and epithelial cells across the cycle (Figure 9b) and the overall proportions of epithelial and stromal cells through the cycle (Figure 9c). Medians and interquartile ranges are plotted in all cases as none of these data passed normality testing. We have now removed flow cytometry analysis for stromal cells as we agree the dissociation protocol that is optimised for immune cells does not fully digest the $CD45^-$ compartment which would have accounted for the anomalous previous flow cytometry results. Instead the entirity of Figure 9 refers to Hyperion data and analyses, aiming to be both clearer and much more representative of what is occurring during the menstrual cycle.

We have an additional figure (10) with extra data regarding flow cytometric breakdown of immune cell subsets (including new analyses splitting monocytes into $HlA\text{-}DR^{hi}$ and $HLA\text{-}DR^{lo}$ based upon our assessment of the cells in Figure 5), and additional information regarding cycle stages in Asherman Syndrome. We have separated these flow data into a different figure to avoid confusion re which graphs refer to Hyperion or Flow Cytometry. We have still included here the epithelial cell characterisation by flow cytometry since this mirrors the Hyperion data exactly with a loss of epithelium in fibrosis with restoration post-surgery. The point we make with these two figures is that epithelial proliferation is

dysfunctional and epithelial numbers low in Asherman Syndrome, which we have linked to the increased infiltration of inflammatory monocytes/Mac1s and their peri-glandular clustering. We aim to include data supporting the notion that while some aspects of the histoarchitecture of the tissue are restored i.e. epithelial cell numbers and clearance of collagen, the immune dysfunction very much persists.

P10 top para – the sentence suggesting that stromal cells increase across the healthy menstrual cycle is incorrect – most are observed in the proliferative stage and least in the secretory stages and vice-versa for the epithelial cells and should be reflected in the flow cytometry as glands as epithelial cells proliferate at a much higher rate than stromal cells. Very few Ki-67+ cells are seen in glands and stroma in the late secretory stage.

We agree and the data were wrong for the reasons mentioned above and have been replaced with Hyperion data showing highest proportions of stromal cells during the proliferative phase that decrease through the cycle, with the opposite patterns for epithelial cells. Statistical analyses of Ki67 expression in epithelial and stromal cells across the cycle has been carried out from the Hyperion data and, as shown in the figures, glandular epithelial cells proliferate the most in the proliferative phase then this decreases through the cycle, and the same pattern occurs in stromal cells. The text has been amended accordingly.

19. Fig 7- Panel C shows too many Ki-67+ "stromal nuclei" suggesting artifact in the stroma of the post surgery group. More details are required how the panel was established for this set of images.
The post surgery patients are all taking estrogen to induce endometrial proliferation, so it would be expected that there would be Ki67 staining within the stroma in this group. Control staining using the same antibodies for traditional immunofluorescence, alongside isotype matched controls and additional controls showing secondary staining but no primary staining can see seen in Extended data 17 and 18 (specifically Extended data 16b for Ki67). Panels were established as described above – in conjunction with the Core Facility using previously optimised immune and matrix antibodies for human tissue.

There are 2 panel Ds, please correct.
This has now been corrected.

Panel E - the post surgery pro-inflammatory Mac1s and monocytes also appear clustered as in the fibrosis section and is reflected in Panel F where there is no difference between fibrosis and post surgery for these cells, similarly for Mac2s.
The key points we are making here are that a) the $HLA\text{-}DR^+$ pool in Asherman Syndrome is dominated by inflammatory monocytes/Mac1s and b) $HLA\text{-}DR^+$ cells (most of which are monocytes/Mac1s) cluster next to the epithelium. Since it is difficult to resolve the specific monocyte and macrophage subsets by Hyperion, we used flow cytometry (Figure 10) to subset in depth, revealing two distinct subsets of monocytes and the Mac1

and Mac2 macrophages. It is evident from Figure 10/flow cytometry that Mac2s stay constant but there are massively increased numbers of monocytes and Mac1s in Asherman Syndrome, making the clustering of monos/Mac1s next to the glandular epithelium in Figure 9 more relevant than for the Mac2s.

However all 3 cell types are significantly different in the diseased and surgically treated tissues compared to the controls.
As mentioned above, we have now included additional analyses re absolute numbers for flow cytometry that demonstrate the Mac2s remain constant in number but the monocytes and Mac1s are increased in Asherman Syndrome (Figure 10B-F).

This needs to be made clearer in the text P10 last para.
The text has now been amended to be clearer.

The broad spectrum of patient presentation in intra uterine adhesions/Ashermans syndrome makes it difficult to see differences, particularly if the patient menstrual data is not known.
All Asherman Syndrome patients had regular menstrual cycles, although all had light periods. This was so that we could assess specific cycle stages which is important given the comparisons with the post-surgery group as described above (on estrogen to induce endometrial proliferation thus need proliferative phase comparisons). For all the Hyperion data (Figure 9) and most of the flow cytometry data (Figure 10) patients and controls are in proliferative phase for this reason. We have now added further analyses re monocytes and Mac1s in the endometrium of Asherman patients in the secretory phase of the cycle, compared to controls in the same stages (in both cases equal ratios of early:mid:late secretory; Figure 10G and H). The text here has been made clearer.

For Hyperion we have now included additional spatial analyses, demonstrating that the mean distance between monocytes/Mac1s and epithelial cells is reduced in Asherman Syndrome consistent with the prediction of more interactions between the two populations (Figure 9I and J). Visually, the elevated numbers of inflammatory monocytes/macrophages are evident, alongside clear evidence of clustering next to epithelial cells (Figure 9H).

Discussion
20. The authors point to the lack of studies demonstrating a role for monocytes in endometrial function and that their roles have not been identified in recent high profile single cell studies in mouse or human reproductive tract. This is true in part but also an over a misinterpretation of the literature. The problem is the overlapping markers for monocytes and macrophages make this difficult to study as the authors have mentiond. The authors' study does provide a very detailed study of the role of monocytes in endometrium. Novel is their use of pre-enriching the myeloid cell

populations in their scRNAseq study. However, they did not compare their findings with that in the Bunis et al paper (ref 37) which did include a set of control women and did report the monocytes as a separate population, demonstrating they increase in the mid secretory stage, compared to earlier cycle stages.

We have now discussed the Bunis et al paper, alongside another single cell paper that has identified two subsets of monocytes in the uterus (expressing CD14 or CD16; PMID 38566989). The latter has been discussed in the context of our own analyses of two subsets of HLA-DR^{hi} and HLA-DR^{lo} monocytes. We have also assessed CD16 expression and whilst this is absent on the HLA-DR^{lo} population, it is present on some of the HLA-DR^{hi} monocytes. Nonetheless this is on a minority of this subset of monocytes and appeared substantially upregulated upon differentiation into macrophages, so we believe CD16 in this context may be related to tissue residency rather than representing no- classical monocytes from the bloodstream.

21. Surprisingly the study failed to identify well characterised peak of monocyte/myeloid infiltration in the late secretory phase in human endometrium, which contribute in a major way to the destruction of the functionalis layer for shedding into the menstrual blood and its subsequent massive regeneration during the proliferative stage (ref 38, Salamonsen et al . PMID: 34486650 and refs therein). This fundamental difference between mouse and human was not mentioned. Additionally, technical issues (see pt 18 above) failed to reflect the relative numbers of EpCAM+ epithelial cells in human endometrium during the secretory stages, weakening enthusiasm for the importance of this study.

Our data does support studies showing an increase in macrophage numbers in the secretory phase in humans (Figure 1B) and a trend towards increased numbers of Mac2 macrophages in the late secretory phase (Figure 4G). As a note – neither do the unbiased single cell studies that have identified macrophages within the endometrium (as mentioned above, an example is Bunis et al (PMID 35069565) who found increased monocytes in the mid secretory phase of the cycle). A key difference between single cell studies of the human endometrium and ours, however, is the identification of cell subsets based on gene expression alone versus surface protein expression (as we have done). Unless we are mistaken none of the single cell papers have validated identification of their monocyte or macrophage subsets by flow cytometry, as in most cases myeloid cells were not the main focus of thsee studies.

The Salamonsen review (PMID 34486650) schematically summarises events through the cycle and immune infiltrates with a diagram representing more macrophages in the mid-late secretory phase but references a paper that in fact describes an increase in CD14^{+}HLA-DR^{+} cells (that they have called macrophages) in the early secretory phase as well as the late secretory phase of the cycle (Bulmer et al 1991 PMID 1757516).

The difference between mouse and human physiology in terms of shedding and menstruation has now been discussed in more detail in the discussion. As mentioned above, the epithelial cell issue has now been resolved and we have included additional analyses from our Hyperion data which reflect the physiological status of the tissue (increasing numbers of epithelial cells through the cycle; Figure 9C). Whilst this in itself

is known, this is important to contrast with Asherman Syndrome patients and the fact that epithelial cells are restored with surgery but the immune dysfunction is not.

22. Para 3 needs to be rewritten in the context that proestrus in mice is equivalent to the proliferative phase of the cycle and while the authors' showed infiltration in the early secretory stage of the cycle in human samples, this is not the peak of glandular epithelial proliferation, which occurs in the late proliferative stage and rapidly slows following ovulation due to the inhibitory effects of progesterone.
This has now been rewritten.

Both references 38 and 39 referred to in this paragraph demonstrate that apoptosis in human endometrium mainly occurs in from late secretory to early proliferative stages in controls, and also include the role of TNF. An additional ref that should be discussed is Santamaria et al Nat Commun PMID: 37735465, a scRNA seq study of endometrium from Asherman's syndrome patients.
We have included in the discussion the points about TNF being a candidate for mediating epithelial apoptosis. We have also included and discussed the Santamaria paper. Whilst we agree it is difficult to fully define exactly when and how monocytes cause epithelial apoptosis, our data do strongly indicate a role for them in epithelial cell homeostasis. The lack of epithelial cleaved caspase 3 in Ccr2$^{-/-}$ mice alongside abnormal expansion of epithelial cells and structures are consistent with an apoptotic mechanism in mice, and the clustering of high numbers of infiltrating monocytes in Asherman Syndrome near the epithelium with reduction in epithelial cell numbers also support this.

23. The Abstract will need revision to account for the above (point 22) and other changes required for this manuscript.
Abstract has now been re written.

24. Other important concepts not included in the Discussion are the role of the sex steroid hormones, estrogen and progesterone which overall regulate the cellular and molecular changes across the estrus and menstrual cycle. Monocytes and macrophages variously express nuclear estrogen receptor isoforms and progesterone receptors, including membrane bound GPCR (Pelekanou V et al 2016 PMID: 26394816; Murpy et al 2009 PMID: 19440537). Were these identified in the scRNAseq work? And what is their role in inducing the influx of monocytes into endometrium?
We have now added in data demonstrating expression of estrogen receptor isoforms and the progesterone receptor on our myeloid and CD45^{-} cell subsets in mice and in humans which has been included in the discussion (Extended data 19). Estrogen and progesterone receptors are expressed to some degree on myeloid cells but expression in both species is much higher on the stromal cells. Defining with certainty their role in the influx of monocytes into the endometrium is outwith the scope of this manuscript, but we have added a section into the discussion. Given the strong monocyte recruitment signature on the activated fibroblasts as inferred from the CellChat data (Extended data 11A), and the high expression of receptors in the stromal compartment, it is plausible that

steroid hormones may indirectly regulate the recruitment of monocytes to the endometrium via their effects on stromal cells. An obvious mechanism would be the induction of CCL2 (which attracts $CCR2^+$ monocytes) on stromal cells by estrogen (PMID 30838396). The peak of estrogen towards the end of the proliferative phase of the cycle would correspond to induction of CCL2 and subsequent recruitment of monocytes to infiltrate the uterus in the early secretory phase.

25. In summary, this is an interesting manuscript, well written and presented, that may contribute novel information on the role of monocytes in endometrial biology and endometrial diseases, but needs revisions to determine how significant the findings will be. It should be noted that there is a dearth of knowledge on endometrial and menstrual disorders which have massive effects on large numbers of women and this manuscript may well contribute new knowledge needed to further understand these conditions and stimulate further research that would benefit these women.
We thank reviewer 3 for these positive comments.

Reviewer #1

(Remarks to the Author)
The revised manuscript, even with the new data, remains largely a survey of monocyte and macrophage dynamics in the mouse uterus, with correlative data from normal humans and humans with Asherman syndrome. Its functional assessment of the importance of these cells in reproductive outcomes remains superficial and diffuse, with phenotypes (e.g. more collagen in Ccr2 KO mice) just described but not dissected. Other “mechanistic” experiments (a requirement for TGF-beta and IFNg in uterine macrophage/monocyte dynamics) are similarly superficial. There are some novel data, but these are relatively minor and do not push the reproductive biology nor the monocyte/macrophage fields forward in major ways. Continued and additional concerns:

We respectfully disagree that the manuscript is largely a descriptive survey or that the functional data are superficial. While prior studies have described uterine macrophage subsets and aspects of CCR2-dependent monocyte recruitment, our study is substantially more advanced by integrating macrophage ontogeny, subset-specific turnover, cytokine-dependent differentiation, epithelial and stromal tissue phenotypes, reproductive outcome, and human fibrotic disease. Thus, the manuscript does not simply catalogue monocyte/macrophage abundance across the cycle; it uses those dynamics as the foundation for perturbational, transcriptomic, spatial, tissue-structural, and translational analyses. To make this point clear – to our knowledge it has not even been shown before that uterine monocytes or macrophage can produce cytokine protein – a basic fundamental function of myeloid cells. Thus even these data alone advance the field; as such, knowledge regarding monocyte/macrophage biology in the uterus is decades behind the detailed functions and ontogeny studies studied at other mucosal sites such as the intestines and the lungs.

The significant advances beyond current knowledge are:

- Macrophage ontogeny in the cycling uterus: we show continual CCR2-dependent replenishment of uterine macrophages from circulating, bone marrow derived precursors.
- Subset-specific macrophage turnover: we define distinct inflammatory Mac1 and pro-repair Mac2 trajectories with different rates of replenishment from blood-monocytes.
- Cytokine regulation of macrophage-subset differentiation: we identify TGFβ and IFNγ as in vivo regulators of uterine macrophage differentiation states.
- Immune-structural crosstalk: we link CCR2-dependent monocyte recruitment to epithelial apoptosis/dynamics, fibroblast interactions, ECM remodelling, collagen accumulation, and reproductive outcome.
- Cross-species disease relevance: we show that these programs are conserved in human endometrium and demonstrate the significance when these pathways

are dysregulated e.g. in uterine fibrosis (in mice and humans), where inflammatory monocyte/Mac1 expansion persists before and after surgery.

The functional relevance of monocytes and macrophages in the uterus is indicated by the coordinated tissue phenotypes observed in Ccr2-deficient mice, including altered epithelial apoptosis and architecture, disrupted epithelial cell dynamics, increased collagen deposition, expansion of ECM-producing myofibroblasts, and reduced live pups per litter. We agree that these data do not prove a single linear causal pathway from ECM or epithelial changes to reproductive outcome, and we have revised the text to avoid such implication. However, together with the TGFβ-blockade and Ifnγr-deficiency experiments showing cytokine-dependent regulation of macrophage-state specification, and the human Asherman syndrome data showing persistent inflammatory monocyte/Mac1 expansion before and after adhesiolysis/surgery, the data provide an integrated cross-species framework linking monocyte recruitment, macrophage differentiation, epithelial/ECM homeostasis, reproductive outcome, and fibrotic endometrial disease. Thus the description of this study as a minor descriptive advance seems particularly inappropriate.

The manuscript still states: “monocyte-induced epithelial cell apoptosis” (page 12) and “cytotoxic properties of uterine monocytes and macrophages” (page 43), and this section, with its emphasis on GzmB and perforin expression, still gives the misleading impression that macrophages are killing uterine epithelial cells. The requested GzmB and perforin KO experiments were not performed. In their rebuttal, the authors seem to be arguing that they decided to keep including the GzmB and perforin data because of its inherent interest, but that’s not how it reads to me.

We understand the reviewer’s concern and have revised the results section title regarding these data to avoid any implication that direct killing of epithelial cells by monocytes/macrophages has been definitively demonstrated. Specifically, we have changed the title of this section from “monocyte-induced epithelial cell apoptosis” to **Cytotoxic profile of monocytes and regulation of uterine epithelial cell homeostasis.**

However, we respectfully disagree that the GzmB and perforin data are included only because of “inherent interest,” or that describing monocyte/Mac1 macrophages as having cytotoxic properties is unsupported. In this context, we use “cytotoxic properties” to refer to the expression of cytotoxic effector programs and molecules. Our transcriptomic analyses nominate cell-killing/cytotoxic pathways as a key feature of uterine monocytes and Mac1 macrophages in both mice and humans. Flow cytometry confirms enriched granzyme B and perforin protein expression in these populations compared with Mac2 macrophages; Ccr2-deficient mice, which lack uterine monocytes (and have reduced Mac1 macrophages), show markedly reduced epithelial cleaved caspase-3 during estrus. In human endometrium, GzmB-expressing HLA-DR^{+} cells localize near glandular epithelium. Together, these findings support a model in which recruited monocytes/Mac1 macrophages may contribute to epithelial cell apoptosis and turnover. We have also discussed the high constitutive amount of TNFα produced (unstimulated) by monocytes in both species which has been proposed

by others to be a key regulator of epithelial cell apoptosis in the uterus during reproductive cycles.

We agree that GzmB- or perforin-deficient mice would be required to demonstrate that epithelial apoptosis is directly mediated by these molecules. However given the timely and costly nature of these experiments, alongside the extensive amount of data already included in this manuscript, these are outwith the scope of this study.

In the text, we have distinguished three levels of evidence: first, CCR2-dependent loss of epithelial apoptosis in vivo; second, enrichment of cytotoxic effector pathways in monocytes/Mac1 macrophages; and third, spatial association of $GzmB^+$ myeloid cells with epithelial structures in human tissue. This is placed in the context of CCR2 dependent epithelial cell dynamics. We believe this preserves the biological relevance of the GzmB/perforin findings while avoiding overstatement of causality.

There is no page 43 of this manuscript.

Certain key results have already been published in PMID: 21825019 (J. Exp. Med. 2011). This paper showed that mouse uterine macrophages divide into two subsets, are greatly depleted from uterus in Ccr2 KO mice indicating a monocytic origin, and make chemokines that likely recruit monocytes. And in contrast to the results in Fig. 7C, Ccr2 KO mice have previously been shown to have normal litter sizes (PMID: 22278981; coincidentally(?) also from a Glasgow group), as I previously noted. Neither paper is cited and the discrepancy with PMID: 22278981 is not commented on but should be.

We thank the reviewer for highlighting Tagliani et al., which we have now cited. However, we fundamentally disagree that the key findings of our manuscript have already been published, since the Tagliani et al. study predominantly refers to pregnant mice which is a totally different physiological setting. In particular, the rapidly expanding myometrium during gestation (the abstract even mentions they have used the pregnant mouse uterus due to the rapid tissue growth). Pregnancy is not simply another stage of the reproductive cycle; it is a fundamentally different physiological and immunological state, involving implantation, decidualisation, trophoblast-derived signals, myometrial growth, vascular remodelling and fetal tolerance. In addition, the chemokines measured in this study (CCL2, CCL7 and CCL12) have been measured at tissue level, with only CCL2 assessed at cellular (macrophage) level and in any case does not overlap whatsoever with our assessment of inflammatory cytokine production (TNFα, IL-1β) or the wide range of other surface markers assessed.

There is a small section of the Tagliani paper discussing macrophages in the non-pregnant mouse uterus but the authors have claimed that there is minimal recruitment of monocytes to the non-pregnant uterus without showing any data to prove this. Instead, another paper is referenced (PMID 19546507) which involves characterisation of dendritic cells and does not have any information regarding monocyte recruitment to the uterus and subsequent differentiation into macrophages. Thus this paper in fact supports our point that prior to our paper, the ontogeny of macrophages in the non-

pregnant uterus and their replenishment from circulating monocytes has not been shown.

The distinction between our study and the Tagliani study is substantial:

- **Physiological context:** Tagliani et al. predominantly studied the pregnant uterus, particularly regarding monocyte recruitment and subsequent differentiation into macrophages. Our study examines the non-pregnant cycling uterus, where immune and structural cells fluctuate rhythmically across estrous/menstrual cycle stages.
- **Macrophage definition:** Tagliani et al. divided uterine macrophages into F4/80^{+}MHCII^{hi} and F4/80^{+}MHCII^{lo} populations. We define monocytes, intermediate cells, inflammatory Mac1 macrophages, and pro-repair/remodelling Mac2 macrophages in mice with different rates of turnover for the macrophage subsets using scRNA-seq, trajectory analysis, validated flow cytometry markers including Ly6C, MHCII, CD64, CD11c, CD206, MerTK, CX3CR1, CD24 and CD4 alongside cytokine production at protein level and proliferation and their fluctuations across the estrous cycle. In addition we provide human data in health and disease demonstrating homologous subsets with similar fluctuations across the reproductive (menstrual) cycle despite the differences in physiology. In both species we provide detailed analyses of inferred function from transcriptomic analyses and pathway analysis, actual function by measuring cytokine production and we also explore the role of monocytes in particular in regulating ECM and epithelial cell homeostasis
- **Central mechanism:** Tagliani et al. focused on CSF-1-dependent proliferation, monocyte extravasation and CCR2-ligand production in the pregnant myometrium. Our study identifies CCR2-dependent monocyte replenishment of macrophage subsets, differential Mac1/Mac2 turnover from monocytes, and TGFβ- and IFNγ-dependent regulation of macrophage-state specification in the cycling uterus.
- **CCR2-dependence and monocyte recruitment:** Tagliani et al. showed CCR2-dependent Ly6C^{hi} monocyte entry into the pregnant uterus and reduced abundance of some macrophage compartments, particularly MHCII^{hi} macrophages. This is not equivalent to the reviewer's broad claim Tagliani et al. show all uterine macrophages are greatly depleted in Ccr2-deficient mice, nor does it define the cycling monocyte/Mac1/Mac2 system described here. By assessing the macrophage populations identified in a non-biased fashion by scRNAseq, we confirm that although total macrophage numbers are indeed lower in *Ccr2*$^{-/-}$ mice but that this is much more of a loss of the pro-inflammatory Mac1 macrophages, fitting with our chimera data showing that Mac1 macrophages are more dependent on monocyte replenishment and turned over more quickly than Mac2 macrophages. We would also like to make the point that macrophages in non pregnant *Ccr2*$^{-/-}$ mice in the Tagliani paper were only assessed during diestrus whereas we have information at all cycle stages.
- **Functional tissue outcomes:** Tagliani et al. did not assess epithelial apoptosis/dynamics, epithelial architecture, fibroblast activation, myofibroblast

expansion, ECM remodelling, collagen accumulation in relation to cycling uterine monocyte dynamics. These are central findings of our manuscript.

- **Human disease relevance:** Tagliani et al. did not address human endometrium or endometrial fibrosis. Our study builds on the mouse findings to human cycling endometrium and Asherman syndrome, where inflammatory monocyte/Mac1 macrophage expansion persists before and after surgery.

Thus, Tagliani et al. provides useful precedent that CCR2-dependent monocyte entry can occur in the pregnant uterus, but it does not address the central question of our manuscript: whether/how monocytes are recruited to the cycling uterus to replenish macrophages, how these subsets regulate immune-structural cell crosstalk in the non-pregnant cycling uterus and how disruption of this system relates to epithelial/ECM abnormalities, reproductive outcome and human endometrial fibrosis.

We have also revised the manuscript to explicitly discuss the apparent discrepancy with Menzies et al. (PMID 22278981). The reviewer is correct that Menzies et al. reported no reduction in litter size in Ccr2-deficient mice, with median litter sizes of 7 pups in WT mice and 8 pups in Ccr2-deficient mice, based on 12 WT and 10 Ccr2-deficient mothers. However, their endpoint was not identical to ours: Menzies et al. euthanized pregnant females on D18 or after delivery of the first pup, and litter sizes were pooled from D18 and laboring mice. Thus, their analysis assessed litter/fetal number in established late pregnancy and at entry into labour, whereas our Fig. 7C quantified **live** pups at first inspection after delivery. Their design would not necessarily detect losses occurring during completion of labor, stillbirth/peripartum loss, or reduced liveborn output after the first pup was delivered. In addition, our analysis included almost twice as many litters, with 20 WT and 23 $Ccr2^{-/-}$ litters, and showed an approximately 2–3 pup reduction in live pups per litter in $Ccr2^{-/-}$ mice. We have revised the text to acknowledge this discrepancy directly.

There are inappropriate logical elisions. For example, while CCR2 deficiency apparently leads to breeding abnormalities as well as changes in the uterine ECM and epithelial cells, the manuscript implies that the breeding abnormalities are *due* to the uterine ECM and epithelial cell changes. This is not established, and many other possibilities exist (aside from the discrepancy between the breeding defect seen here and PMID: 22278981). Perhaps there is an ovulation defect.

We agree with the reviewer that the reduced number of live pups per litter in $Ccr2^{-/-}$ mice should not be interpreted as being definitively caused by the epithelial and ECM abnormalities described in the uterus.

However, we do not agree that these observations are unrelated or merely coincidental. In $Ccr2^{-/-}$ mice, loss of uterine monocytes and reduction of Mac1 macrophages are accompanied by coordinated uterine tissue abnormalities, including altered epithelial apoptosis/dynamics, abnormal epithelial architecture, increased collagen deposition,

and expansion of ECM-producing myofibroblasts. These data support the conclusion that CCR2-dependent monocyte recruitment is linked to uterine tissue integrity, and that disruption of this pathway is associated with impaired reproductive output, measured as fewer live pups per litter.

We have therefore revised the manuscript to soften language implying a direct causal chain from CCR2-dependent monocyte loss to epithelial/ECM changes to breeding outcome. We now explicitly acknowledge that, alongside altered uterine tissue integrity/remodelling, other indirect mechanisms may contribute to the reduced live pups per litter in $Ccr2^{-/-}$ mice, including ovarian factors or ovulation defects.

Fig. 8A collagen area (resubmission) has different data for the older mice than that in the original (Fig. 6A).

We have gone through the datasets and the staff member carrying out the experiment had accidentally included some different regions of interest from the same sample as different data points, which has now been rectified.

Fig. 6F remains problematic. Why are all the endothelial cells massively expanded as well? How were absolute epithelial cell numbers determined? This is not a trivial endeavor unless precise amounts of tissues are run through the cytometer or counting beads were used. I do not see mention of this.

We thank the reviewer for raising this point. Endothelial cells are not a major focus of the manuscript and we do not draw mechanistic conclusions from their expansion in Fig. 6F. They were included as part of the broader $CD45^{-}$ structural-cell flow cytometry panel to provide a complete overview of tissue-cell composition alongside epithelial cells, fibroblasts and myofibroblasts.

Absolute epithelial and endothelial cell numbers were determined from whole uterine digests, with tissue input standardised across samples: both uterine horns were collected, enzymatically digested, filtered and analysed by flow cytometry. Thus, the reported values represent cell numbers in figure 6 recovered per whole digested uterus rather than percentages alone. We have revised the Methods and figure legend to make this explicit and to clarify how absolute cell numbers were calculated.

We agree that the endothelial-cell changes may be biologically interesting, but they are outside the central scope of the current study and would require dedicated vascular analyses. We have therefore avoided overinterpreting this finding. The main conclusion from Fig. 6F is that epithelial cell dynamics are altered in $Ccr2^{-/-}$ mice, consistent with the loss of epithelial cleaved caspase-3 and abnormal epithelial architecture shown in Fig. 6C–E.

I'm not convinced from Fig. 6H and ED 9B the HLA-DR+ cells are the ones that express Granzyme B. The abutting of the gland seems only to be in one spot.

Thank you for raising this point. We would highlight that we are not asking the reader to rely solely upon this image for determination that HLA-DR+ cells are those expressing Granzyme B. While the imaging provides spatial context we show, consistently, across species and at both gene and protein levels this is the case:

-Mouse scRNA-seq shows expression of Gzmb and Prf1 in uterine myeloid clusters, with cytotoxic/cell-killing programs enriched in monocytes/Mac1 macrophages.

-Human sorted bulk RNA-seq similarly identifies cytotoxicity-associated genes, including GZMB, PRF1, GZMA, GZMK and NKG7**,** in the Mac1 axis rather than Mac2 macrophages.

-Most importantly, flow cytometry directly confirms Granzyme B and perforin protein expression in mouse and human monocyte/macrophage subsets.

Thus, although we agree that the representative imaging should not be used alone to infer that every Granzyme B+ cell is HLA-DR+ or that gland-associated localization is uniform, the broader conclusion that uterine monocyte/Mac1-lineage cells express Granzyme B/perforin-associated cytotoxic effector programs is well supported.

The justification not to assess estrogen levels only in diestrus and not across the entire estrous cycle is that diestrus is when there is the difference between progesterone levels in Ccr2 KO mice versus controls. This is not a compelling argument. Also, I was concerned not that altered hormones are changing monocyte/macrophage dynamics but rather that these altered dynamics were altering hormone levels. Which they appear to do at least for P4 in diestrus.

Upon discussion with the editor prior to the first resubmission it was agreed that it would be acceptable to show progesterone only, given the well-known difficulties in measuring circulating estradiol in serum in mice. Thus we have now removed the estrogen data entirely.

We agree that the difference in progesterone levels during diestrus in $Ccr2^{-/-}$ mice lead to the possibility that loss of monocytes and skewed macrophage subsets may affect hormone levels. This has now been incorporated into the discussion. However the majority of epithelial cell changes occur during estrus where the wildtype mice have expansion of epithelial cell numbers, and the collagen deposition occurs during proestrus. At both these cycle stages the levels of progesterone in $Ccr2^{-/-}$ mice are comparable to wildtype, indicating these abnormalities in tissue structure are unlikely to have arisen from a difference in progesterone levels at a different time in the cycle.

It remains problematic to score cell abundance as percentages of CD45- or CD45+ cells.

We have previously addressed this point by adding in absolute numbers for all figures requested in the previous review. Thus, where our interpretation depends on expansion or depletion of cell populations, we provide absolute cell numbers alongside proportions or present absolute numbers directly.

For example:

- **Cycle-dependent monocyte/macrophage abundance:** in Fig. 1A and Fig. 1B, mouse and human monocyte/macrophage dynamics across reproductive cycle stages are shown as both proportions and absolute numbers, supporting the conclusion that monocytes are the most dynamically regulated myeloid population.
- **$Ccr2^{-/-}$ genotype-dependent myeloid changes:** for uterine monocytes and macrophage subsets in $Ccr2^{-/-}$ mice, the relevant analyses include absolute cell numbers when making claims about loss or reduction of populations.
- **Epithelial-cell dynamics:** Extended Data Fig. 8H shows EpCAM+ epithelial cells across the estrous cycle as both proportions of CD45- cells and absolute numbers, supporting the conclusion that epithelial-cell abundance changes across cycle stage.
- **Asherman syndrome monocyte/macrophage abundance:** Fig. 10C–F shows proportions and absolute numbers for HLA-DR^{lo} monocytes, HLA-DR^{hi} monocytes, Mac1 macrophages and Mac2 macrophages, supporting the abundance claims in human endometrial fibrosis.
- **Older/fibrotic mouse uterus:** Extended Data Fig. 15D–G presents absolute numbers for epithelial cells, monocytes, Mac1 macrophages and Mac2 macrophages, supporting the interpretation of altered tissue and immune-cell abundance in aged fibrotic uteri.

By contrast, where percentages are shown without absolute numbers, they are used appropriately to address relative composition, subset balance, or marker expression within subsets, rather than absolute abundance. Examples include proportions of monocytes or macrophage subsets expressing markers such as F4/80, Ki67, CD4, CD86, MerTK, or producing cytokines such as TNFα, and proportional imaging readouts such as epithelial fraction within tissue sections. These are questions of phenotype, subset skewing, or tissue composition, for which percentages within a defined parent gate are the appropriate metric.

We therefore do not agree that the manuscript relies inappropriately on percentages of CD45+ or CD45- cells to support abundance claims. We have reviewed the figure legends to make sure that it is clear whether data are representative of proportions within a parent gate or absolute numbers.

Collagen “area” remains an inappropriate measure of collagen content.

We have previously addressed this point in response to the same comment in the last review by performing hydroxyproline quantification for the key $CCR2^{-/-}$ finding (Fig 7b), as suggested, where we make the strongest claim about genotype dependent effects on collagen content. We have also carried out hydroxyproline assays in the young versus older mice (Fig 8b).

All of the Asherman data is correlative and observational, and interpretations are speculative.

Human Asherman syndrome samples are necessarily observational, as sampling is invasive and yields relatively small amounts of tissue. However, the interpretation is not speculative with the disease analysis following directly from a mouse-human conserved cell-state framework established earlier in the manuscript:

- **We first define the relevant cell states in mouse:** uterine monocytes, inflammatory Mac1 macrophages and pro-repair/remodelling Mac2 macrophages.
- **We then show these states are conserved in humans:** FACS-sorted human endometrial monocytes, Mac1 macrophages and Mac2 macrophages show conserved transcriptional programs, with the Mac1 axis retaining inflammatory/cytotoxic features and Mac2 macrophages retaining tissue-remodelling/pro-repair features.
- **We then test this framework in Asherman syndrome:** the disease is not associated with a generic macrophage change, but with expansion of the inflammatory monocyte/Mac1 axis, including increased $HLA\text{-}DR^{lo}$ monocytes and Mac1 macrophages by flow cytometry.
- **This immune skewing aligns with tissue pathology:** Asherman samples show collagen deposition, impaired glandular epithelial Ki67, reduced epithelial abundance pre-surgery, and persistence of inflammatory CD206-HLA-DR+ monocyte/macrophage populations after surgery.

We would argue that this is actually a deep level of human and disease tissue integration, where we demonstrate conservation of important biological findings in human and relevance to specific disease states.

Blurry figures (e.g. 7D, ED 8B, and many other places, but next to figures that are sharp so this is not a download issue), inconsistent formatting (e.g. ED 8F&H), inappropriately placed figures (ED 9D), ED 19 is not called out in the Results section. Multiple references are missing (the text just shows a “ref” placeholder). These errors raise concerns about the level of attention to detail.

The blurring of some figures occurred during the transfer of all figures to PDF, which was done for ease of review, which has now been amended for the mentioned figures for this round of review. If accepted, all figures will be higher resolution and sharp, at the appropriate dpi. We are unsure what the reviewer means re inconsistent formatting

(ED 8F&H) and inappropriate placement of figures (ED 9D). Extended data 19 is not part of the results because it is only relevant to the discussion, and was added into the discussion at the request of another reviewer. We have now included the missing references.

The staining in Fig ED 9C looks artefactual.

These immunofluorescence images are supportive data for Hyperion IMC analysis, performed at the request of another reviewer. These immunoflourescence images are consistent with the Hyperion images as another detection method re tissue localisation (see figure 6H). Further, the detailed distribution and appearance is entirely consistent with secreted cytotoxic granules- a good example can be seen in Figure 2A of "Granzyme B PET Imaging as a Predictive Biomarker of Immunotherapy Response", PMID: 28461564 that demonstrates appearance of secreted and intracellular granzyme B. These findings are completely absent in isotype control in our study (shown), conflicting with the artefact statement, and consistent with standard and robust scientific practice in immunofluorescence imaging.

I am unclear here what aspect the reviewer is unconvinced by, but we are happy to provide additional imaging if required but would need specific guidance on what further evidence would be helpful to the reviewer.

Reviewer #2

(Remarks to the Author)
The authors have added a substantial amount of data and clarification to this new version of their manuscript. My concerns were addressed in a satisfactory manner. One aspect that I would appreciate more clarity on is the differentiation of human monocytes into HLA-DR low and high cells. The gating strategy in extended figure 2 somewhat suggests that HLA-DR low monocytes might be excluded from the analysis shown in Figure 1. Figure 5 shows all in all much less of a fluctuation of monocytes across the cycle, even if gated on the HLA-DR low monocytes, which are supposed to be the ones changing in abundance. Can the authors explain this discrepancy?

We thank reviewer 2 for these positive comments. We have now changed our example gating which we have now realised was not fully representative (Extended data 2a), as usually the gate was set to include all HLA-DR+ cells including high and low expressers. We believe the discrepancy in the total monocyte fluctuations versus the HLA-DR^{lo} subset fluctuations is due to the likely combined contribution of both (HLA-DR^{hi} and HLA-DR^{lo}) subsets. Although the effect was more striking in the HLA-DR^{lo} subset, the HLA-DR^{hi} analyses shows several samples that are high during proestrus. Thus when these data are combined for total monocytes, the effect is more obvious, as in Figure 1.

Another aspect concerns the interpretation of the extensive expression changes of MerTK, CD86, and CD4 on monocytes across the cycle. They seem fairly absent during the proestrus, when monocytes are abundant, and very high in the diestrus, when there are almost no monocytes remaining. Is it still accurate to call these cells monocytes? The gating in Figure 1 A suggests that there are none, which would explain the data, potentially.

We thank the reviewer for pointing this out and we agree: since there are virtually no monocytes in diestrus, the very small number of cells present should not be used for gating on and assessing expression of markers within that subset. Monocytes in diestrus have now be excluded from the analyses. MerTK, CD86 and CD4 – although commonly expressed on macrophages – are not restricted to macrophage or macrophage-specific markers and more are indicators of function or activation. There is a common misconception that CD86 is an "M1/pro-inflammatory" macrophage marker but monocytes and pro-repair macrophages can also be highly activated (and thus express CD86 as in other mucosal tissues). We think on the monocytes this represents fluctuations in activation levels throughout the cycle (barring diestrus where we agree the few cells present should not be analysed). MerTK is involved in apoptotic cell uptake and in this context it is not surprising it is expressed on monocytes given we make the point later that monocytes in the uterus express many genes involved in tissue remodelling.

References are missing in 6 cases in the manuscript, where it reads '(ref)'.

These have now been added in.

Reviewer #3

(Remarks to the Author)
The authors have undertaken a thorough revision of their manuscript, undertaking further experiments, and in general have addressed all the points I raised. There are a couple of minor issues remaining or have arisen as a result that need to be addressed, although there is still some misunderstanding on differences between the cyclic changes in mouse and human endometrium.

We thank reviewer 3 for these positive comments.

1. Here are the differences and similarities of the estrus and menstrual cycles. The WOI occurs in fertile women in all ovulatory cycles during the mid-secretory stage when endometrial decidualisation spontaneously occurs, while there is no WOI in cycling mice as the ovulated oocytes need to be fertilised to induce endometrial stromal decidualisation. Thus the WOI occurs on day 4-5 of pregnancy in mice. It can be simulated experimentally by providing a decidualisation stimulus – oil droplet injection into the uterine horn or mating with a castrated male mouse (pseudopregnancy). This is why mice do not menstruate as decidualisation is a terminal differentiation of the

human endometrium which needs to be removed to enable endometrial regeneration as a new cycle commences. The authors should highlight these differences and point out that the monocyte differentiation is a similar process in both humans and mice, despite the differences in cycle/pregnancy dynamics. Accordingly, the WOI cannot be used to cover both mouse and human in the Abstract as the manuscript currently stands. The authors would need to induce WOI in pseudopregnant mice to demonstrate the monocyte/Mac role in uterine remodelling during decidualisation to show they mimic the same role in human WOI. It would also still be useful to plot the cycle stages on the graphs as per convention to commence with proestrus.

We apologise for our misunderstanding in the physiological difference between mouse and human reproductive cycles and thank the reviewer for this detailed information which has now been clarified in the manuscript. Specifically, we have highlighted these differences, and in particular that there is no WOI in cycling mice. The abstract as well as the introduction, results and discussion have been appropriately amended. We have pointed out as suggested that monocyte differentiation is a similar process in mice and humans despite the physiological differences between species.

We have also replotted all graphs and images regarding estrous cycle stages to commence with proestrus.

2. In the Introduction (p3, 1st para) please add in the secretory differentiation of the glandular epithelial cells ie "...the endometrium undergoes cyclical episodes of proliferation (estrogen-dominated proliferative phase of the menstrual cycle) followed by differentiation of the epithelium into secretory cells and fibroblasts into decidual cells (progesterone-dominated)."

This has now been added in.

3. Fig 8A/Ext data 15A – "A thinner epithelial layer surrounding glands (glandular epithelium) etc" Are the authors describing the glandular epithelium which changes in height – highest/thickest during estrogen-dominant phase and lower in other phases, particularly low estrogen. Endometrial glands are lined with a single layer of pseudostratified columnar epithelium Could the authors be seeing lower epithelium in fewer glands in a thinner endometrium in older mice? Clarify the wording.

This wording has now been clarified and the text amended.

4. Fig 9 F, G. What does "case average" mean in the Y axis label – perhaps clarify in the Fig legend.

Case average refers to the fact that 5 regions of interest per sample were acquired, and each data point represents the average of those 5 different images. This has now been clarified in the figure legends.

5. P 16 second to last sentence – Mac1s ae also sustained at a high level in the proliferative stage (Fig 10H) but '(Fig 10G)'.should also be referenced for the monocytes.

This has now been amended in the text.

6. Discussion p 18 3rd para – the marked reduction of glandular epithelial proliferation is also driven by progesterone, and the secondary effect could also be due to the infiltration of monocytes that induce apoptosis. This may be of greater importance in mice as they remodel the endometrium rather than shedding it as in humans.

This has now been incorporated in the discussion.

7. Discussion p 19. Was ESR2 found in your data set on monocytes and/or Mac1s?

ESR2 was not expressed on the monocytes or macrophages which is why it is not shown but this has now been clarified in the discussion.

8. Discussion p 20 1st para – delete "(and thus epithelial layers)"

This has now been amended.

9. There are numerous places in the new text showing "(ref)". Please insert the relevant references.

These references have now been added in.
